# Physicochemical Characterization of a Co-Amorphous Atorvastatin-Irbesartan System with a Potential Application in Fixed-Dose Combination Therapy

**DOI:** 10.3390/pharmaceutics13010118

**Published:** 2021-01-18

**Authors:** Marcin Skotnicki, Barbara Jadach, Agnieszka Skotnicka, Bartłomiej Milanowski, Lidia Tajber, Marek Pyda, Jacek Kujawski

**Affiliations:** 1 Chair and Department of Pharmaceutical Technology, Poznan University of Medical Sciences, 60-780 Poznan, Poland; bajadach@ump.edu.pl (B.J.); askotnic@ump.edu.pl (A.S.); bmilan@ump.edu.pl (B.M.); 2 School of Pharmacy and Pharmaceutical Sciences, Trinity College Dublin, College Green, 2 Dublin, Ireland; ltajber@tcd.ie; 3 Department of Biophysics, Poznan University of Medical Sciences, 60-780 Poznan, Poland; mpyda@utk.edu; 4 Department of Chemistry, Rzeszow University of Technology, 35-959 Rzeszow, Poland; 5 Chair and Department of Organic Chemistry, Poznan University of Medical Sciences, 60-780 Poznan, Poland; jacekkuj@ump.edu.pl

**Keywords:** irbesartan, atorvastatin, co-amorphous system, differential scanning calorimetry, physical stability, intrinsic dissolution rate, density functional theory

## Abstract

The aim of this study was to characterize a 1:1 molar ratio of a pharmacologically relevant co-amorphous atorvastatin-irbesartan (ATR-IRB) system obtained by quench cooling of the crystalline ATR/IRB physical mixture for potential use in the fixed-dose combination therapy. The system was characterized by employing standard differential scanning calorimetry (DSC), Fourier transform-infrared spectroscopy (FT-IR), and intrinsic dissolution rate studies. Quantum mechanical calculations were performed to obtain information regarding intermolecular interactions in the studied co-amorphous ATR-IRB system. The co-amorphous formulation showed a significant improvement in the intrinsic dissolution rate (IDR) of IRB over pure crystalline as well as its amorphous counterpart. An unusual behavior was observed for ATR, as the IDR of ATR in the co-amorphous formulation was slightly lower than that of amorphous ATR alone. Short-term physical aging studies of up to 8 h proved that the ATR-IRB co-amorphous system remained in the amorphous form. Furthermore, no physical aging occurred in the co-amorphous system. FT-IR, density functional theory calculations, and analysis of *T*_g_ value of co-amorphous system using the Couchman–Karasz equation revealed the presence of molecular interactions between APIs, which may contribute to the increased physical stability.

## 1. Introduction

The principal cause of death in developed countries is attributed to cardiovascular diseases (CVDs). CVDs are the disorders of heart and blood vessels, including heart failure, coronary heart disease, hypertension, cerebrovascular disease, or congenital heart disease [1].

High blood pressure, as well as low-density lipoprotein cholesterol, increased levels of serum homocysteine, and platelet aggregation resulting from smoking, lack of exercise, and poor eating habits are considered as key risk factors for CVDs. These factors frequently co-exist and increase the risk of diseases described above. One of the recently implemented strategies to treat CVDs is to address multiple risk factors simultaneously, for example, by introducing a fixed-dose combination (FDC), i.e., a combination of drugs in one dosage form [1]. Such a combination may contain a statin and a blood pressure lowering agent.

Atorvastatin (ATR) belongs to inhibitors of 3-hydroxy-3-methyl-glutaryl-coenzyme A (HMG-CoA) reductase. The mechanism of action is the catalysis of the conversion of HMG-CoA to mevalonate in cholesterol biosynthesis. ATR is administered orally for the treatment of hypercholesterolemia. Irbesartan (IRB) is a specific competitive antagonist of the angiotensin II receptor (AT_1_ subtype) of the nonpeptide type and, like ATR, is given orally for the treatment of hypertension or heart failure. ATR and IRB in a fixed-dose combination formulation have already been proven to deliver significant benefits for the patients [2]. ATR and IRB belong to class II of drugs according to the Biopharmaceutics Classification System (BCS), i.e., have low solubility and high permeability [3,4,5]; thus, the solubility and the dissolution rate of IRB and ATR are considered as key features limiting their oral bioavailability. ATR is currently used in formulations in a crystalline or an amorphous form [6], whereas IRB is formulated as a crystalline form. Two polymorphic forms, i.e., form A and form B of IRB have been distinguished, with form A being the better soluble form in comparison to form B, and the former is formulated as solid dosage forms [7]. It has also been established that IRB shows high variability in terms of oral bioavailability [8].

Various methods to improve solubility and also dissolution rates have been proposed and evaluated [5,9]. One of them is the use of an amorphous form of a drug instead of the crystalline phase [10]. In the amorphous state, the dissolution process can be faster due to vastly diminished intermolecular interactions [11]. On the other hand, amorphous materials may recrystallize upon storage, and this is because the internal energy of amorphous solids is higher compared to their crystalline counterparts. It is well known that the interactions between the molecules are stronger within the crystal lattice [12]. One of the methods to overcome this stability issue is to use polymers to form polymeric amorphous solid dispersions (PASD) [13]. However, the use of polymers in PASD has many disadvantages [13]. Their application is limited due to long-term stability issues and processing into dosage forms [13]. Polymers used in PASDs are often hygroscopic. Thus, water sorbed during storage or processing may act as a plasticizer, reducing the glass transition temperature, leading to phase separation and recrystallization [14]. In addition, limited miscibility of the drugs with polymer necessitates a high amount of polymeric material to be combined with the API, increasing final dosage form, especially for high dose drugs [15].

There is an interesting alternative to PASD without the aforementioned drawbacks. The improvement in the dissolution rate and stability of amorphous APIs can be achieved by combining amorphous drugs into one single-phase system called a co-amorphous system (CAM) [16,17]. This approach has become regarded as promising, especially in relation to the forming of drug-drug CAM, which can be administered as a combination therapy (FDC). The reported co-amorphous systems have been found to increase both the dissolution rates and stability of the component APIs [16,17,18,19]. In some cases, those phenomena have been attributed to molecular interactions between the two drugs present in the system [12,13,14]. The identification of possible intermolecular interactions between APIs and their impact on stability and dissolution is critical during the formulation of amorphous binary systems [1,16,17]. The following phenomena have been suggested as potentially responsible for the enhanced stability: anti-plasticization effect (elevation of glass transition temperature) and reduced molecular mobility as well as intermolecular interactions between the APIs in the system [20,21,22,23,24,25,26,27].

The aim of this study was to characterize the 1:1 molar ratio of a co-amorphous API-API system obtained by quick cooling of the physical mixture melt for the potential use in an FDC. Atorvastatin and irbesartan were chosen as model compounds to formulate the co-amorphous system. There have been several attempts to improve the solubility of ATR and IRB by both solid dispersion [3,5,28,29,30,31] and co-amorphous formulation [27,32,33,34,35]. ATR and IRB were chosen as model compounds for several reasons. Both are poorly soluble BCS class II drugs [3,4,5,6]. Amorphous IRB has a relatively low glass transition temperature (*T*_g_) and undergoes a physical aging process at 25 °C (becomes more structurally ordered with time) [36], which may potentially change its physicochemical properties [37,38,39]. ATR has a high *T*_g_ [6], which may be used to elevate the low *T*_g_ of IRB (anti-plasticization effect) in the co-amorphous combination. Both ATR and IRB are strong glass-forming agents [6,36] with meting points close to each other, i.e., 160–185 °C and are thermally stable at this temperature, thus, it is possible to formulate them into co-amorphous phases using the fast cooling of the melt method. The simultaneous administration of both APIs as an FDC has already been demonstrated as being beneficial for the patients [2].

The co-amorphous system was characterized using a panel of techniques including thermogravimetric analysis (TGA), standard differential scanning calorimetry (DSC), Fourier transform-infrared spectroscopy (FT-IR), and intrinsic dissolution rate studies. Quantum mechanical calculations were also performed to evaluate the possible intermolecular interactions in the co-amorphous ATR-IRB complexes, to ascertain the most favorable formations and support the outcomes of the dissolution studies.

## 2. Materials and Methods

### 2.1. Materials

**Figure 1:**
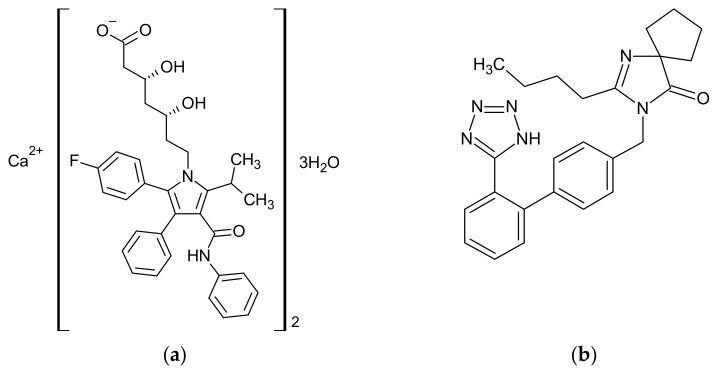
Chemical structures of (**a**) atorvastatin calcium trihydrate (*M*^ATR^ = 1209.4 g mol^−1^) and (**b**) irbesartan—1*H* tetrazole tautomer (*M*^IRB^ = 428.5 g mol^−1^).

Atorvastatin (ATR; crystalline; in the form of trihydrate, pharmaceutical-grade; Figure 1a) was obtained from Biopharm, Poznań, Poland and used “as is”. Its amorphous form (form AM) was prepared immediately prior to characterization in situ in the DSC pan or on a hot plate (Magnetic stirrer RCT basic, IKA-Werke GmbH & Co. KG, Staufen, Germany) by heating the sample on aluminum foil to 185 °C, holding for 5 min followed by cooling at approximately 5 °C min^−1^ to room temperature.

Irbesartan (IRB, crystalline; pharmaceutical-grade; Figure 1b) was obtained from Polpharma, Stargard Gdański, Poland and used “as is”. Its amorphous form (form AM) was prepared immediately prior to characterization in situ in the DSC pan, or on a hot plate (Magnetic stirrer RCT basic, IKA-Werke GmbH & Co. KG, Staufen, Germany) by heating the sample on aluminum foil to 185 °C, holding for 5 min followed by cooling at approximately 5 °C min^−1^ to room temperature.

### 2.2. Preparation of Amorphous Binary Systems

Physical mixtures of 1:1 mol/mol IRB/ATR (73:27 *w*/*w*) and 50:50, 80:20, and 20:80 (*w*/*w*) were prepared by mixing APIs in a glass mortar with a piece of plastic film for 20 min. Co-amorphous samples were prepared by heating the binary physical mixtures immediately prior to characterization in situ in the DSC pan to 185 °C and cooling to 20 °C at a 10 °C min^–1^ rate, or on a hot plate (Magnetic stirrer RCT basic, IKA-Werke GmbH & Co. KG, Staufen, Germany) by heating sample on the aluminum foil to 185 °C, holding for 5 min followed by cooling at approximately 5 °C min^−1^ to room temperature.

### 2.3. Thermogravimetric Analysis

TGA curves were obtained using a Mettler-Toledo TGA/DSC1 instrument or a Perkin Elmer Pyris 1 TGA under a nitrogen gas flow 60 mL min^−1^. Powdered samples weighing 2–20 mg were placed in an opened ceramic pan and heated at a rate of 10 °C min^−1^ from 25 to 600 °C.

### 2.4. Differential Scanning Calorimetry

DSC curves were obtained using a DSC Q1000 TA Instrument Inc., v9.9 Build 303 (New Castle, DE, USA) or a DSC 821 Mettler-Toledo (Greifensee, Switzerland) under a nitrogen gas flow of 50 and 60 mL min^−1^, respectively. Sample (1–10 mg) were crimped in a hermetic aluminum pan and heated at 10 °C min^−1^ from 20 to 190 °C. Next, the samples were cooled down at 10 °C min^−1^ to 20 °C and reheated to 190 °C in a second run.

The enthalpy relaxation change after isothermal physical aging was determined as a function of aging time. The amorphous samples were first heated to 190 °C to eliminate the effect of prior thermal history and then cooled at 10 °C min^−1^ below the glass transition temperature to the aging temperature (*T*_a_ = 45 °C). The samples were kept at *T*_a_ for 1 and 8 h, and then cooled at 10 °C min^−1^ to 20 °C. A subsequent heating scan at 10 °C min^−1^ to 190 °C provides the data for the aged samples.

The temperature and enthalpy were calibrated with indium (m. p. = 156.65 °C, Δ_fus_*h* = 28.45 J g^−1^) [40], and at least two measurements were completed for each sample. Melting was quoted as an onset temperature. Glass transition temperature was quoted as the midpoint of heat capacity between the liquid and glassy states [40]. All values were determined using TA Universal Analysis 2000 v4.5A (New Castle, DE, USA) or Mettler-Toledo STARe SW v10.0 (Greifensee, Switzerland) software. Errors are quoted as one standard deviation.

### 2.5. Determination of the Theoretical T_g_ Value Using Couchman-Karasz Equation

The experimental value of glass transition for the co-amorphous system was compared with the predicted *T*_g_ calculated from the Couchman–Karasz equation [41]:

(1) $$T_{g12}=\frac{w_{1}T_{g1}+Kw_{2}T_{g2}}{w_{1}+\;Kw_{2}}$$

where *T*_g12_ is the *T*_g_ of the co-amorphous mixture, *T*_g1_ and *T*_g2_ are the *T*_g_ of the individual components, *w*_1_ and *w*_2_ are the weight fractions, and *K* is a constant and is given by the equation:

(2) $$K=\frac{\Delta c_{p2}}{\Delta c_{p1}}$$

where Δ*c*_p_ is the change in the heat capacity at *T*_g_.

### 2.6. Fourier Transform-Infrared Spectroscopy Measurements

Fourier transform infrared spectroscopy was carried out using an Alpha Bruker FT-IR spectrometer (Billerica, MA, USA) in KBr pellets. Spectra were recorded at room temperature from 4000 to 500 cm^−1^ collecting 64 scans with a resolution of 2 cm^−1^.

### 2.7. Computational Section

The initial structures of IRB and ATR to generate the ATR-IRB complexes were taken from the Cambridge Structural Database (CCDC), structure NOZWII for IRB [42], and structure IZOQIZ for ATR [43]. For quantum-mechanics calculations, the density functional theory (DFT) formalism was applied [44]. The density functional calculations were carried out and optimized for four complex geometries at the DFT level of theory using the Gaussian G16 A.01 software (Gaussian Inc., Wallingford, CT, USA) [45] and very tight criteria as well as the most commonly used global hybrid generalized gradient approximation B3LYP functional (B3LYP/6-31G(d,p) approach with additional keywords “empiricaldispersion = gd3bj iop (3/174 = 1000000, 3/175 = 1988900, 3/177 = 398100, 3/178 = 4421100)” in the input file) [46] and a pure functional, B97, with the Grimme’s D3BJ dispersion (B97D3/6-31G(d,p) approach with additional keywords “empiricaldispersion=gd3bj” in the input file [47] in the gaseous phase were used. The ideal gas, rigid rotor, and harmonic oscillator approximations were applied to calculate the vibrational frequencies and thermodynamic properties. No imaginary frequencies within the vibrational spectra were obtained and therefore confirmed the energy minimum for all compounds (ATR calcium and IRB as tetrazole tautomer 1*H* and 2*H*) and ATR-IRB complexes (Cartesian coordinates of all considered compounds are given in the Supplementary Materials). The interaction energy of all optimized complexes was calculated by applying the counterpoise corrected (CP) method [48] based on the basis set superposition error (BSSE) [49] at B3LYP/6-311++G(d,p) or B97D3/6-311++G(d,p) levels of theory. The complexes with the lowest resulted interaction energy were analyzed by applying molecular electrostatic potential (MEP) computations together with Quantum Theory Atoms-In-Molecules (QTAIM) approach at B3LYP/6-311++G(d,p) or B97D3/6-311++G(d,p) levels of theory. The AIMAll v14.11.23 Professional package [50] for topological analysis was used for QTAIM calculations [51]. For this purpose, the “Proaim” basin integration approach was employed using “superfine” interatomic surface mesh and “very high” outer angular quadrature. The atomic (integrated) Laplacian values allowed for monitoring the accuracy of basin integrations. Furthermore, the Poincarè-Hopf rule was satisfied for all carried out calculations. The *ρ*, ∇^2^*_ρ_* parameters at the bond critical points (BCPs) concerning dipole polarizability tensors were calculated based on the optimized structures (taking into consideration the already calculated interaction energy). With the use of the generated wavefunction (*.*wfn*) files Reduced Density Gradient (RDG) [52] as well as Interaction Region Indicator (IRI = [*ρ*(*r*)]*^a^*/(|∇*ρ*|) + *b*); where *a* and *b* correspond to “uservar” and “uservar2” in settings.ini file (Multiwfn 3.7 software; Beijing Kein Research Center for Natural Sciences, Beijing, China) [53,54], respectively; if “uservar” is 0, *a* is set to the recommended value 1.1, while if “uservar2” is 0, then *b* is set to the recommended value 0.0005) parameters (Multiwfn 3.7 software from the Beijing Kein Research Center for Natural Sciences, default settings) were calculated. The computations of the RDG and IRI factors were carried out for ATR-IRB complexes with the corresponding lowest and highest values of the interaction energy (based on the optimized geometries using B3LYP and B97D3 functionals). For free energy of solvation calculations of complexes **V** and **VII**, the Truhlar’s Solvation Model Based on Density (SMD) [55] as well as the M062X/6-31G(d,p) approach were used.

### 2.8. Intrinsic Dissolution Testing

The intrinsic dissolution rate (IDR) of the crystalline, amorphous, and co-amorphous (1:1 molar ratio) ATR and IRB samples was measured by the rotating disc method [56] using the Wood’s apparatus in an Erweka DT60 dissolution testing station (ERWEKA GmbH, Langen, Hessen, Germany). A 150 mg of sample was compressed at 1300 psi and held for 40 s in a die to form an 8 mm diameter disc. Dissolution studies were performed in 500 mL of 2% *w*/*v* sodium dodecyl sulphate (SDS) in ultra-pure water maintained at a temperature of 37 ± 0.5 °C and using the disk rotation speed of 100 rpm. Dissolution studies were carried out for 120 min and the concentration of API was determined online every 5 min by a UV-Vis spectrophotometer Nicolet Evolution 300 (Thermo Electron Corporation, Waltham, MA, USA) at 228 (IRB signal), 244 (ATR signal), and 344 nm (background correction) based on the dual-wavelength analysis method [57,58]. All measurements were performed in triplicate. IDR, the rate of mass transfer from solid to liquid state when conditions such as surface area, pH, ionic strength, and stirring speed are kept constant, was determined using the following equation [59]:

(3) $$\mathrm{IDR}=\frac{C}{t}\frac{V}{S}\;=kC_{s}\;$$

where *C* is the drug concentration at time *t*, *V* is the volume of the test solution, *S* is the surface area of the disc, *k* is intrinsic dissolution rate constant, and *C*_s_ is the saturation solubility of the drug. The IDR was calculated from the slope of each curve for a time period of 0–120 min.

## 3. Results and Discussion

### 3.1. Thermal Analysis

The identification of possible interactions between APIs in the fixed-dose combination is of paramount importance at an early stage of the formulation development process [1]. Thermal analysis is frequently used to study the drug-drug and drug-excipient compatibility [60,61,62].

TGA curves (Figure S1, Supplementary Materials) of the individual drugs and the 50:50 (*w*/*w*) ATR-IRB physical mixture indicate that no degradation due to the interaction between the drugs takes place and that the ingredients change individually in the mixture, thereby suggesting no chemical incompatibility.

**Figure 2:**
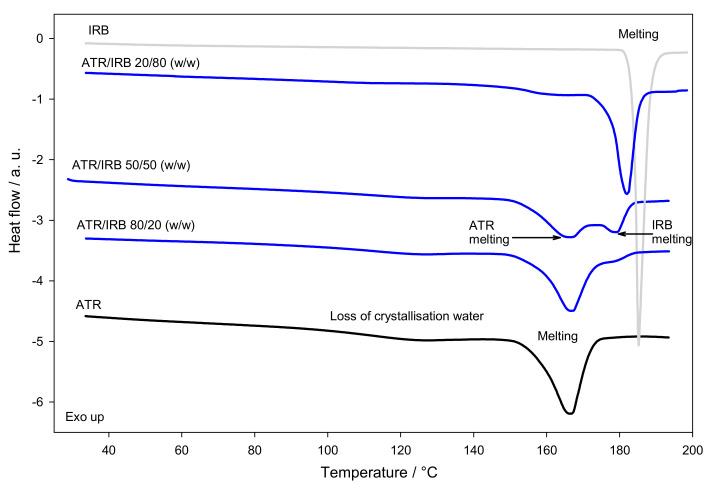
Standard DSC curves of crystalline atorvastatin (ATR), crystalline irbesartan (IRB) and their 80/20, 50/50 and 20/80 (*w*/*w*) physical mixtures. All runs obtained at a heating rate of 10 °C min^−1^.

Figure 2 shows the standard DSC traces of crystalline ATR, IRB, and the physical mixtures in different concentrations of the investigated materials from 25 to 190 °C at a 10 °C min^−1^ heating rate. The standard DSC curve of ATR shows two endothermic events, one very broad peak with a maximum at around 113.4 °C corresponding to the loss of crystallization water as ATR is a trihydrate (see TGA, Figure S1, Supplementary Materials). The second event with onset at 150.2 ± 0.9 °C (Δ*h*^ATR^ = 81 ± 3 J g^−1^) is ascribed to melting. The results were similar to the previously reported, i.e., melting with an onset in the range 141–148 °C and enthalpy of fusion ranged from 81.4–87.1 J g^−1^ [6].

The standard DSC curve of IRB shows one sharp endothermic peak with an onset at 183.8 ± 0.2 °C and an enthalpy of fusion Δ*h*^IRB^ = 94.1 ± 0.5 J g^−1^ due to melting. The results suggest that the investigated IRB is a polymorphic form A [63,64].

To further investigate the compatibility of the drugs, 80/20, 50/50, and 20/80 (*w*/*w*) physical mixtures (PMs) of ATR/IRB were investigated. DSC provides information about possible interactions between APIs. These can be assessed depending on the peak appearance, its shift on the temperature scale, disappearance of phase transition peaks and/or differences in the enthalpy values in thermal curves of the components in physical mixtures. The DSC trace of the 80/20 ATR/IRB mixture showed that the melting peak of ATR broadened and shifted to a higher temperature (158.0 ± 0.7 °C) and slightly overlapped with the IRB melting peak. The DSC curve of the ATR/IRB 20/80 physical mixture showed a small endotherm arising from melting of ATR with an onset at 151 ± 1°C and the second endotherm arising from melting of IRB, shifted into the lower temperature (177.9 ± 0.6 °C). In the thermogram of the 50/50 physical mixture ATR/IRB two endothermic events arising from ATR and IRB melting were observed. The clear appearance of both melting peaks and no other events suggest that no chemical interaction takes place in the mixture. However, in all investigated PMs, changes in the melting onset temperature and endotherm peaks of both APIs do occur (Table 1). This may be due to the mixing of components, which results in a decrease in the purity of each component in the mixture, showing as a broadening of the peak. It is expected that the enthalpy and temperature of fusion will change. The effect may also be due to physical interactions present between the components [65,66,67]. For further investigation of the material’s compatibility, FT-IR was applied.

**Figure 3:**
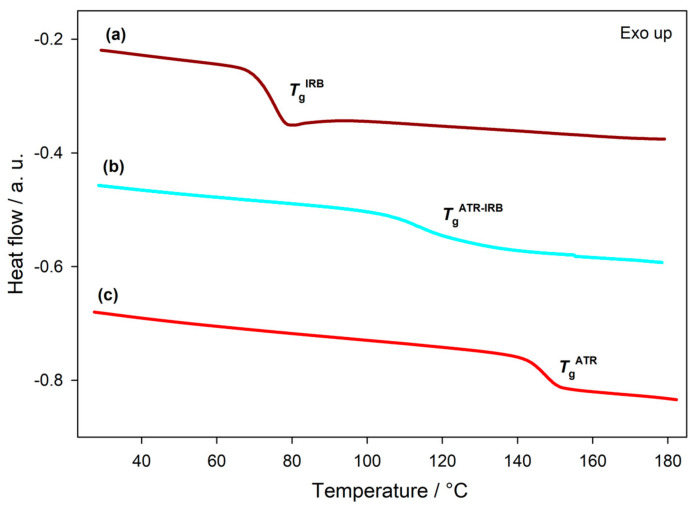
Second heating standard DSC curves of atorvastatin (ATR), irbesartan (IRB) and the 1:1 mol/mol atorvastatin-irbesartan (ATR-IRB) system. The curves show the glass transition phenomenon confirming amorphization of the APIs; a single glass transition for the mix indicates mixing of the material at the molecular level and co-amorphization of the system. All runs were obtained at a heating rate of 10 °C min^−1^.

Figure 3 shows the second heating DSC curves for individual APIs and the quench cooled melt of the ATR-IRB 1:1 mol/mol mixture. The glass transition was detected in all systems, whereas the melting endotherm was not observed, indicating the amorphous nature of all systems. For the individual amorphous APIs, *T*_g_ values of 145.6 ± 0.9 and 75.7 ± 0.2 °C with the change of heat capacity at *T*_g_, Δ*c_p_* of 0.39 ± 0.04 and 0.50 ± 0.01 J g^−1^ K^−1^ were observed for ATR and IRB, respectively, similar to the values stated in the previous reports [6,64]. On the analysis of the quench cooled melt melted ATR-IRB 1:1 mol/mol mixture, a single *T*_g_ at 118 ± 2 °C with the change of heat capacity of 0.34 ± 0.01 J g^−1^ K^−1^ was detected, showing that the system is entirely miscible at the molecular level [68].

The glass transition temperature is an important physical parameter of amorphous drugs, as it indicates a boundary between phases of low and high molecular mobility. Therefore, the glass transition temperature may infer the storage conditions of amorphous APIs [11]. The molecules of amorphous API may re-order below the *T*_g_; this process of ordering strongly depends on the storage temperature [10,69]. In the case of the co-amorphous ATR-IRB system, the *T*_g_ has increased in comparison to pure IRB (anti-plasticization effect), thus, an increased physical stability is expected [26]. Furthermore, a negative deviation from the *T*_g_ predicted using the CK equation is observed (Table 2). This behavior is an indication of non-ideal mixing behavior and a possibility of intermolecular interactions occurring between the APIs [70]. The process of intermolecular reorganizing leading to interactions probably occurs during the heating of the crystalline drugs in the PM as deviations in *T*_m_ and Δ*h* of melting in comparison to pure APIs were noted (Table 1).

The disordered structure of an amorphous material stored below its *T*_g_ becomes more structurally ordered over time [71], and both total enthalpy and volume are reduced toward the equilibrium state. This phenomenon is called physical aging [72]. The parameter characterizing the process of physical aging is the enthalpy of relaxation, also known as recovery enthalpy [72]. It is known that aged amorphous pharmaceuticals can have different physicochemical properties [37,38,39], and this may depend on the local order of the amorphous structure [73]. Furthermore, it is established that molecular mobility still exists below *T*_g_ and is reflected in Johar–Goldstein (JG) or beta relaxations [74]. This molecular mobility may trigger the process of recrystallization. To investigate the possibility of beta relaxation kinetically activating the process of recrystallization at sub-*T*_g_ temperatures and a physical aging process, short-term stability studies were performed.

**Figure 4:**
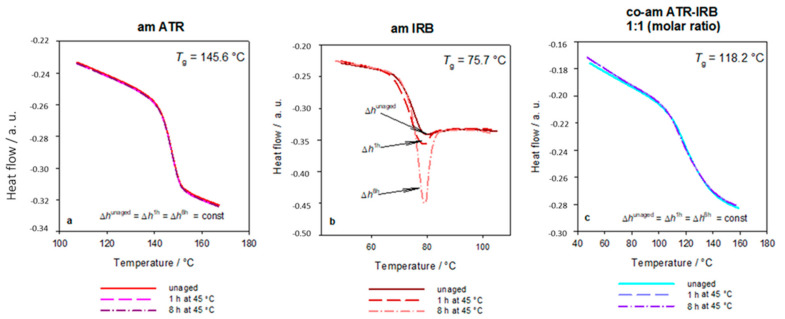
Standard DSC traces of (**a**) amorphous atorvastatin (ATR), (**b**) amorphous irbesartan (IRB), and (**c**) co-amorphous atorvastatin-irbesartan (ATR-IRB) unaged and aged at 45 °C for 1 and 8 h. The enthalpy relaxation (Δ*h*) was not changed after aging in the case of ATR and co-am ATR-IRB, whereas a significant change is observed after 1 and 8 h of aging for IRB. All runs were obtained at a heating rate of 10 °C min^−1^.

Figure 4 shows the effect of physical aging of pure amorphous APIs and co-amorphous ATR-IRB system. The aging process was assessed by DSC, and the thermograms showed that the enthalpy relaxation overlapped with the change of heat capacity in the glass transition region [75,76]; the difference between an unaged and aged sample is visibly presented in Figure 4b. In general, following the *T*_g_ − 50 rule, i.e., the storage at a temperature below 50 °C of *T*_g_ may arrest JG relaxations and prevents the recrystallization of an amorphous drug [10,77], but a physical aging process may still occur at that temperature. As IRB can undergo a physical aging process at 25 °C [36], the samples were aged at a higher temperature, i.e., 45 °C, to stimulate a possible aging process in ATR and co-amorphous ATR-IRB. Amorphous ATR, due to its high *T*_g_, did not undergo physical aging. The enthalpy relaxation (Δ*h*) of amorphous IRB has changed from 0.8 J g^−1^ after 1 h to 3.3 J g^−1^ after 8 h of aging. It can be noted that IRB exhibits aging during the cooling-reheating process, i.e., enthalpy relaxation can be observed for sample reheated immediately after the cooling. This was confirmed by Temperature-Modulated DSC [40] (Figure S2, Supplementary Materials). In the case of the co-amorphous ATR-IRB, physical aging is not observed after 8 h, most likely indicating slower molecular motions in the co-amorphous system, which might signify increased physical stability despite the presence of IRB in the system [10,69]. Neither a recrystallization exotherm nor a melting endotherm was observed for all aged samples confirming the amorphous nature of all materials after the aging process.

### 3.2. FT-IR Spectroscopy

**Figure 5:**
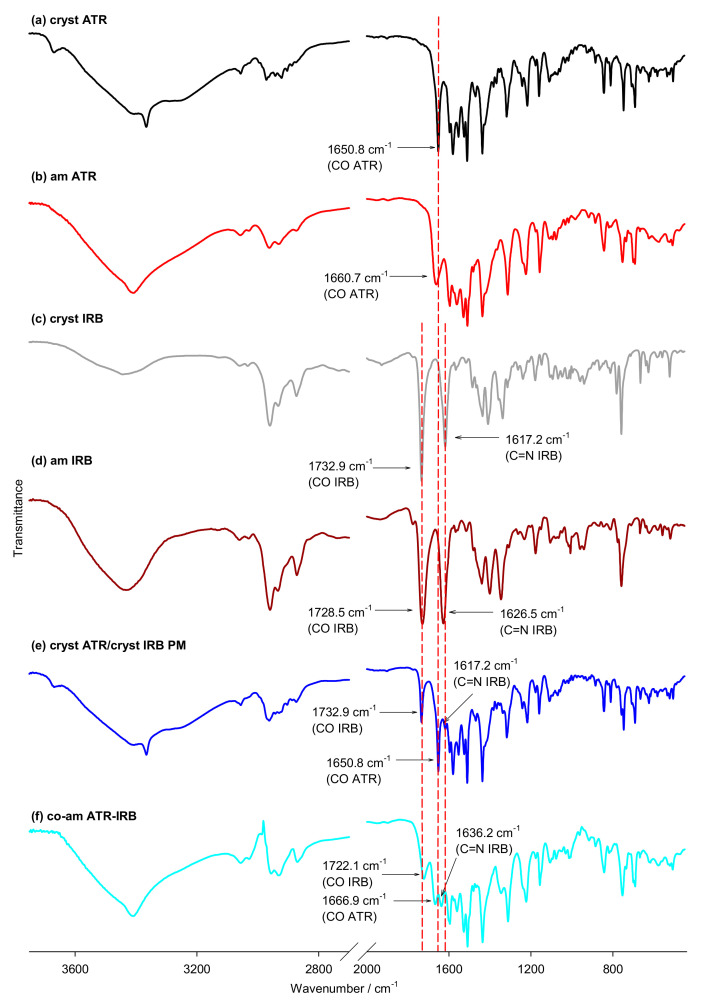
FT-IR spectra of (**a**) crystalline ATR, (**b**) amorphous ATR, (**c**) crystalline IRB, (**d**) amorphous IRB, (**e**) the physical mixture (PM) of crystalline ATR and IRB (1:1 mol/mol), and (**f**) co-amorphous ATR-IRB.

FT-IR spectroscopy was used to evaluate interactions between the ATR-IRB system at the molecular level (Figure 5). The measurements revealed clear differences between the crystalline and amorphous ATR and IRB samples. The spectra of amorphous ATR and IBR, in comparison to the crystalline materials, showed peak broadening and band displacements. The carbonyl groups in ATR participated in H-bond formation in the crystalline material [78]. Furthermore, Brus et al. found that the amide carbonyl molecular site is associated with the conformational changes occurring predominantly in the amorphous state [79]. Therefore, the carbonyl group of ATR was selected for a detailed analysis. There is a large difference between the carbonyl stretching vibration of crystalline ATR (1650.8 cm^−^^1^) and the amorphous form of ATR (1660.7 cm^−^^1^); similar results were previously published by Kim et al. [3]. Furthermore, there is a shift of the ATR carbonyl group in the co-amorphous system (1666.9 cm^−^^1^). This shift is likely to be arising from the interaction of the carbonyl group of ATR and an interacting moiety of IRB via hydrogen bonding.

Also, a clear difference between crystalline and amorphous IRB was detected. Two signals were investigated, one arising from the carbonyl group, which might participate in H-bonds, and the other was the C=N bond in the diazaspiro ring, which is known to be involved in an H-bond in the polymorph form B [42,80]. Crystalline IRB shows absorption bands at 1732.9 and 1617.2 cm^−1^, assigned to the carbonyl moiety of the carboxyl group and the C=N bond in the diazaspiro ring vibrations, respectively. These values are similar to those reported by Araya-Sibaya et al. (1730 and 1615 cm^−1^, respectively) [63] and Franca et al. [81] (1731.82 and 1616.11 cm^−1^, respectively), confirming the polymorph A structure of the investigated crystalline IRB. These bands change in the amorphous form, i.e., move to 1728.5 cm^−1^ for the –C=O vibration, which is slightly different than that reported previously by Cruz-Angeles et al. (1723 cm^−1^) [34] and Chawla and Bansal (1726 cm^−1^) [36], however, the position of the C=N group, at 1626.5 cm^−1^, is similar to that reported by the latter authors (1626 cm^−1^) [36]. In the co-amorphous system band of the carbonyl group of IRB shifts to 1722.1 cm^−1,^ and the vibration of the C=N bond moves to 1636.2 cm^−1^. Again, this suggests an interaction between ATR and IRB that is most likely a hydrogen bond and present after co-amorphization. The IR spectrum of the equivalent physical mixture of the crystalline materials did not show any significant band shifts with respect to the IR spectra of the components. This spectrum is a superposition of spectra of the original crystalline materials. These results suggest that no interaction takes place in the physical mixture of crystalline materials and confirms the physical and chemical compatibility of both APIs. The DSC results indicate that there may be an interaction between crystalline APIs, however, it has to be noted that the FT-IR measurements were performed at room temperature and any interaction indicated by DSC may have been caused by the high temperature applied during the DSC experiment. The clear molecular interaction between APIs in the co-amorphous system, as suggested by DSC, was confirmed by FT-IR (single glass transition and deviation from Couchman–Karasz equation predicted *T*_g_).

### 3.3. Computational Investigations

The objective of computational investigations was to improve our understanding of possible interactions present between ATR and IRB. To the best of our knowledge, such investigations are the first attempt to describe, quantifiably, intermolecular interactions between ATR and IRB in their possible complexes from the standpoint of quantum chemistry. Four possible geometries of the ATR-IRB complex were proposed. Their initial geometries were prepared considering the mutual position of the carbonyl group of IRB (its 2*H* tetrazole tautomer) and the fluorine, carbonyl, and hydroxyl functionalities within the structure of ATR (complexed with calcium ions). The calculations showed that the absolute value of the total energy difference between the isolated 1*H* and 2*H* tautomer of IRB (estimated at the B3LYP/6-311++G(d,p) or B97D3/6-311++G(d,p) level of theory) were 0.001864 and 0.003134 Hartree, respectively (1.17 and 1.79 kcal mol^−1^, respectively) and implied that the 1*H* tautomer of IRB is energetically slightly more favored. However, the interaction energy in the dimers made of 1*H* and 2*H* tautomers of IRB calculated at the B3LYP/6-311++G(d,p) level of theory was −18.05 (for the dimer of 1*H* tautomers), and −43.78 kcal mol^−1^ (for the dimer of 2*H* tautomers), or when B97D3/6-311++G(d,p) level of theory was used the energy was −44.26 kcal mol^−1^ (for the dimer of 2*H* tautomers); Figures S3–S6 in the Supplementary Materials. Optimization of the 1*H* tautomer of IRB using the B97D3 functional led to cleavage of the tetrazole ring in the dimer. Our calculations provided evidence that the 2*H* tautomer of IRB can form, energetically, more stable complexes. Thus, subsequent calculations considered only the 2*H* tautomer of IRB.

**Figure 6:**
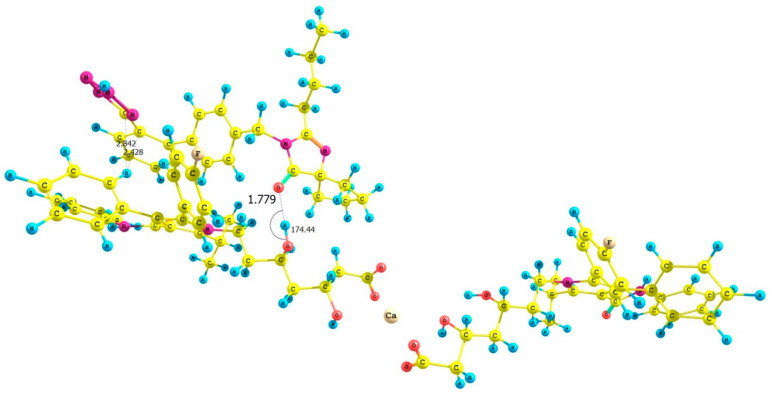
Structure of the optimized ATR-IRB complex **III** (B3LYP/6-311++G(d,p)//B3LYP/6-31G(d,p) level of theory).

**Figure 7:**
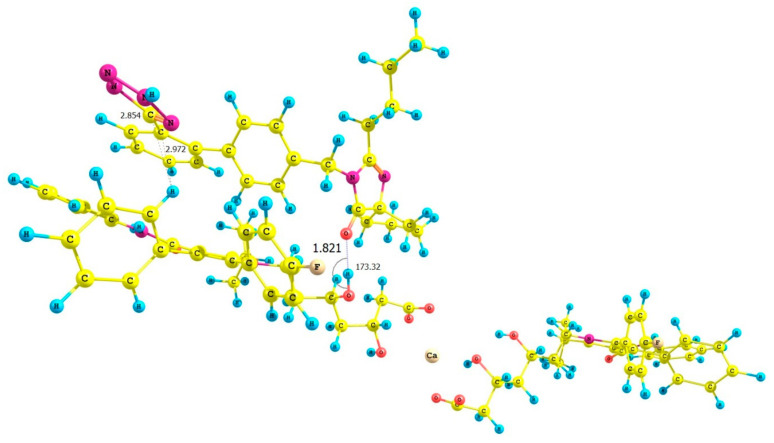
Structure of the optimized ATR-IRB complex **VII** (B97D3/6-311++G(d,p)//B97D3/6-31G(d,p) level of theory).

Next, the complexes at the B3LYP/6-31G(d,p) and B97D3/6-31G(d,p) level of theory were optimized incorporating the Grimme’s D3BJ dispersion model (Cartesian coordinates are given in the Supplementary Materials, Figures S7–S12). The data concerning interaction energies (gaseous phase, counterpoise corrected method, including the basis-set superposition BSSE error) for complexes **I**–**VIII** are shown in Table 3. The lowest interaction energies, with the most negative values, were determined for complex **III** (optimized by B3LYP/6-31G(d,p)), Figure 6, and **VII** (optimized by B97D3/6-31G(d,p)), Figure 7, and were: −34.44 (complex **III**) and −33.20 (complex **VII**) kcal mol^−1^, respectively. These interactions between the hydroxyl group of ATR (as a hydrogen bond (HB) donor) and the carbonyl moiety of IRB (as a HB acceptor) were due to similar geometries of complexes **III** and **VII**, in which strong hydrogen contact O−H⋯O=C was detected (Figure 6 and Figure 7). The distance between the proton of the hydroxyl group and the carbonyl functionality (*d*) was 1.779 or 1.821 Å for complex **III** and **VII**, respectively, with the corresponding angle (*θ*) of 174.44 or 173.32°, respectively. Furthermore, it was noticed that in these cases, the proximity of the phenyl ring of ATR and the tetrazole system of IRB (ca. 2.4–2.8 Å) had an influence on the interaction strength resulting in possible van der Waals (vdW) type contacts. It is noteworthy that the discussed O−H⋯O=C type of contacts seemed to be crucial for intermolecular interactions in ATR-IRB complexes compared to other interactions. The contact between the fluorine atom of ATR and the carbonyl group of IRB was significantly weaker (*d* > 3.0 Å, complexes **I** or **V**, Figures S7 and S10, respectively, Supplementary Materials). Moreover, the N−H⋯O=C contact (*d* > 1.927 or 2.340 Å, complexes **IV** or **VIII**, Figures S9 and S12, respectively, Supplementary Materials) or when the tetrazolic system of IRB was located in a far distance from ATR’s phenyl ring (complexes **II** or **VI**, Figures S8 and S11, respectively, Supplementary Material) emerged to be weaker too.

**Figure 8:**
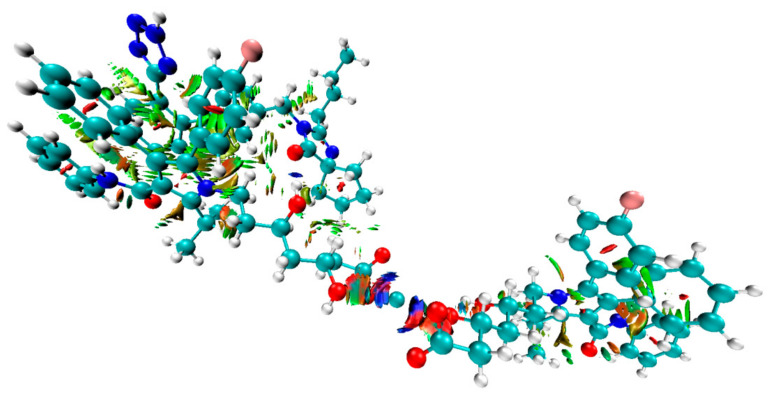
Colored contour surface of reduced density gradient (RDG) highlighting weak and strong interactions in the ATR-IRB complex **III** (B3LYP/6-311++G(d,p)//B3LYP/6-31G(d,p) level of theory; (blue) hydrogen bonds, (green) vdW interactions).

**Figure 9:**
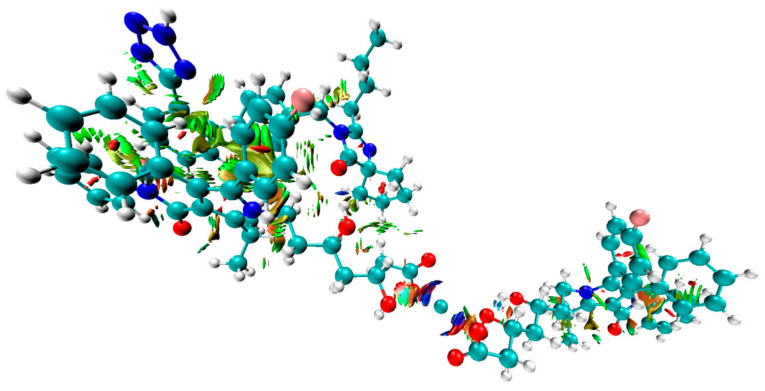
Colored contour surface of reduced density gradient (RDG) highlighting weak and strong interactions in the ATR-IRB complex **VII** (B97D3/6-311++G(d,p)//B97D3/6-31G(d,p) level of theory; (blue) hydrogen bonds, (green) vdW interactions).

QTAIM computations supported the significant role of hydrogen type of contact O−H⋯O=C in the structure of complexes **III** and **VII** (Figure 6 and Figure 7). The application of QTAIM allowed to determine the type and properties of a bond (ionic or covalent), including hydrogen bonding. Only complexes **III** and **VII** were investigated. The positive or negative values of the Laplacian (∇^2^*_ρ_*) at the BCP can determine whether the covalent (∇^2^*_ρ_* < 0) or electrostatic (∇^2^*_ρ_* > 0; ionic or weak hydrogen bonds) or van der Waals type of interactions is present within the closed-shell systems. The values for the electron density topological parameters *ρ* and ∇^2^*_ρ_* were 0.0359 and 0.1246 as well as 0.033 and 0.1120 a. u. for complexes **III** and **VII**, respectively. It was observed that the *ρ* values were small, the ∇^2^*_ρ_* values were positive but relatively small, and the value of the density of electronic kinetic energy was equal to the absolute value of the density of electronic potential energy in the BCP. Thus, they indicate that a strong, O−H⋯O=C type of hydrogen bond with an electrostatic character was present in the complexes. The energy of HBs was estimated according to the refs. [82,83] and was −10.29 and −9.01 kcal mol^−1^ for complexes **III** and **VII**, respectively. On the other hand, in the case of complexes with the less negative value of interaction energy (complexes I and V, Table 3.), the values of *ρ* and ∇^2^*_ρ_* were 0.0055 and 0.0235 as well as 0.0078 and 0.031 a. u., respectively, proving a significantly weaker nature of the F⋯O=C contact between ATR and IRB (the estimated energy of these contacts was −0.92 and −1.35 kcal mol^−1^ for complexes **I** and **V**, respectively). In the case of all analyzed complexes, the absolute value of bond ellipticity (*ε*) indicated the cylindrical, directional nature of contacts. The non-covalent interaction index (NCI) characterizes weak intermolecular (non-covalent) interactions. The NCI parameter is related to the Reduced Density Gradient (RDG). A graphical representation of the contour surface of the RDG considering Grimme’s D3BJ dispersion model as well as its scatter diagram for the analyzed complexes **I** and **III** as well as **V** and **VII** (Table 3.) are given in Figure 8, Figure 9 and Figures S13–S19 (Supplementary Materials). Results showed that the strong hydrogen bond contact present between the hydroxyl group of ATR and the carbonyl moiety of IRB in the complexes had the most negative value of interaction energy (blue color in Figure 8 and Figure 9, complexes **III** and **VII**). Moreover, it was noticed that the intermolecular vdW interaction between the aromatic rings was favorable in these complexes (**III** and **VII**) compared to complexes **I** and **V**, in which the F⋯O=C contact dominated. In the RDG isosurface map (Figure 8 and Figure 9, Figures S14 and S15 of Supplementary Materials), the green and red regions are related to the presence of van der Waals and steric effects, respectively. However, the blue circular intermolecular hydrogen bonding area evidenced that a hydrogen bonding interaction was indeed present in this region. Since there were spikes in the very negative region of sign(*λ*_2_)*ρ* in complexes **III** and **VII** (Figures S17 and S19 in the Supplementary Materials), it was concluded that they suggested attractive intermolecular interactions in comparison with complexes **I** and **V** (Figures S16 and S18, Supplementary Materials). These findings were supported by the strong nature of the O−H⋯O=C contact in complexes **III** and **VII** based on the Interaction Region Indicator (IRI) analysis (Figures S20–S23, Supplementary Material).

Level of Theory—B3LYP/6-311++G(d,p)//B3LYP/6-31Gd,p)

The molecular electrostatic potential (MEP) was determined by the B3LYP/6-311++G(d,p) and B97D3/6-311++G(d,p) approaches for ATR-IRB complexes **III** (Figure 6) and **VII** (Figure 7) with the geometry previously optimized at B3LYP/6-31G(d,p) or B97D3/6-31G(d,p) level of theory in the gaseous phase, respectively (Figures S24 and S25, Supplementary Materials). To the best of our knowledge, computational studies of MEP of the isolated molecules of ATR and IRB have been limited to calculations based on B3LYP/6-31*G level of theory for ATR [84] and IRB [85]. In our studies, comprising the BSSE methodology to the conformational exploration, the results were refined using a basis set augmented with higher-level polarization functions. The results revealed a significant change in charge distribution occurring mostly in the tetrazole ring and the carbonyl group of IRB and the hydroxyl functionalities of ATR in all analyzed complexes. They show strong electrostatic contacts supported by the most negative value of interaction energy. The calcium ion complexing two molecules of ATR was omitted.

In the next step, the theoretical IR spectra of the selected ATR-IRB complexes were calculated. This approach was limited to complexes **III** (Figure 6) and **VII** (Figure 7), with the most negative value of interaction energy (Table 4). The computations of vibrational frequencies were carried out using the same level of theory as those used for the Self-Consistent Force Field (SCF) optimization procedure and Grimme’s D3 empirical (GD3) dispersion model. The calculated C=O vibrations of complexes **III** and **VII** were visible at higher wavenumbers than those for ATR^a^ (B3LYP functional) and ATR^b^ (B97D3 functional), respectively. Similar behavior was observed for the experimental spectra, i.e., ATR in the co-amorphous system vs. amorphous ATR (Table 4). In the case of C=N vibrations of IRB, the vibration in complexes **III** was visible at the lower wavenumber than for IRB^a^ (B3LYP functional), and for complex **VII** is was at a higher wavenumber than for IRB^b^ (B97D3 functional). In relation to the experimental spectra, the C=N vibration of IRB in the co-amorphous system are observed at a higher wavenumber than for amorphous IRB (Table 4). On the other hand, the computed wavenumber related to the carbonyl moiety of IRB (**III** and **VII**) decreased in comparison with the C=O vibration calculated for the isolated molecule of IRB (IRB^a^ and IRB^b^). A similar trend was observed for the experimental spectra, i.e., IBR in the co-amorphous system and amorphous IRB (Table 4). It is noteworthy that the use of the B97D3 functional calculations to compute the theoretical IR spectral values resulted in a good agreement with the experimental data.

### 3.4. Intrinsic Dissolution Testing

**Figure 10:**
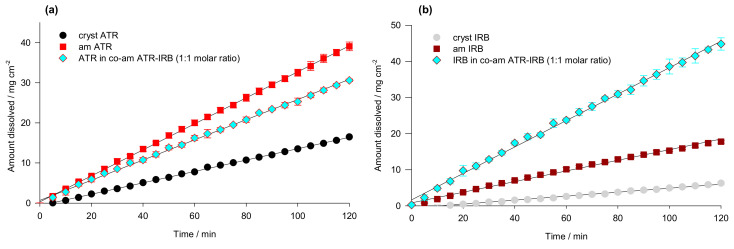
Intrinsic dissolution profiles (*n* = 3) of (**a**) crystalline (cryst ATR), amorphous (am ATR) and co-amorphous atorvastatin (co-am ATR-IRB), and (**b**) crystalline (cryst IRB), amorphous (am IRB), and co-amorphous irbesartan (co-am ATR-IRB) in 2% *w*/*v* aqueous solution of sodium dodecyl sulphate.

The intrinsic dissolution tests were performed to evaluate the impact of co-amorphization on the dissolution behavior on both drugs in 2% *w*/*v* SDS (Figure 10). As expected, for both amorphous APIs, dissolution profiles exhibit a higher dissolution rate than for their crystalline counterparts. On the other hand, the dissolution behavior of both drugs from the co-amorphous system is quite different. The dissolution profile of IRB (Figure 10a) shows about a 2.5-fold, significantly greater (ANOVA, post-hoc HSD test at *α* = 0.05, *p* = 0.0002), intrinsic dissolution rate of API in the co-amorphous system (IDR = 0.365 ± 0.009 mg min^−1^ cm^−2^) in comparison to the pure amorphous drug (IDR = 0.147 ± 0.003 mg min^−1^ cm^−2^). In contrast, ATR in the mixture shows rather an unusual behavior, i.e., the IDR from the co-amorphous system (0.251 ± 0.0004 mg min^−1^ cm^−2^) is lower than the one for amorphous ATR (IDR = 0.324 ± 0.007 mg min^−1^ cm^−2^) (Figure 10b). The co-amorphous materials are generally associated with increased solubility and dissolution rates [16,17]. It is, therefore, surprising to find that ATR from the co-amorphous ATR-IRB material has a lower dissolution rate than pure amorphous ATR. It is worth noticing that the IDRs of amorphous IRB and ATR are 2.67 and 2.28 times faster (statistically significant) than their crystalline counterparts, respectively.

To explain the results of dissolution studies, DFT studies were employed once again. It can be concluded that strong hydrogen bonds were present between the molecules in complexes **III** and **VII**. The cavity between the ATR and IRB subunits of these complexes was smaller in comparison to complexes **I** and **V**. Therefore, due to the stronger HBs and steric hindrance within the structure of complexes **III** and **VII**, the penetration of water molecules into the complex may be regarded negligible, and their dissolution in water seems to be less favorable when compared to complexes **I** and **V**. This was supported by the additional free energy of solvation calculations for complexes **V** and **VII** using the Truhlar’s SMD solvation model and the M062X functional. The estimated energy was −110.70 and −104.54 kcal mol^−1^ for complex **V** and **VII**, respectively, consistent with the lower values of the dissolution rates due to, most likely, lower solubility of complex **VII**.

## 4. Conclusions

The co-amorphous system ATR-IRB was successfully obtained by melting with subsequent cooling of a crystalline physical mixture of APIs. The co-amorphous formulation showed a significant improvement regarding the IDR of IRB over the pure crystalline as well as an amorphous counterpart. An unusual behavior was observed for ATR, as the IDR of ATR from the co-amorphous formulation was slightly lower than the IDR of amorphous ATR on its own. Short-term physical aging studies proved that the ATR-IRB co-amorphous system remains in the amorphous form. Furthermore, no physical aging occurred in the co-amorphous system. FT-IR and analysis of the *T*_g_ value of co-amorphous system using the Couchman-Karasz equation and DFT calculations revealed molecular interactions between the APIs, which may have contributed to the increased physical stability (no physical aging). The computational studies confirmed strong intermolecular interactions between ATR and IRB in the co-amorphous system as found by DSC and FT-IR experiments. The QTAIM and interaction energy computations, as well as calculations considering non-covalent interactions (RDG, IRI) and free energy of solvation supported the supposition of the stronger nature of hydrogen contacts in complexes **III** and **VII**. The behavior of the analyzed ATR-IRB complexes in water was supported by the additional free energy of solvation calculations considering **V** and **VII** complexes suggesting that stronger intermolecular interactions in the latter prevent water molecule ingress into the complex, thus lowering its solubility and translating into lower dissolution rates. Summarizing, co-amorphization enhances both the stability and dissolution rate of irbesartan, whereas it does not have an impact on the short-term stability of atorvastatin but has a negative impact on its dissolution rate.

## Figures and Tables

**Table 1:** Experimental and theoretical fusion parameters for crystalline atorvastatin (ATR), crystalline irbesartan (IRB), and physical mixtures (PMs) 80/20, 50/50, and 80/20 (*w*/*w*) determined by standard DSC.

Sample	*T*_fus_ ± SD/°C		*T*_fus_ $-T_{\mathrm{fus}}^{\mathrm{API}\;\mathrm{mix}}/{}^{\circ}C$		Δ_fus_*h*^exp^ ± SD/J g^−1^			Δ_fus_*h*^theor^/J g^−1^	Δ_fus_*h*^exp^ − Δ_fus_*h*^theor^/J g^−1^
	ATR	IRB	ATR	IRB	ATR	IRB	ATR + IRB	ATR + IRB	
**ATR**	150.2 ± 0.9	N/A	N/A	N/A	81 ± 3	N/A	N/A	N/A	N/A
**IRB**	N/A	183.8 ± 0.2	N/A	N/A	N/A	94.1 ± 0.5	N/A	N/A	N/A
**ATR/IRB 80/20 PM**	158.0 ± 0.7	NPE *	−7.8	NPE *	NPE *	NPE *	72 ± 1	83.7	−11.7
**ATR/IRB 50/50 PM**	153.8 ± 0.6	173 ± 2	−3.6	10.8	NPE *	NPE *	72 ± 5	87.6	−15.6
**ATR/IRB 20/80 PM**	151 ± 1	177.9 ± 0.6	−0.8	5.9	NPE *	NPE *	70 ± 4	91.5	−21.5

* Not possible to estimate.

**Table 2:** Experimental glass transition temperatures (*T*_g_), change in heat capacities (Δ*c_p_*) at the *T*_g_ determined from standard DSC 2nd heating run and calculated *T*_g_s from Couchman–Karasz equation (*T*_g_^CK^) for investigated samples.

Sample	*T*_g_ ± SD/°C	Δ*c*_p_ ± SD/J g^−1^ °C^−1^	*T*_g_^CK^/°C	Δ*T*_g_(*T*_g_ − *T*_g_^CK^)/°C
**ATR**	145.6 ± 0.9	0.39 ± 0.04	N/A	N/A
**IRB**	75.7 ± 0.2	0.50 ± 0.01	N/A	N/A
**Co-am ATR-IRB**	118 ± 2	0.34 ± 0.01	122.9	−4.9

**Table 3:** Interaction energy (Δ*E*) calculated for ATR-IRB complexes at the B3LYP/6 311++G(d,p)//B3LYP/6-31G(d,p) (**I**–**IV**) and B97D3/6-311++G(d,p)//B97D3/6-31G(d,p) level of theory (**V**–**VIII**).

ATR-IRB Complex	Interaction Energy (Δ*E*) Calculated for ATR-IRB Complexes kcal mol^−1^
Level of Theory—B3LYP/6-311++G(d,p)//B3LYP/6-31Gd,p)	
**I**	−1.46
**II**	−25.17
**III**	−34.44
**IV**	−17.00
Level of Theory—B97D3/6-311++G(d,p)//B97D3/6-31Gd,p)	
**V**	−3.24
**VI**	−22.11
**VII**	−33.20
**VIII**	−10.65

**Table 4:** Experimental and theoretical frequencies (IR spectrum) of the selected groups estimated for pure ATR (**ATR^a^** or **ATR^b^**), pure IRB (**IRB^a^** or **IRB^b^**) and ATR-IRB complexes **III** and **VII** at the B3LYP/6-31G(d,p) (**ATR^a^**, **IRB^a^**, and **III**) or B97D3/6-31G(d,p) level of theory (**ATR^b^**, **IRB^b^**, and **VII**). Superscript “a” and “b” indicate calculations using B3LYP or B97D3 functional, respectively.

Group	Wavenumber/cm^−1^										
	Experimental					Calculated					
	cryst ATR	am ATR	am IRB	cryst IRB	co-am ATR-IRB	ATR^a^	ATR^b^	IRB^a^	IRB^b^	III	VII
C=O_ATR_	1650.8	1660.7	N/A	N/A	1666.9	1633.3	1641.7	N/A	N/A	1727.6 *	1649.3 *
C=O_IRB_	N/A	N/A	1732.9	1728.5	1722.1	N/A	N/A	1886.3	1821.4	1765.9	1719.8
C=N_ar(IRB)_	N/A	N/A	1617.2	1626.5	1636.2	N/A	N/A	1745.5	1650.1	1711.3	1651.7

* Average value from two ATR within the complex.

## Data Availability

The data presented in this study are available in the Supplementary Material.

## Supplementary Materials

The following are available online at https://www.mdpi.com/1999-4923/13/1/118/s1, Figure S1: TGA curves for atorvastatin (ATR), irbesartan (IRB) and the 50/50 (*w*/*w*) physical mixture of atorvastatin/irbesartan (ATR/IRB PM). Figure S2: Temperature-modulated DSC curves of amorphous irbesartan (IRB) obtained after cooling the melt at a cooling rate of 10 °C min^−1^. Figures S3 and S4: Structures of the optimized **IRB** 1*H* and 2*H* tautomer dimers (B3LYP functional). Figures S5 and S6: Structures of the optimized **IRB** 1*H* and 2*H* tautomer dimers (B97D3 functional). Figures S7–S12: Structures of the optimized ATR-IRB complexes **I**, **II**, **IV**–**VI**, and **VIII**, Figure S13: Graphical representation of types of interactions regarding the reduced density gradient (RDG) analysis. Figure S14: Colored contour surface of the reduced density gradient (RDG) highlighting weak and strong interaction in the ATR-IRB complex **I** (B3LYP/6-311++G(d,p)//B3LYP/6-31Gd,p) level of theory; blue, hydrogen bonds; green, vdW interactions). Figure S15: Colored contour surface of the reduced density gradient (RDG) highlighting weak and strong interaction in the ATR-IRB complex **V** (B97D3/6-311++G(d,p)//B97D3/6-31Gd,p) level of theory; blue, hydrogen bonds; green, vdW interactions). Figures S16 and S17: Scatter diagrams of the reduced density gradient (RDG) highlighting weak and strong interaction in the ATR-IRB complexes **I** and **III** (B3LYP/6-311++G(d,p)//B3LYP/6-31Gd,p) level of theory; blue, hydrogen bonds; green, vdW interactions). Figures S18 and S19: Scatter diagrams of the reduced density gradient (RDG) highlighting weak and strong interaction in the ATR-IRB complexes **V** and **VII** (B97D3/6-311++G(d,p)//B97D3/6-31Gd,p) level of theory; blue, hydrogen bonds; green, vdW interactions). Figures S20 and S21: Colored contour surface of the interaction region indicator (IRI) highlighting weak and strong interaction in the ATR-IRB complexes **I** and **III** (B3LYP/6-311++G(d,p)//B3LYP/6-31Gd,p) level of theory; green, vdW interactions). Figures S22 and S23: Colored contour surface of the interaction region indicator (IRI) highlighting weak and strong interaction in the ATR-IRB complexes **V** and **VII** (B97D3/6-311++G(d,p)//B97D3/6-31Gd,p) level of theory; green, vdW interactions). Figure S24: Electrostatic potential (ESP) map of ATR-IRB complex **III** calculated in the gaseous phase (B3LYP/6-311++G(d,p)//B3LYP/6-31Gd,p) level of theory; isovalue = 0.002 a.u.). Figure S25: Electrostatic potential (ESP) map of ATR-IRB complex **VII** calculated in the gaseous phase (B97D3/6-311++G(d,p)//B97D3/6-31Gd,p); isovalue = 0.002 a.u.); List of Cartesian coordinates of the optimized IRB, IRB dimers, ATR calcium and ATR-IRB complexes.
