# Supplementary material for: Physicochemical Characterization of a Co-Amorphous Atorvastatin-Irbesartan System with a Potential Application in Fixed-Dose Combination Therapy
Source: Pharmaceutics. 2021 Jan 18;13(1):118. doi: 10.3390/pharmaceutics13010118 (PMC7831949; doi:10.3390/pharmaceutics13010118)
Supplement: Supplementary file 1 [file pharmaceutics-13-00118-s001.pdf]

pharmaceutics

Supplementary Materials: Physicochemical Characterization of a Co-Amorphous Atorvastatin-Irbesartan System with a Potential Application in Fixed-Dose Combination Therapy

Marcin Skotnicki, Barbara Jadach, Agnieszka Skotnicka, Bartłomiej Milanowski, Lidia Tajber, Marek Pyda and Jacek Kujawski

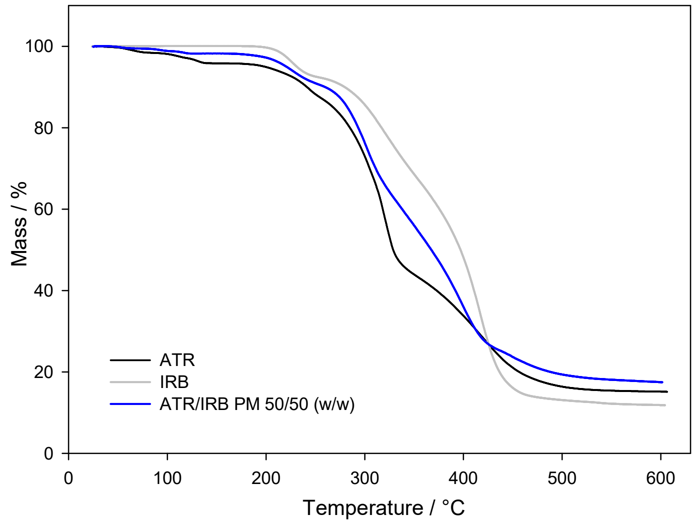

Figure S1. TGA curves for atorvastatin (ATR), irbesartan (IRB) and the 50/50 (w/w) physical mixture of atorvastatin/irbesartan (ATR/IRB PM).

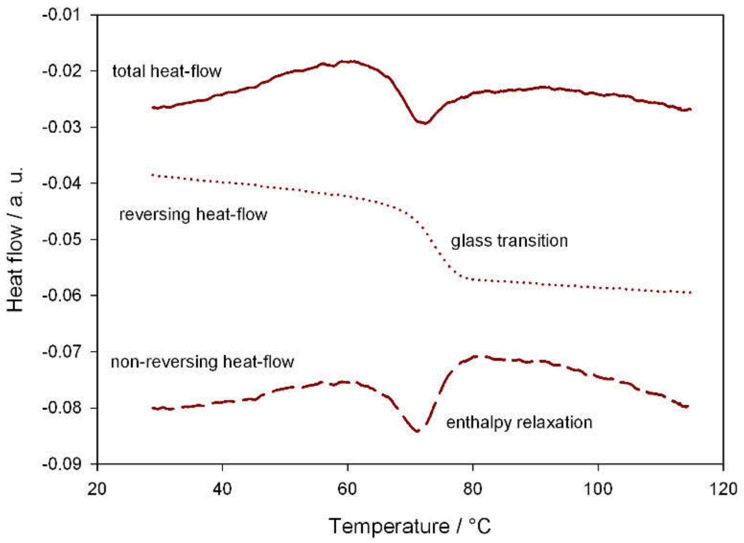

Figure S2. Temperature-modulated DSC curves of amorphous irbesartan (IRB) obtained after cooling the melt at a cooling rate of 10 °C min^{-1}. The total heat flow curve was separated into the non-reversing (relaxation, kinetic) and reversing signal (change in heat capacity at glass transition, thermodynamic). On the non-reversing curve, an endothermic event (enthalpy relaxation) was observed, confirming that IRB underwent non-isothermal physical aging during the cooling–heating cycle. The thermogram was recorded with an underlying heating rate 1 °C min^{-1}, the temperature was modulated at an amplitude of ± 0.5 °C and a period of 60 s.

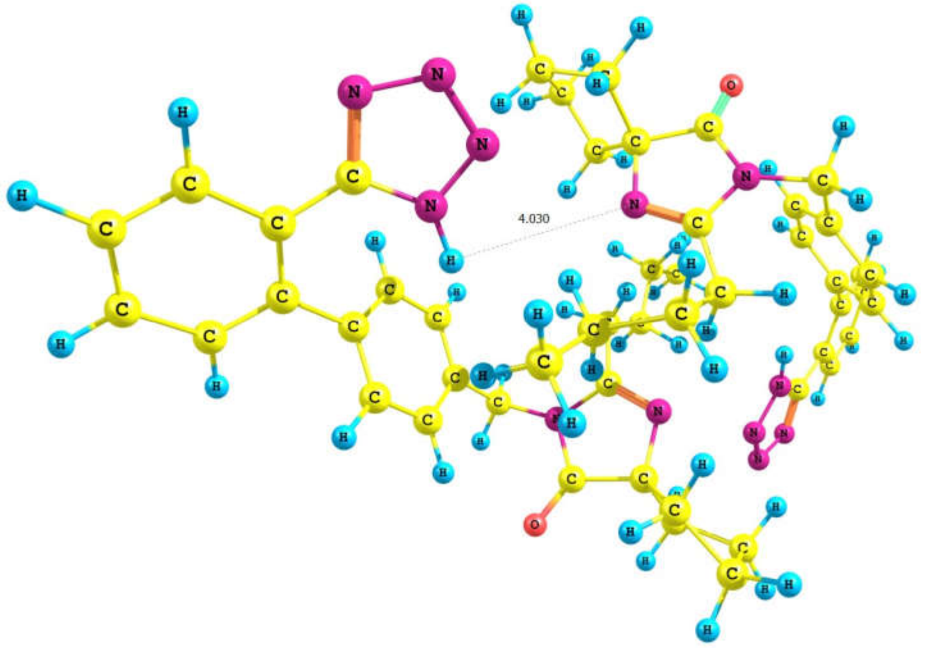

Figure S3. Structure of the optimized **IRB** 1*H* tautomer dimer (B3LYP functional).

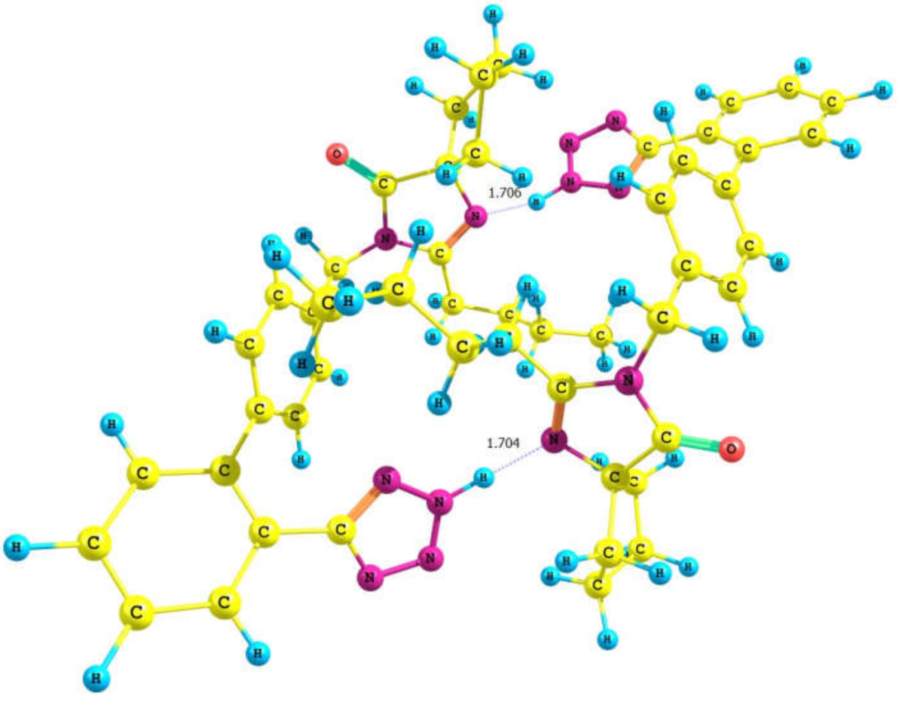

Figure S3. Structure of the optimized **IRB** 2*H* tautomer dimer (B3LYP functional).

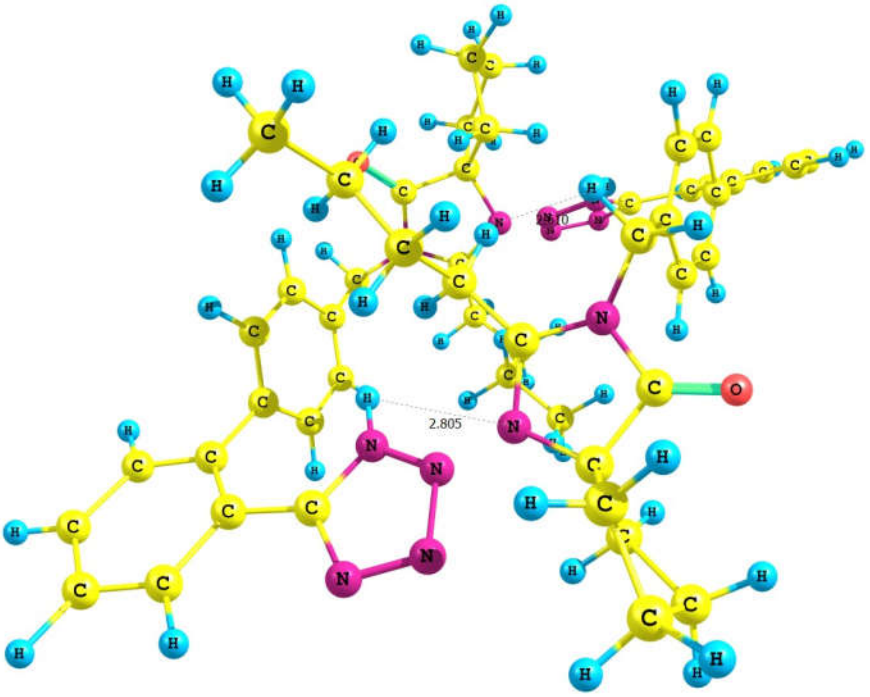

Figure S4. Structure of the optimized **IRB** 1*H* tautomer dimer (B97D3 functional).

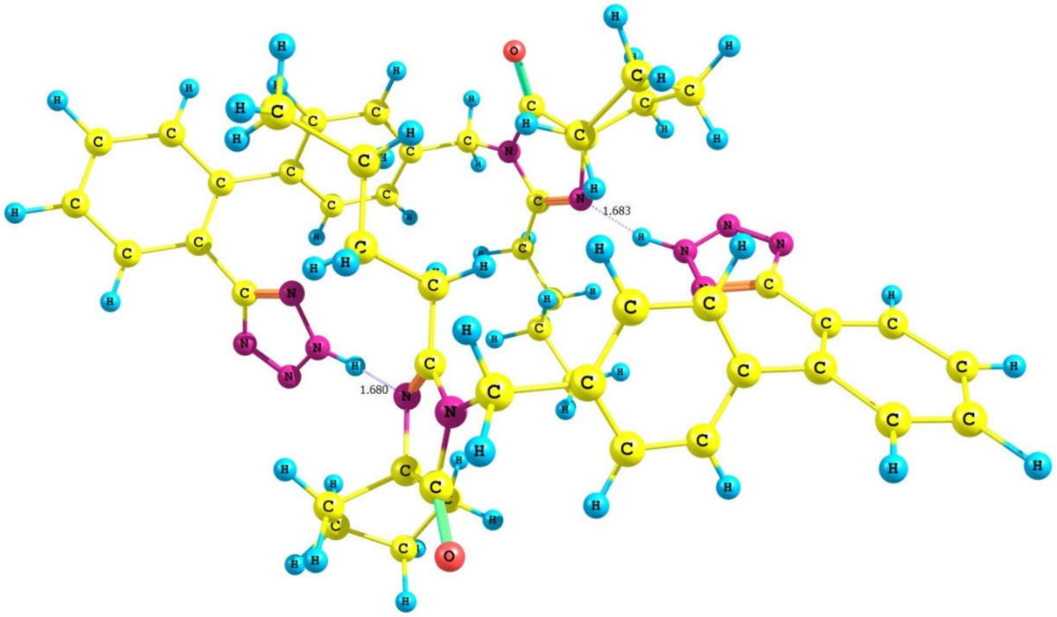

Figure S5. Structure of the optimized **IRB** 2*H* tautomer dimer (B97D3 functional).

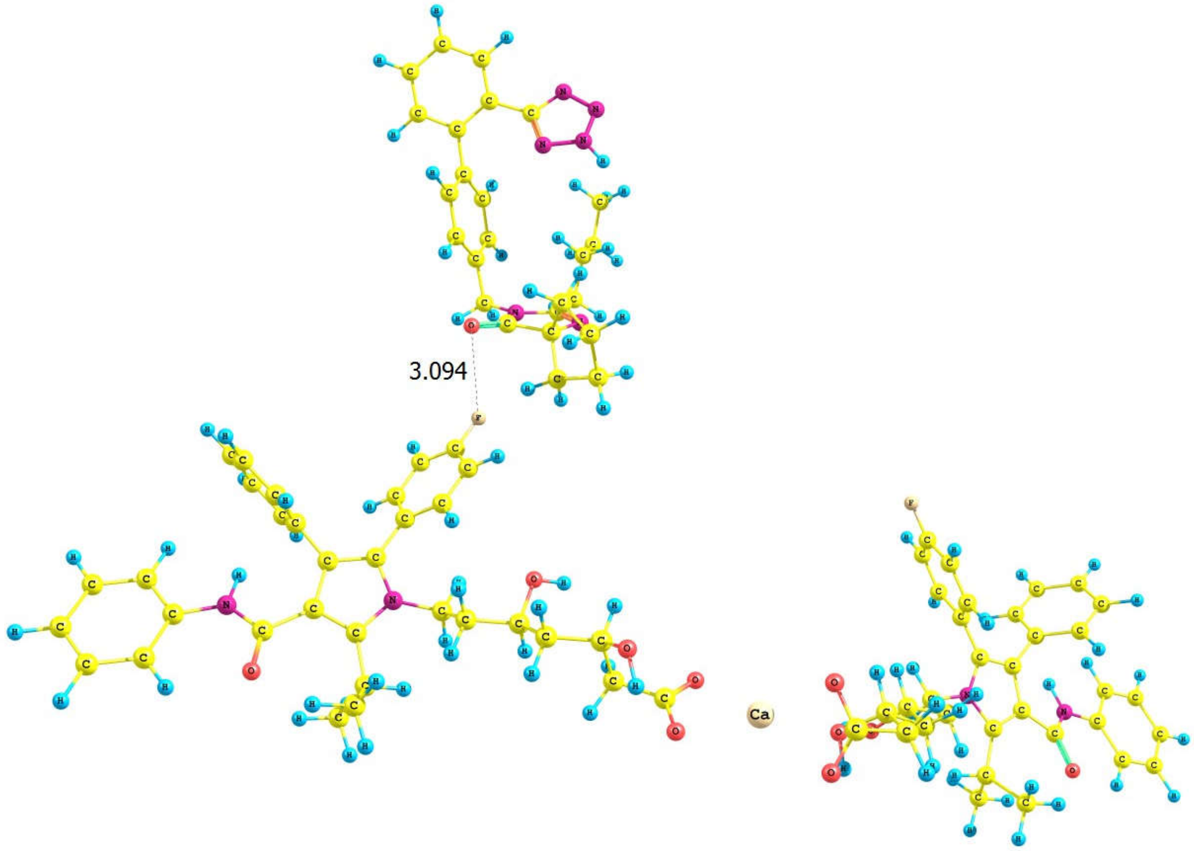

Figure S6. Structure of the optimized ATR-IRB complex **I**.

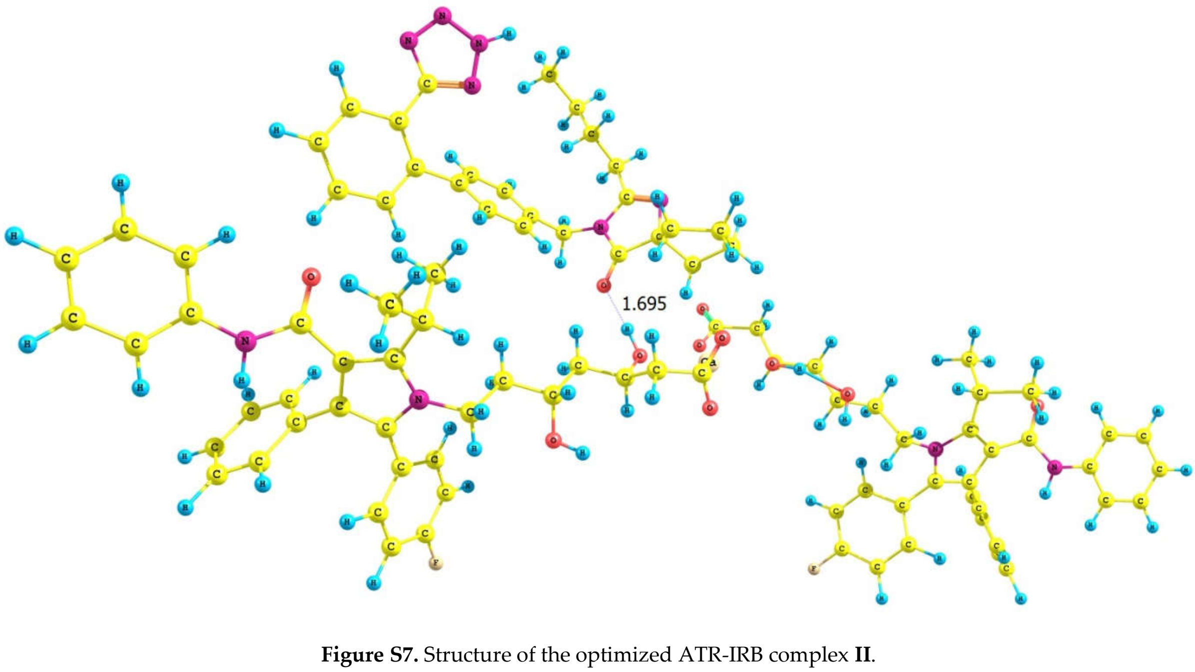

Figure S7. Structure of the optimized ATR-IRB complex **II**.

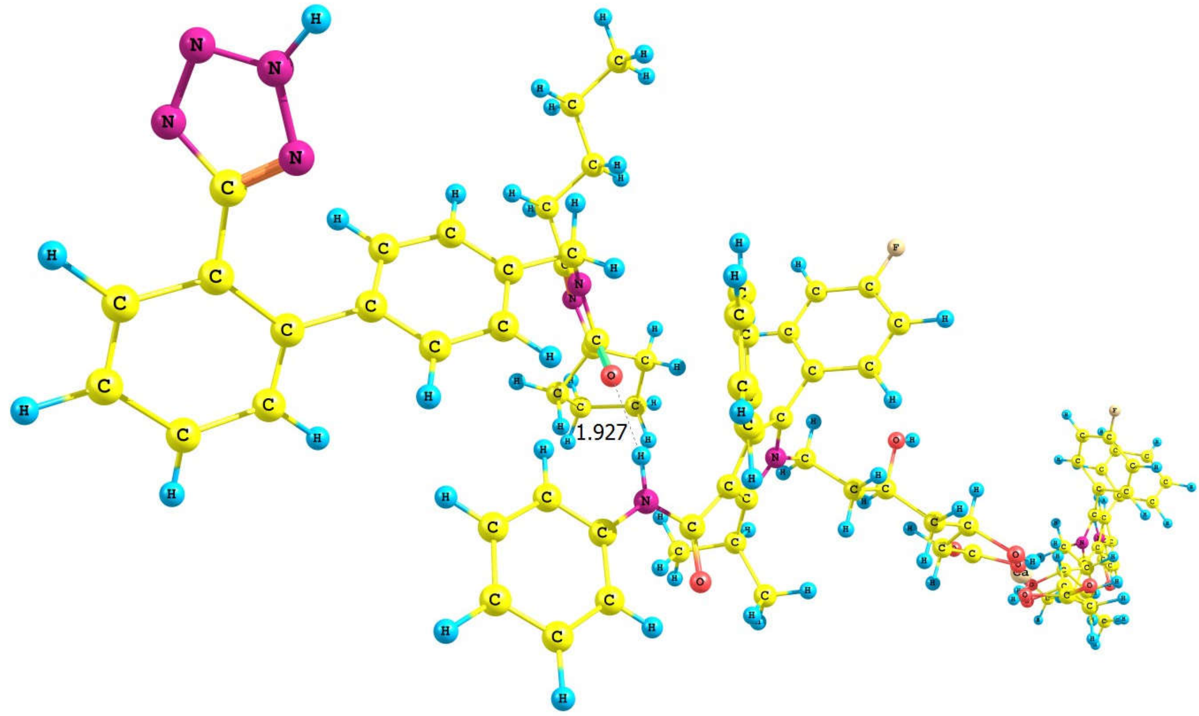

Figure S8. Structure of the optimized ATR-IRB complex **IV**.

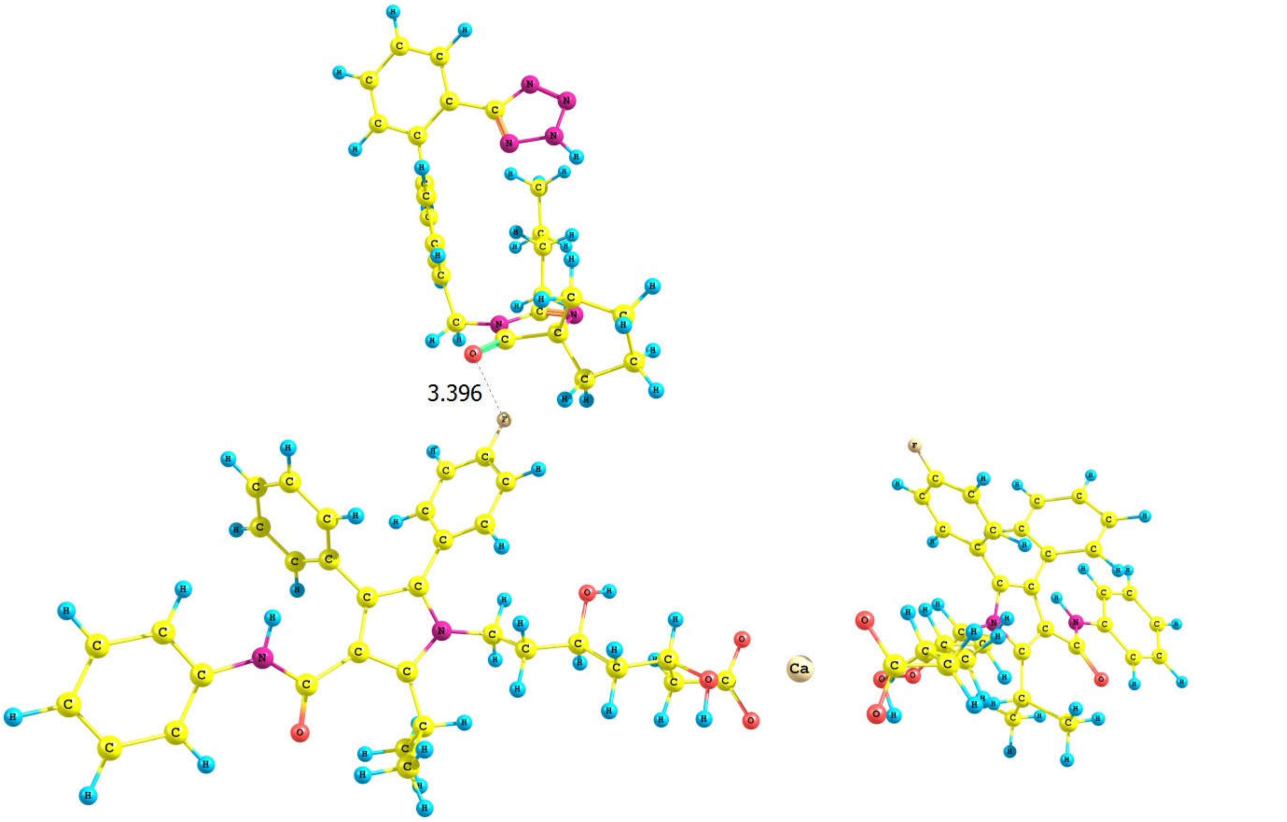

Figure S9. Structure of the optimized ATR-IRB complex **V**.

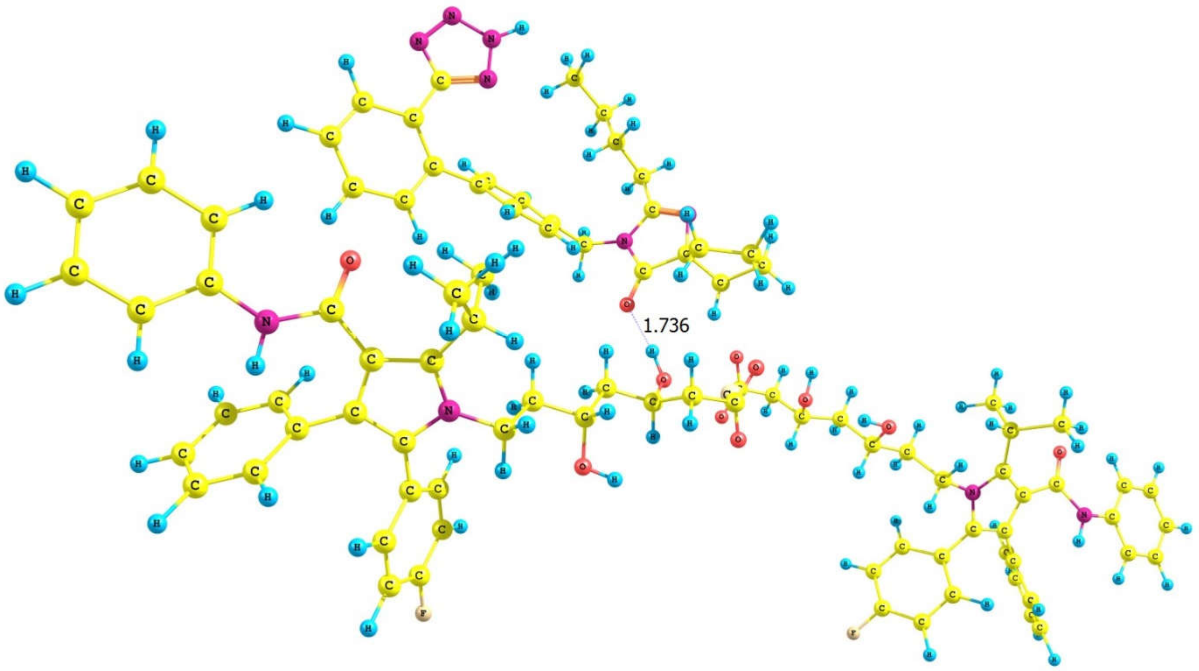

Figure S10. Structure of the optimized ATR-IRB complex **VI**.

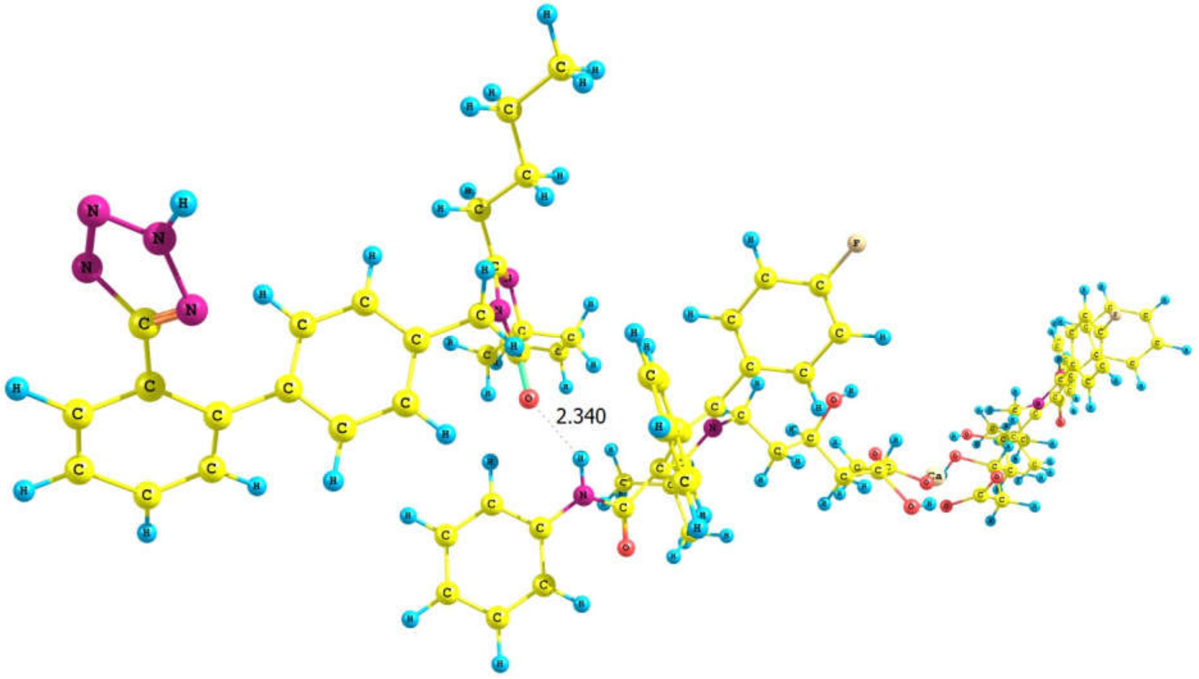

Figure S11. Structure of the optimized ATR-IRB complex **VIII**.

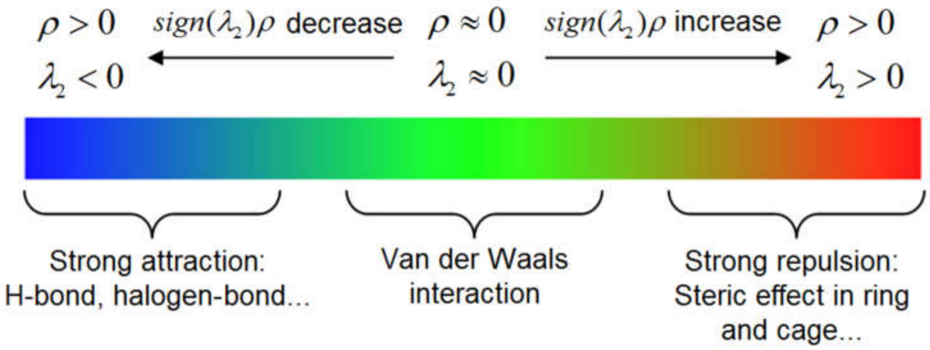

Figure S12. Graphical representation of types of interactions regarding the reduced density gradient (RDG) analysis.

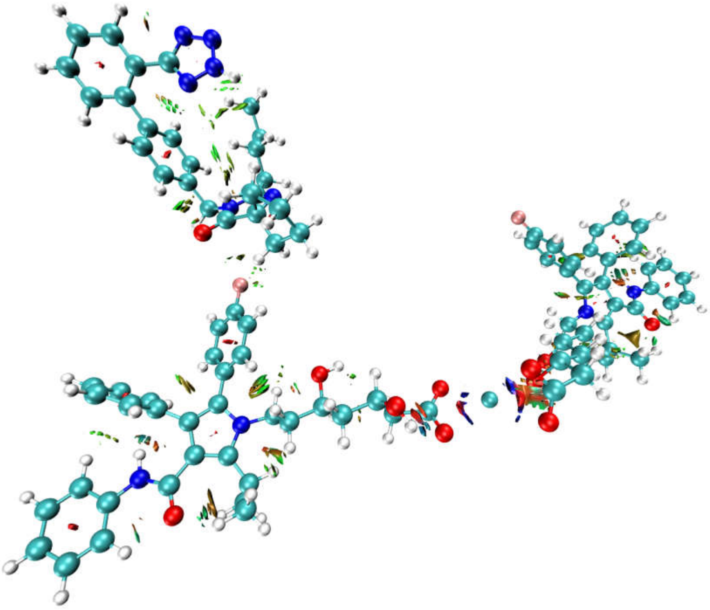

Figure S13. Colored contour surface of the reduced density gradient (RDG) highlighting weak and strong interactions in the ATR-IRB complex **I** (B3LYP/6-311++G(d,p)//B3LYP/6-31Gd,p) level of theory; blue, hydrogen bonds; green, vdW interactions).

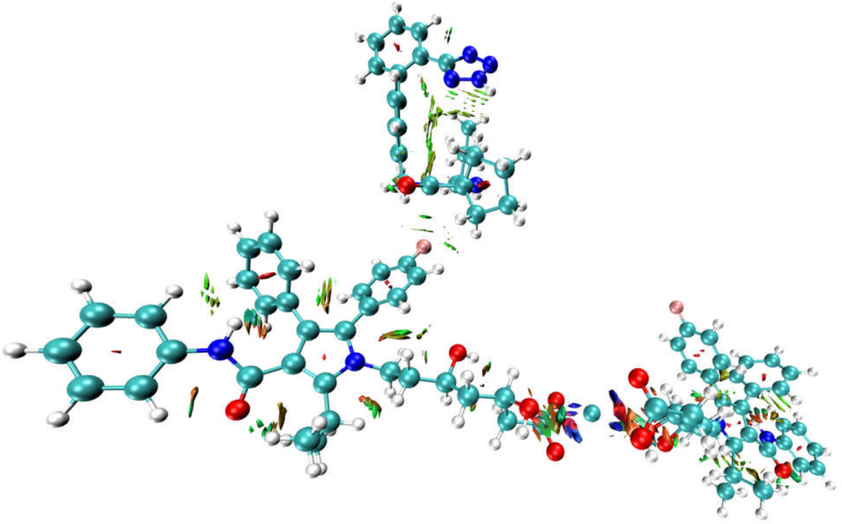

Figure S14. Colored contour surface of the reduced density gradient (RDG) highlighting weak and strong interactions in the ATR-IRB complex **V** (B97D3/6-311++G(d,p)//B97D3/6-31Gd,p) level of theory; blue, hydrogen bonds; green, vdW interactions).

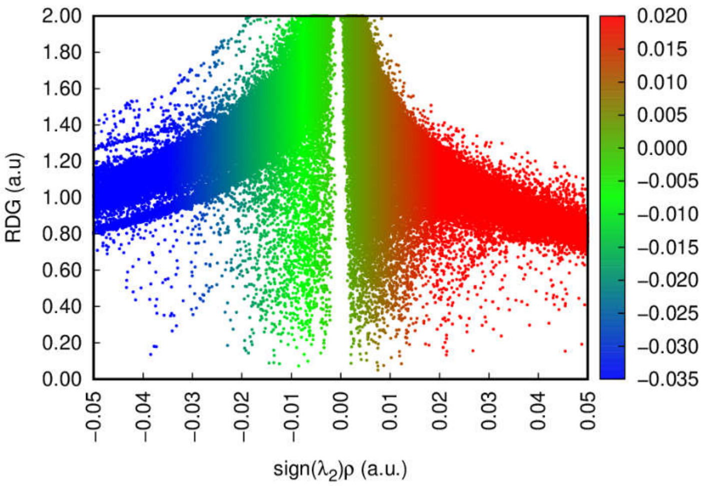

Figure S15. Scatter diagram of the reduced density gradient (RDG) highlighting weak and strong interactions in the ATR-IRB complex **I** (B3LYP/6-311++G(d,p)//B3LYP/6-31Gd,p) level of theory; blue, hydrogen bonds; green, vdW interactions).

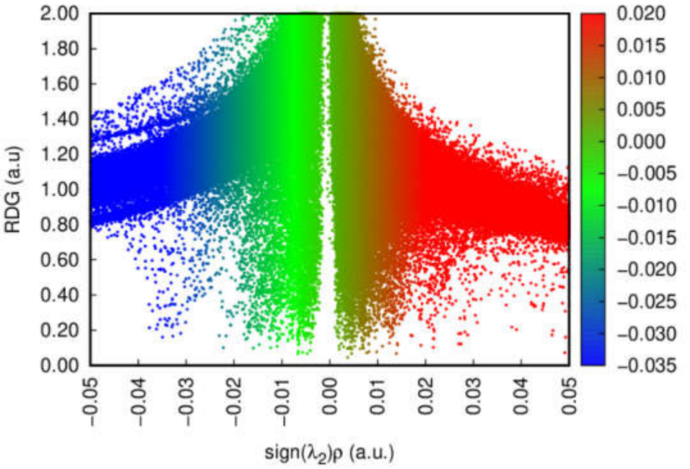

Figure S16. Scatter diagram of the reduced density gradient (RDG) highlighting weak and strong interactions in the ATR-IRB complex **III** (B3LYP/6-311++G(d,p)//B3LYP/6-31Gd,p) level of theory; blue, hydrogen bonds; green, vdW interactions).

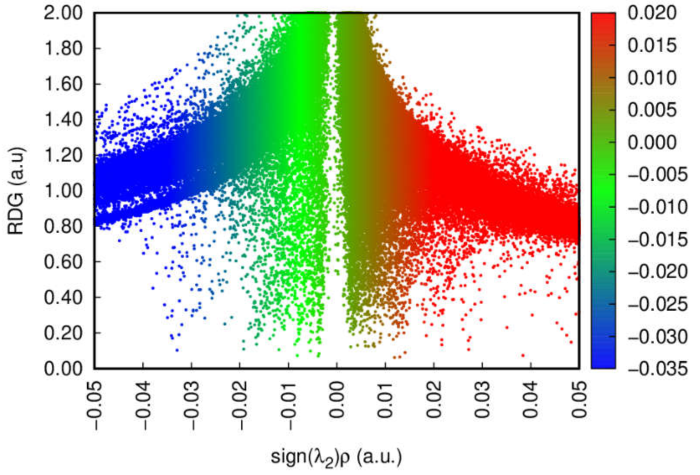

Figure S17. Scatter diagram of the reduced density gradient (RDG) highlighting weak and strong interactions in the ATR-IRB complex **V** (B97D3/6-311++G(d,p)//B97D3/6-31Gd,p) level of theory; blue, hydrogen bonds; green, vdW interactions).

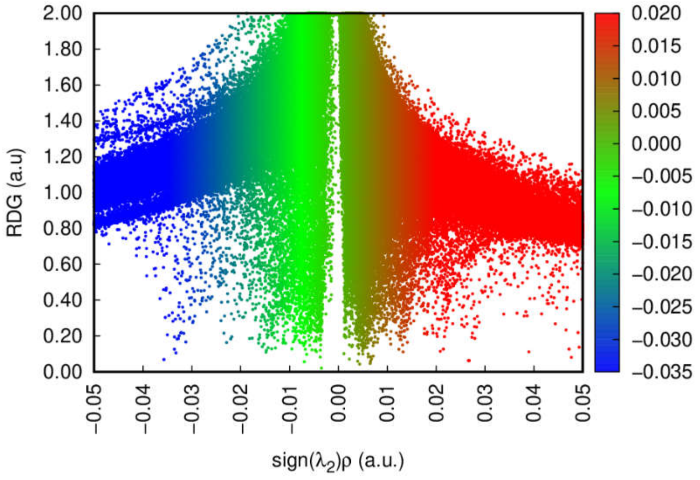

Figure S18. Scatter diagram of the reduced density gradient (RDG) highlighting weak and strong interactions in the ATR-IRB complex **VII** (B97D3/6-311++G(d,p)//B97D3/6-31Gd,p) level of theory; blue, hydrogen bonds; green, vdW interactions).

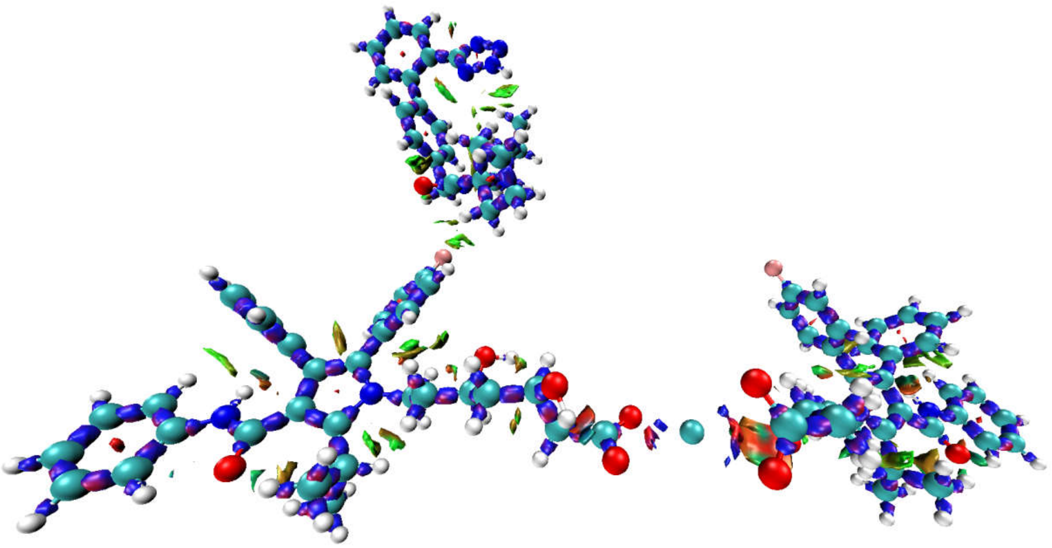

Figure S19. Colored contour surface of the interaction region indicator (IRI) highlighting weak and strong interactions in the ATR-IRB complex **I** (B3LYP/6-311++G(d,p)//B3LYP/6-31Gd,p) level of theory; green, vdW interactions).

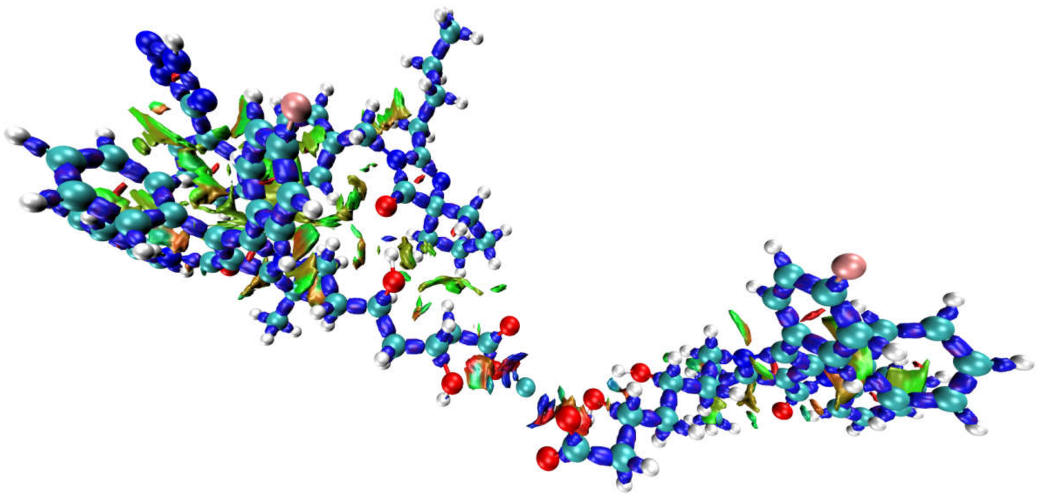

Figure S20. Colored contour surface of the interaction region indicator (IRI) highlighting weak and strong interactions in the ATR-IRB complex **III** (B3LYP/6-311++G(d,p)//B3LYP/6-31Gd,p) level of theory; green, vdW interactions).

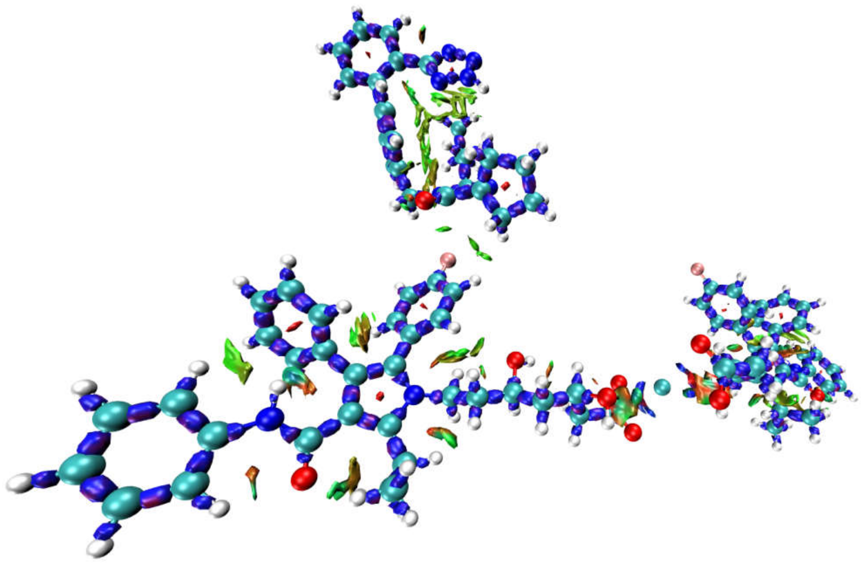

Figure S21. Colored contour surface of the interaction region indicator (IRI) highlighting weak and strong interactions in the ATR-IRB complex **V** (B97D3/6-311++G(d,p)//B97D3/6-31Gd,p) level of theory; green, vdW interactions).

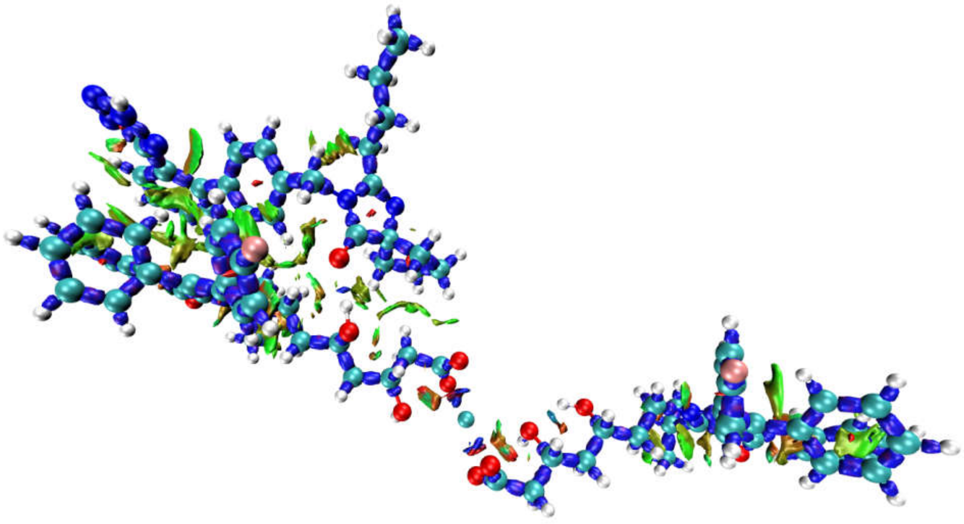

Figure S22. Colored contour surface of the interaction region indicator (IRI) highlighting weak and strong interactions in the ATR-IRB complex **VII** (B97D3/6-311++G(d,p)//B97D3/6-31Gd,p) level of theory; green, vdW interactions).

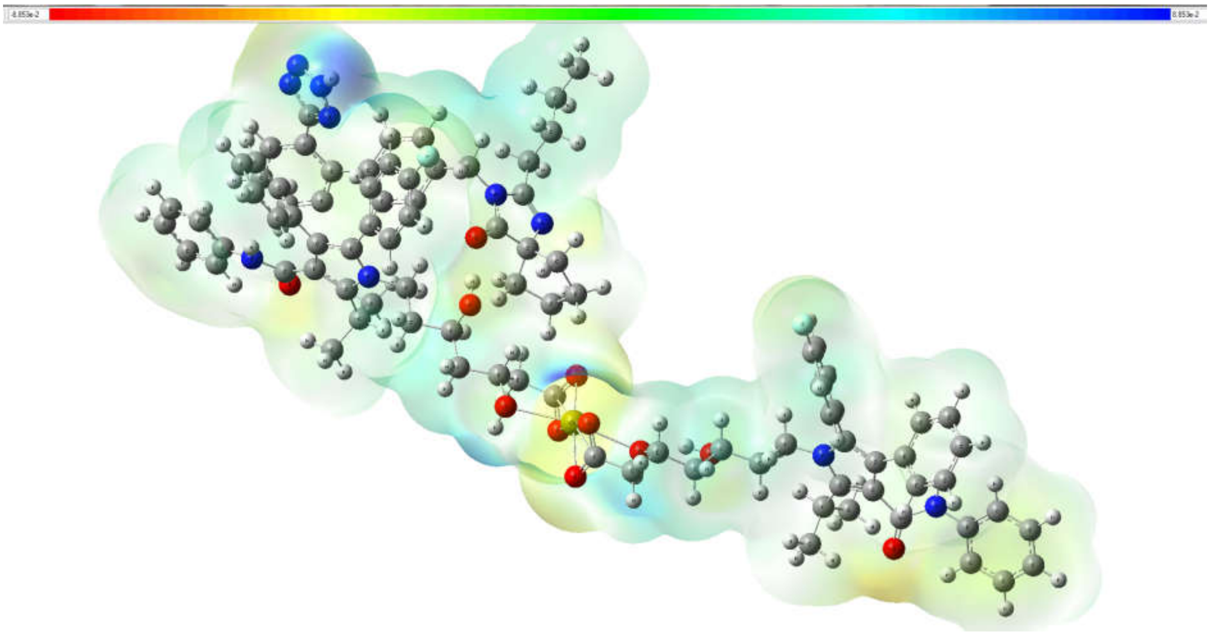

Figure S23. Electrostatic potential (ESP) map of ATR-IRB complex **III** calculated in the gaseous phase (B3LYP/6-311++G(d,p)//B3LYP/6-31Gd,p) level of theory; isovalue = 0.002 a.u).

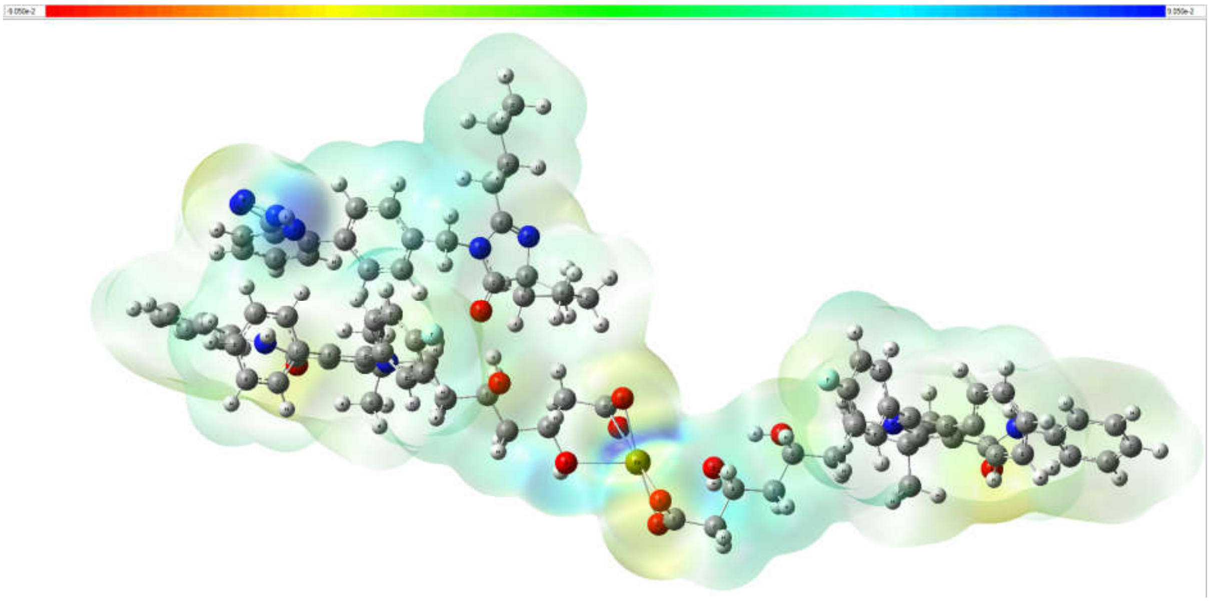

Figure S24. Electrostatic potential (ESP) map of ATR-IRB complex **VII** calculated in the gaseous phase (B97D3/6-311++G(d,p)//B97D3/6-31Gd,p); isovalue = 0.002 a.u).

Cartesian coordinates of the optimized IRB, IRB dimers, ATR calcium, and ATR-IRB complexes.

H	6.768834000	-2.553126000	-0.629226000
H	7.352787000	-0.266287000	-1.417346000
H	1.114655000	0.449252000	-0.877650000

IRB (dimer; tetrazole 1*H* tautomer)

N	1.192000000	7.343000000	-2.528000000
C	0.764000000	6.666000000	-3.690000000
N	1.047000000	7.269000000	-4.781000000
C	1.766000000	8.509000000	-4.440000000
C	1.786000000	8.540000000	-2.916000000
C	1.093000000	9.762000000	-5.084000000
H	0.201000000	9.425000000	-5.617000000
H	0.780000000	10.478000000	-4.320000000
C	2.131000000	10.357000000	-6.061000000
H	1.662000000	10.787000000	-6.951000000
H	2.690000000	11.159000000	-5.566000000
C	3.074000000	9.187000000	-6.368000000
H	4.036000000	9.500000000	-6.784000000
H	2.613000000	8.489000000	-7.073000000
C	3.212000000	8.502000000	-5.007000000
H	3.836000000	9.111000000	-4.341000000
H	3.619000000	7.494000000	-5.058000000
C	0.991000000	6.999000000	-1.124000000
H	1.839000000	7.439000000	-0.592000000
H	1.049000000	5.915000000	-1.017000000
C	-0.297000000	7.531000000	-0.532000000
C	-1.345000000	6.684000000	-0.159000000
H	-1.252000000	5.612000000	-0.306000000
C	-2.500000000	7.199000000	0.430000000
H	-3.295000000	6.528000000	0.741000000
C	-2.632000000	8.578000000	0.652000000
C	-1.585000000	9.422000000	0.254000000
H	-1.678000000	10.492000000	0.415000000
C	-0.434000000	8.909000000	-0.329000000
H	0.375000000	9.569000000	-0.624000000
C	-3.801000000	9.147000000	1.371000000
C	-5.131000000	8.942000000	0.970000000
C	-6.184000000	9.457000000	1.745000000
H	-7.199000000	9.279000000	1.411000000
C	-5.928000000	10.189000000	2.898000000
H	-6.752000000	10.581000000	3.484000000
C	-4.608000000	10.423000000	3.286000000
H	-4.392000000	10.999000000	4.181000000
C	-3.562000000	9.904000000	2.527000000
H	-2.535000000	10.055000000	2.842000000
C	-5.499000000	8.246000000	-0.261000000
N	-4.737000000	8.053000000	-1.360000000
N	-5.466000000	7.429000000	-2.304000000
N	-6.644000000	7.252000000	-1.786000000
N	-6.701000000	7.747000000	-0.527000000
O	2.231000000	9.405000000	-2.155000000
N	-3.248000000	5.046000000	-6.728000000
C	-2.815000000	5.980000000	-5.752000000
N	-3.182000000	5.692000000	-4.567000000
C	-3.971000000	4.458000000	-4.618000000
C	-3.935000000	4.033000000	-6.076000000
C	-3.400000000	3.371000000	-3.660000000
H	-2.760000000	3.888000000	-2.938000000
H	-2.788000000	2.634000000	-4.187000000

B3LYP functional

IRB (tetrazole 1*H* tautomer)

O	-3.400253000	-2.545023000	-0.893608000
N	-3.727539000	0.764418000	0.406804000
N	-2.623902000	-1.170537000	0.800801000
N	1.727823000	1.250999000	-0.923775000
N	3.407713000	2.592780000	-0.978310000
N	1.230023000	2.496645000	-1.050964000
N	2.250138000	3.286986000	-1.080345000
C	-4.158586000	-0.199573000	-0.615813000
C	-3.892163000	0.346907000	-2.032258000
C	-5.687438000	-0.380108000	-0.613485000
C	-6.220113000	0.852173000	-1.373852000
C	-5.054225000	1.327993000	-2.286089000
C	-3.389746000	-1.477385000	-0.312744000
C	-2.869602000	0.180156000	1.152354000
C	-2.117995000	0.840297000	2.262921000
C	-1.728495000	-2.135673000	1.426777000
C	-0.813282000	1.492720000	1.766738000
C	-0.290637000	-1.956906000	0.993658000
C	0.057151000	2.007483000	2.913795000
C	0.054155000	-2.117726000	-0.352795000
C	0.697080000	-1.581555000	1.901456000
C	1.390208000	2.587118000	2.437725000
C	1.354710000	-1.869070000	-0.780863000
C	1.995943000	-1.316739000	1.472978000
C	2.334169000	-1.437233000	0.124098000
C	3.707494000	-1.087730000	-0.339049000
C	4.039015000	0.204794000	-0.792064000
C	4.706020000	-2.063849000	-0.288141000
C	5.360862000	0.483744000	-1.172628000
C	6.011499000	-1.779480000	-0.674738000
C	3.078452000	1.310992000	-0.881132000
C	6.338525000	-0.498832000	-1.116182000
H	-3.926075000	-0.498382000	-2.725244000
H	-2.908782000	0.814235000	-2.106812000
H	-6.081952000	-0.467391000	0.400152000
H	-5.912876000	-1.306113000	-1.149621000
H	-6.489757000	1.639795000	-0.668961000
H	-7.117955000	0.604125000	-1.943663000
H	-5.332521000	1.344010000	-3.341783000
H	-4.753496000	2.340247000	-2.013144000
H	-1.892790000	0.115642000	3.051449000
H	-2.770181000	1.601400000	2.695012000
H	-2.097020000	-3.118048000	1.122734000
H	-1.815446000	-2.062689000	2.511754000
H	-1.061805000	2.306757000	1.080819000
H	-0.243052000	0.764307000	1.186917000
H	0.245854000	1.185914000	3.616677000
H	-0.498007000	2.766789000	3.476712000
H	-0.703028000	-2.431740000	-1.062534000
H	0.445076000	-1.457650000	2.948958000
H	1.980710000	2.963437000	3.276880000
H	1.239119000	3.410018000	1.735935000
H	1.987055000	1.825822000	1.927170000
H	1.614766000	-1.994646000	-1.826283000
H	2.746158000	-0.988636000	2.182542000
H	4.445685000	-3.054894000	0.064354000
H	5.598242000	1.483890000	-1.510406000

IRB (tetrazole **2*H*** tautomer)

O	-3.754621000	-2.203174000	-1.458348000
N	-3.529027000	0.662028000	0.659775000
N	-2.703170000	-1.435817000	0.460745000
N	1.667187000	1.484770000	-0.811658000
N	3.387138000	2.789154000	-0.261775000
N	1.315308000	2.735857000	-0.552872000
N	2.320699000	3.534546000	-0.222456000
C	-4.155233000	0.056833000	-0.525126000
C	-3.900173000	0.909250000	-1.782326000
C	-5.691456000	0.073094000	-0.422813000
C	-6.094756000	1.508322000	-0.826156000
C	-4.908866000	2.067257000	-1.662413000
C	-3.561150000	-1.342293000	-0.623994000
C	-2.727780000	-0.204730000	1.154005000
C	-1.851733000	0.033278000	2.343465000
C	-1.827509000	-2.582110000	0.684109000
C	-0.572907000	0.814769000	1.988408000
C	-0.390437000	-2.261535000	0.341243000
C	0.395313000	0.895958000	3.169407000
C	-0.074334000	-1.807347000	-0.943048000
C	0.619258000	-2.333217000	1.296888000
C	1.702208000	1.609983000	2.825034000
C	1.211193000	-1.385039000	-1.244952000
C	1.913421000	-1.912886000	0.992952000
C	2.216628000	-1.407737000	-0.272555000
C	3.597173000	-0.938912000	-0.577830000
C	3.939314000	0.418646000	-0.754551000
C	4.611848000	-1.899522000	-0.657473000
C	5.275576000	0.764244000	-1.008743000
C	5.931189000	-1.546626000	-0.918060000
C	2.984992000	1.528805000	-0.619540000
C	6.263558000	-0.206128000	-1.097077000
H	0.355790000	3.051478000	-0.585073000
H	-4.111746000	0.285156000	-2.655057000
H	-2.861409000	1.239520000	-1.843210000
H	-6.030329000	-0.206413000	0.575852000
H	-6.078994000	-0.661788000	-1.133566000
H	-6.250975000	2.121707000	0.062288000
H	-7.030837000	1.507661000	-1.388398000
H	-5.223205000	2.424859000	-2.645121000
H	-4.449152000	2.908084000	-1.140521000
H	-1.580874000	-0.918582000	2.806604000
H	-2.433385000	0.598317000	3.075249000
H	-2.219130000	-3.372084000	0.039243000
H	-1.914519000	-2.918397000	1.719265000
H	-0.864629000	1.821503000	1.670326000
H	-0.073688000	0.345919000	1.138068000
H	0.619228000	-0.121351000	3.511357000
H	-0.096461000	1.400808000	4.009426000
H	-0.849256000	-1.764240000	-1.700279000
H	0.391971000	-2.687742000	2.296705000
H	2.364152000	1.669572000	3.692565000
H	1.521940000	2.631304000	2.478504000
H	2.237426000	1.078241000	2.035141000
H	1.437953000	-1.009807000	-2.234691000
H	2.685751000	-1.945410000	1.752789000
H	4.346504000	-2.940867000	-0.518080000
H	5.524873000	1.810730000	-1.125566000
H	6.693558000	-2.314235000	-0.981811000
H	7.287454000	0.083539000	-1.300646000

C	-4.623000000	2.766000000	-2.957000000
H	-4.364000000	2.277000000	-2.012000000
H	-5.089000000	2.016000000	-3.607000000
C	-5.556000000	3.971000000	-2.798000000
H	-6.586000000	3.702000000	-2.548000000
H	-5.187000000	4.635000000	-2.008000000
C	-5.448000000	4.678000000	-4.156000000
H	-6.114000000	4.195000000	-4.879000000
H	-5.678000000	5.740000000	-4.110000000
C	-2.967000000	4.974000000	-8.155000000
H	-3.774000000	4.362000000	-8.571000000
H	-3.038000000	5.971000000	-8.593000000
C	-1.635000000	4.338000000	-8.483000000
C	-0.673000000	5.006000000	-9.244000000
H	-0.875000000	6.009000000	-9.608000000
C	0.538000000	4.389000000	-9.560000000
H	1.269000000	4.910000000	-10.170000000
C	0.819000000	3.099000000	-9.094000000
C	-0.154000000	2.431000000	-8.335000000
H	0.050000000	1.427000000	-7.979000000
C	-1.366000000	3.039000000	-8.036000000
H	-2.119000000	2.505000000	-7.467000000
C	2.084000000	2.397000000	-9.432000000
C	3.353000000	2.855000000	-9.041000000
C	4.498000000	2.130000000	-9.416000000
H	5.464000000	2.504000000	-9.099000000
C	4.393000000	0.962000000	-10.160000000
H	5.289000000	0.418000000	-10.439000000
C	3.133000000	0.492000000	-10.534000000
H	3.035000000	-0.423000000	-11.111000000
C	1.997000000	1.207000000	-10.169000000
H	1.014000000	0.859000000	-10.468000000
C	3.575000000	4.041000000	-8.212000000
N	2.662000000	4.853000000	-7.626000000
N	3.302000000	5.818000000	-6.935000000
N	4.569000000	5.584000000	-7.093000000
N	4.774000000	4.498000000	-7.871000000
C	-2.007000000	7.195000000	-6.092000000
H	-0.955000000	7.021000000	-5.825000000
H	-2.020000000	7.353000000	-7.173000000
C	-2.517000000	8.462000000	-5.382000000
H	-2.029000000	9.326000000	-5.850000000
H	-3.592000000	8.578000000	-5.568000000
C	-2.254000000	8.493000000	-3.871000000
H	-2.765000000	7.637000000	-3.418000000
H	-1.184000000	8.347000000	-3.690000000
C	-2.703000000	9.809000000	-3.233000000
H	-2.461000000	9.841000000	-2.165000000
H	-2.202000000	10.664000000	-3.701000000
H	-3.783000000	9.958000000	-3.343000000
O	-4.389000000	3.045000000	-6.601000000
C	0.088000000	5.327000000	-3.660000000
H	-0.165000000	5.036000000	-2.638000000
H	-0.866000000	5.418000000	-4.192000000
C	0.966000000	4.253000000	-4.327000000
H	1.832000000	4.038000000	-3.687000000
H	1.375000000	4.674000000	-5.250000000
C	0.212000000	2.959000000	-4.637000000
H	-0.609000000	3.183000000	-5.330000000
H	-0.256000000	2.571000000	-3.723000000
C	1.121000000	1.889000000	-5.246000000
H	1.925000000	1.614000000	-4.554000000
H	0.564000000	0.979000000	-5.489000000
H	1.591000000	2.250000000	-6.167000000
H	1.652000000	4.844000000	-7.678000000
H	-3.766000000	8.277000000	-1.531000000

C	-3.641764000	4.221623000	-7.933533000
H	-4.307942000	3.368717000	-8.090494000
H	-3.977640000	5.036875000	-8.578263000
C	-2.217317000	3.836573000	-8.255335000
C	-1.419117000	4.648941000	-9.065838000
H	-1.829584000	5.565783000	-9.481212000
C	-0.110941000	4.284540000	-9.366426000
H	0.493784000	4.919760000	-10.004707000
C	0.434728000	3.097475000	-8.858901000
C	-0.374612000	2.278545000	-8.059652000
H	0.035114000	1.357626000	-7.658328000
C	-1.684023000	2.642132000	-7.757512000
H	-2.301881000	2.005154000	-7.131954000
C	1.820240000	2.691388000	-9.205636000
C	2.917354000	3.574105000	-9.095871000
C	4.180908000	3.174021000	-9.549108000
H	5.004876000	3.874512000	-9.467636000
C	4.380222000	1.900379000	-10.073571000
H	5.366273000	1.603939000	-10.416527000
C	3.310553000	1.008047000	-10.144236000
H	3.455396000	0.009935000	-10.545752000
C	2.045690000	1.406565000	-9.718629000
H	1.202896000	0.729531000	-9.815846000
C	2.794184000	4.901607000	-8.475919000
N	2.088347000	5.156260000	-7.368569000
N	2.304606000	6.453777000	-7.200895000
H	1.946259000	6.964037000	-6.349463000
N	3.099329000	6.991424000	-8.113432000
N	3.421385000	6.017163000	-8.939201000
C	-3.344471000	7.087803000	-6.786756000
H	-2.427156000	6.805164000	-7.319108000
H	-4.094844000	7.287506000	-7.563590000
C	-3.102882000	8.344433000	-5.935721000
H	-2.946789000	9.190013000	-6.615485000
H	-4.009744000	8.575099000	-5.366023000
C	-1.903519000	8.223255000	-4.987108000
H	-2.018008000	7.320085000	-4.381401000
H	-0.988714000	8.093514000	-5.578489000
C	-1.750934000	9.421178000	-4.048950000
H	-0.872254000	9.302575000	-3.408164000
H	-1.638159000	10.358307000	-4.606123000
H	-2.618416000	9.510603000	-3.388466000
O	-4.382169000	2.524029000	-5.697549000
C	0.251225000	5.528300000	-4.127916000
H	-0.531323000	5.447579000	-3.366237000
H	-0.241927000	5.682860000	-5.090229000
C	1.086814000	4.234851000	-4.199485000
H	1.644650000	4.086540000	-3.265592000
H	1.821653000	4.352528000	-5.001327000
C	0.218414000	3.009528000	-4.491268000
H	-0.430432000	3.230357000	-5.346602000
H	-0.442687000	2.813382000	-3.636579000
C	1.051540000	1.765812000	-4.801655000
H	1.715751000	1.511367000	-3.968148000
H	0.413181000	0.897938000	-4.994870000
H	1.673853000	1.931954000	-5.686998000

ATR calcium (ATR-Ca-ATR)

Ca	1.985203000	34.787057000	8.644625000
O	1.618249000	32.464914000	8.405370000
C	2.381979000	32.356884000	7.343699000
O	2.985031000	33.403998000	6.895045000
C	2.539689000	30.993818000	6.715430000
H	3.015302000	31.109675000	5.739131000

IRB (**dimer**; tetrazole **2*H*** tautomer)

N	1.503396000	7.060283000	-2.563376000
C	1.100344000	6.726398000	-3.855685000
N	1.596207000	7.494046000	-4.767826000
C	2.454412000	8.495778000	-4.114183000
C	2.344568000	8.167326000	-2.630503000
C	1.994307000	9.951511000	-4.450172000
H	1.171127000	9.881089000	-5.165151000
H	1.621904000	10.466222000	-3.561479000
C	3.217331000	10.651663000	-5.075502000
H	2.928815000	11.413968000	-5.804590000
H	3.801631000	11.147169000	-4.291805000
C	4.036222000	9.505475000	-5.682591000
H	5.074571000	9.779078000	-5.890689000
H	3.593669000	9.160853000	-6.623452000
C	3.925179000	8.413743000	-4.611670000
H	4.577118000	8.653210000	-3.764345000
H	4.173048000	7.409278000	-4.963216000
C	1.088132000	6.453295000	-1.299443000
H	1.923808000	6.619062000	-0.613190000
H	0.980136000	5.376811000	-1.445954000
C	-0.180219000	7.052656000	-0.733800000
C	-1.312006000	6.265777000	-0.497938000
H	-1.289520000	5.204709000	-0.734590000
C	-2.461056000	6.822747000	0.058330000
H	-3.330852000	6.200211000	0.241244000
C	-2.508119000	8.184194000	0.382019000
C	-1.371392000	8.967247000	0.144492000
H	-1.396831000	10.023734000	0.392135000
C	-0.219881000	8.411908000	-0.404012000
H	0.659942000	9.026603000	-0.563277000
C	-3.701300000	8.798426000	1.026606000
C	-4.987034000	8.825642000	0.441430000
C	-6.057498000	9.391169000	1.152092000
H	-7.035825000	9.396817000	0.685508000
C	-5.870118000	9.942236000	2.414411000
H	-6.711648000	10.376346000	2.944835000
C	-4.597997000	9.942826000	2.985951000
H	-4.436287000	10.375590000	3.968308000
C	-3.531967000	9.375092000	2.293369000
H	-2.544044000	9.351096000	2.742387000
C	-5.269953000	8.308915000	-0.903585000
N	-4.364932000	8.062023000	-1.856560000
N	-5.125211000	7.633454000	-2.854545000
H	-4.712581000	7.150280000	-3.692316000
N	-6.419968000	7.588314000	-2.566860000
N	-6.534808000	8.025409000	-1.334869000
O	2.861236000	8.726310000	-1.681112000
N	-3.846893000	4.620654000	-6.540911000
C	-3.794093000	5.901732000	-5.987637000
N	-4.138554000	5.939780000	-4.747874000
C	-4.420767000	4.570444000	-4.295263000
C	-4.234772000	3.723034000	-5.549931000
C	-3.423249000	4.163227000	-3.162756000
H	-3.027493000	5.088991000	-2.733236000
H	-2.583779000	3.580417000	-3.548286000
C	-4.271484000	3.418278000	-2.124480000
H	-3.805818000	3.412175000	-1.134005000
H	-4.414281000	2.377540000	-2.436457000
C	-5.607531000	4.169003000	-2.169330000
H	-6.430201000	3.624788000	-1.697892000
H	-5.517327000	5.133775000	-1.658681000
C	-5.838822000	4.384011000	-3.673850000
H	-6.282960000	3.484637000	-4.111866000
H	-6.486681000	5.233958000	-3.895727000

C	3.834488000	24.107729000	6.264705000
H	4.582096000	24.429620000	6.998474000
H	3.662905000	23.034793000	6.377029000
H	2.896083000	24.633094000	6.479624000
O	1.717489000	35.717319000	10.862039000
C	0.760617000	36.417650000	10.327900000
O	0.040679000	35.956442000	9.355645000
C	0.784596000	37.908788000	10.516116000
H	-0.218913000	38.336818000	10.532179000
H	1.323320000	38.183737000	11.426421000
C	1.526041000	38.423127000	9.254533000
H	0.877960000	38.299068000	8.385313000
O	2.681250000	37.485388000	8.926009000
H	3.266867000	37.465490000	9.712908000
C	2.024645000	39.854603000	9.392029000
H	2.742469000	39.924233000	10.218655000
H	1.157464000	40.474266000	9.667862000
C	2.676990000	40.430773000	8.128618000
H	2.007242000	40.258768000	7.268466000
O	3.963468000	39.797350000	7.869193000
H	3.824407000	38.828616000	7.830962000
C	2.933731000	41.926509000	8.272086000
H	3.518595000	42.103169000	9.181344000
H	1.974314000	42.447922000	8.395665000
C	3.671100000	42.510854000	7.056117000
H	4.728143000	42.241529000	7.098106000
H	3.273897000	42.089078000	6.131641000
N	3.533121000	43.973288000	6.963678000
C	2.777403000	44.626944000	5.975260000
C	2.966757000	45.989210000	6.137019000
C	3.838850000	46.172731000	7.281690000
C	4.198717000	44.897651000	7.754112000
C	1.880499000	43.927601000	5.040169000
C	0.892115000	43.028179000	5.506676000
H	0.789504000	42.861481000	6.571220000
C	0.025838000	42.385102000	4.629473000
H	-0.733669000	41.694852000	4.978787000
C	0.142755000	42.655056000	3.269623000
C	1.084769000	43.546064000	2.762423000
H	1.137693000	43.728489000	1.698312000
C	1.954772000	44.171244000	3.660169000
H	2.708424000	44.850029000	3.283489000
F	-0.722485000	42.017887000	2.386556000
C	2.278077000	47.068628000	5.399999000
C	2.747331000	47.497748000	4.130719000
H	3.602194000	47.031386000	3.696247000
C	2.114683000	48.544653000	3.454448000
H	2.480324000	48.874515000	2.501306000
C	1.009073000	49.178660000	4.040719000
H	0.513257000	50.019804000	3.513328000
C	0.530012000	48.769924000	5.304358000
H	-0.318035000	49.256068000	5.738334000
C	1.176119000	47.716026000	5.957688000
H	0.807311000	47.398684000	6.932444000
C	4.254883000	47.432504000	7.827281000
O	4.532583000	47.643742000	9.111263000
N	4.410271000	48.497903000	6.957949000
H	4.240450000	48.261577000	5.981927000
C	4.779794000	49.829812000	7.202854000
C	5.164524000	50.608479000	6.102774000
H	5.200064000	50.148730000	5.113841000
C	5.518463000	51.974262000	6.252097000
H	5.878308000	52.480760000	5.403849000
C	5.541534000	52.510549000	7.546508000
H	5.854926000	53.538756000	7.686706000
C	5.126664000	51.752467000	8.629317000

H	3.217219000	30.408593000	7.354480000
C	1.203651000	30.213225000	6.597719000
H	0.560341000	30.732178000	5.861833000
O	0.514939000	30.134076000	7.872529000
H	0.675125000	30.978265000	8.364609000
C	1.397912000	28.751174000	6.191511000
H	0.413131000	28.272099000	6.263717000
H	2.032017000	28.284561000	6.958777000
C	1.981925000	28.452686000	4.812336000
H	2.958298000	28.950299000	4.685774000
O	1.090882000	28.865537000	3.730106000
H	0.939150000	29.833754000	3.771283000
C	2.146444000	26.949199000	4.653624000
H	2.803614000	26.573876000	5.465933000
H	1.150848000	26.479575000	4.728911000
C	2.779112000	26.521601000	3.318398000
H	2.329509000	27.056534000	2.484940000
H	3.848693000	26.769571000	3.307733000
N	2.626819000	25.076725000	3.085283000
C	1.781000000	24.511884000	2.130785000
C	1.920815000	23.135228000	2.210057000
C	2.852949000	22.844497000	3.260006000
C	3.294847000	24.075654000	3.779010000
C	0.872183000	25.290894000	1.255418000
C	0.059816000	26.330865000	1.755631000
H	0.089646000	26.593070000	2.801437000
C	-0.787976000	27.032394000	0.905636000
H	-1.418904000	27.831915000	1.279200000
C	-0.858190000	26.671256000	-0.434924000
C	-0.085446000	25.640195000	-0.965045000
H	-0.160789000	25.393980000	-2.012683000
C	0.789076000	24.969081000	-0.115584000
H	1.412589000	24.183377000	-0.509558000
F	-1.736709000	27.365526000	-1.276982000
C	1.106251000	22.129954000	1.455762000
C	1.540019000	21.593994000	0.242635000
H	2.458569000	21.951689000	-0.193395000
C	0.778559000	20.631724000	-0.417156000
H	1.111765000	20.246419000	-1.385243000
C	-0.414114000	20.156764000	0.142314000
H	-1.000378000	19.401601000	-0.366888000
C	-0.839937000	20.674287000	1.360652000
H	-1.767864000	20.324608000	1.805706000
C	-0.085081000	21.653647000	2.030942000
H	-0.418499000	22.083965000	2.990551000
C	3.299556000	21.485790000	3.732604000
O	3.557861000	21.298951000	4.966836000
N	3.291729000	20.510630000	2.810318000
H	3.051398000	20.777418000	1.872981000
C	3.648997000	19.161837000	3.000238000
C	4.057171000	18.427406000	1.869911000
H	4.088818000	18.951470000	0.892424000
C	4.420056000	17.092819000	1.979416000
H	4.746282000	16.547139000	1.083685000
C	4.387660000	16.450172000	3.216444000
H	4.678209000	15.415182000	3.310964000
C	3.970763000	17.178928000	4.351944000
H	3.942144000	16.688178000	5.315910000
C	3.608197000	18.519487000	4.257191000
H	3.308744000	19.078705000	5.124368000
C	4.338550000	24.393263000	4.834903000
H	4.549472000	25.471349000	4.767682000
C	5.679728000	23.670187000	4.570459000
H	6.436485000	24.018052000	5.284636000
H	6.042422000	23.866419000	3.555887000
H	5.569418000	22.595228000	4.699920000

H	-4.659000000	26.561000000	-4.524000000
H	-3.275000000	29.078000000	-7.710000000
H	-5.451000000	32.845000000	-9.017000000
H	-6.339000000	32.922000000	-7.496000000
H	-6.036000000	31.357000000	-8.262000000
H	-6.695000000	26.018000000	-5.846000000
H	-5.320000000	28.565000000	-9.011000000
H	-6.719000000	24.958000000	-8.695000000
H	-10.254000000	28.324000000	-9.508000000
H	-8.671000000	24.369000000	-10.109000000
H	-10.434000000	26.075000000	-10.550000000
Ca	1.543000000	34.657000000	8.835000000
O	2.237000000	32.417000000	8.742000000
C	2.450000000	32.532000000	7.481000000
O	2.440000000	33.658000000	6.894000000
C	2.601000000	31.246000000	6.699000000
H	3.104000000	31.437000000	5.749000000
H	3.211000000	30.559000000	7.294000000
C	1.202000000	30.611000000	6.458000000
H	0.749000000	31.098000000	5.583000000
O	0.306000000	30.856000000	7.536000000
H	0.836000000	31.020000000	8.332000000
C	1.298000000	29.104000000	6.199000000
H	0.278000000	28.719000000	6.092000000
H	1.732000000	28.632000000	7.090000000
C	2.109000000	28.696000000	4.966000000
H	3.150000000	29.042000000	5.079000000
O	1.548000000	29.236000000	3.763000000
H	1.707000000	30.186000000	3.748000000
C	2.120000000	27.181000000	4.789000000
H	2.453000000	26.716000000	5.720000000
H	1.093000000	26.841000000	4.619000000
C	3.006000000	26.735000000	3.622000000
H	2.735000000	27.293000000	2.728000000
H	4.056000000	26.959000000	3.836000000
N	2.877000000	25.311000000	3.317000000
C	2.254000000	24.803000000	2.174000000
C	2.389000000	23.426000000	2.207000000
C	3.102000000	23.085000000	3.414000000
C	3.400000000	24.283000000	4.075000000
C	1.569000000	25.642000000	1.174000000
C	0.619000000	26.613000000	1.538000000
H	0.368000000	26.760000000	2.581000000
C	-0.015000000	27.403000000	0.584000000
H	-0.731000000	28.163000000	0.872000000
C	0.283000000	27.200000000	-0.757000000
C	1.198000000	26.236000000	-1.163000000
H	1.408000000	26.107000000	-2.219000000
C	1.843000000	25.473000000	-0.194000000
H	2.578000000	24.738000000	-0.500000000
F	-0.317000000	27.969000000	-1.694000000
C	1.810000000	22.480000000	1.220000000
C	2.527000000	22.104000000	0.074000000
H	3.504000000	22.545000000	-0.104000000
C	2.012000000	21.159000000	-0.815000000
H	2.581000000	20.879000000	-1.696000000
C	0.770000000	20.573000000	-0.568000000
H	0.370000000	19.833000000	-1.253000000
C	0.047000000	20.940000000	0.569000000
H	-0.918000000	20.484000000	0.768000000
C	0.562000000	21.884000000	1.454000000
H	0.009000000	22.160000000	2.346000000
C	3.432000000	21.733000000	3.926000000
O	3.733000000	21.520000000	5.105000000
N	3.386000000	20.723000000	2.994000000
H	3.132000000	20.988000000	2.053000000

H	5.114133000	52.181930000	9.631313000
C	4.762919000	50.408768000	8.489219000
H	4.483009000	49.814512000	9.340791000
C	5.117873000	44.470657000	8.867648000
H	5.296480000	43.397445000	8.740862000
C	6.496799000	45.159552000	8.794267000
H	7.160973000	44.720065000	9.547327000
H	6.408775000	46.230769000	8.995202000
H	6.955445000	45.015434000	7.806232000
C	4.468621000	44.679321000	10.254031000
H	3.509569000	44.158524000	10.350949000
H	4.307990000	45.740642000	10.412878000
H	5.133658000	44.311259000	11.047231000

Complex **I**

O	-3.252000000	27.382000000	-2.477000000
N	-3.522000000	30.933000000	-2.833000000
N	-3.002000000	29.084000000	-4.038000000
N	-7.792000000	29.551000000	-6.732000000
N	-9.125000000	30.288000000	-8.371000000
N	-8.180000000	30.811000000	-6.573000000
N	-8.969000000	31.281000000	-7.534000000
C	-3.598000000	29.779000000	-1.921000000
C	-4.995000000	29.691000000	-1.243000000
C	-2.627000000	29.949000000	-0.735000000
C	-3.398000000	30.871000000	0.211000000
C	-4.826000000	30.294000000	0.178000000
C	-3.271000000	28.563000000	-2.781000000
C	-3.199000000	30.480000000	-3.990000000
C	-3.089000000	31.326000000	-5.221000000
C	-2.644000000	28.237000000	-5.173000000
C	-4.444000000	31.508000000	-5.934000000
C	-3.839000000	27.864000000	-6.023000000
C	-4.287000000	32.207000000	-7.288000000
C	-4.805000000	26.989000000	-5.511000000
C	-4.025000000	28.411000000	-7.295000000
C	-5.601000000	32.340000000	-8.058000000
C	-5.944000000	26.689000000	-6.251000000
C	-5.174000000	28.123000000	-8.030000000
C	-6.151000000	27.267000000	-7.510000000
C	-7.376000000	26.958000000	-8.298000000
C	-8.396000000	27.908000000	-8.519000000
C	-7.505000000	25.687000000	-8.871000000
C	-9.484000000	27.578000000	-9.343000000
C	-8.597000000	25.361000000	-9.673000000
C	-8.404000000	29.232000000	-7.879000000
C	-9.583000000	26.316000000	-9.920000000
H	-7.862000000	31.383000000	-5.803000000
H	-5.306000000	28.645000000	-1.197000000
H	-5.722000000	30.236000000	-1.847000000
H	-1.654000000	30.327000000	-1.055000000
H	-2.484000000	28.961000000	-0.286000000
H	-3.392000000	31.888000000	-0.194000000
H	-2.972000000	30.905000000	1.219000000
H	-4.927000000	29.504000000	0.928000000
H	-5.585000000	31.046000000	0.400000000
H	-2.365000000	30.890000000	-5.917000000
H	-2.701000000	32.302000000	-4.916000000
H	-2.193000000	27.344000000	-4.732000000
H	-1.882000000	28.743000000	-5.771000000
H	-5.101000000	32.092000000	-5.277000000
H	-4.922000000	30.535000000	-6.079000000
H	-3.570000000	31.638000000	-7.896000000
H	-3.838000000	33.197000000	-7.137000000

H	0.944000000	49.909000000	3.461000000
C	1.029000000	48.801000000	5.311000000
H	0.314000000	49.440000000	5.821000000
C	1.594000000	47.722000000	5.984000000
H	1.329000000	47.522000000	7.018000000
C	4.585000000	47.146000000	7.821000000
O	4.956000000	47.231000000	8.994000000
N	4.624000000	48.217000000	6.955000000
H	4.292000000	48.040000000	6.018000000
C	4.840000000	49.575000000	7.244000000
C	4.502000000	50.496000000	6.237000000
H	4.075000000	50.137000000	5.301000000
C	4.692000000	51.858000000	6.442000000
H	4.429000000	52.558000000	5.653000000
C	5.207000000	52.325000000	7.651000000
H	5.354000000	53.388000000	7.812000000
C	5.537000000	51.408000000	8.649000000
H	5.945000000	51.757000000	9.596000000
C	5.364000000	50.037000000	8.459000000
H	5.617000000	49.324000000	9.229000000
C	5.249000000	44.091000000	8.770000000
H	5.322000000	43.006000000	8.642000000
C	6.682000000	44.640000000	8.661000000
H	7.319000000	44.159000000	9.410000000
H	6.699000000	45.716000000	8.838000000
H	7.105000000	44.437000000	7.672000000
C	4.650000000	44.351000000	10.166000000
H	3.650000000	43.915000000	10.256000000
H	4.578000000	45.424000000	10.339000000
H	5.288000000	43.904000000	10.937000000

Complex **II**

O	1.879000000	30.341000000	9.039000000
N	3.029000000	31.298000000	12.294000000
N	1.635000000	29.823000000	11.276000000
N	3.971000000	24.207000000	12.933000000
N	4.027000000	22.046000000	13.507000000
N	4.380000000	23.999000000	14.180000000
N	4.421000000	22.724000000	14.552000000
C	3.147000000	31.500000000	10.846000000
C	4.603000000	31.273000000	10.360000000
C	2.900000000	32.966000000	10.451000000
C	4.199000000	33.663000000	10.864000000
C	5.309000000	32.650000000	10.493000000
C	2.164000000	30.522000000	10.230000000
C	2.187000000	30.351000000	12.478000000
C	1.796000000	29.796000000	13.809000000
C	0.632000000	28.770000000	11.106000000
C	2.519000000	28.476000000	14.140000000
C	1.223000000	27.385000000	10.943000000
C	1.961000000	27.816000000	15.404000000
C	2.180000000	27.138000000	9.953000000
C	0.796000000	26.320000000	11.742000000
C	2.628000000	26.473000000	15.702000000
C	2.685000000	25.855000000	9.768000000
C	1.297000000	25.034000000	11.552000000
C	2.246000000	24.781000000	10.555000000
C	2.713000000	23.404000000	10.242000000
C	3.347000000	22.545000000	11.167000000
C	2.486000000	22.927000000	8.942000000
C	3.685000000	21.239000000	10.778000000
C	2.850000000	21.640000000	8.555000000
C	3.751000000	22.956000000	12.519000000
C	3.439000000	20.787000000	9.487000000
H	4.605000000	24.756000000	14.810000000
C	3.543000000	19.340000000	3.182000000
C	3.289000000	18.523000000	2.069000000
H	2.974000000	18.981000000	1.136000000
C	3.428000000	17.143000000	2.161000000
H	3.224000000	16.527000000	1.290000000
C	3.819000000	16.552000000	3.364000000
H	3.930000000	15.476000000	3.439000000
C	4.063000000	17.365000000	4.468000000
H	4.363000000	16.917000000	5.410000000
C	3.935000000	18.751000000	4.393000000
H	4.129000000	19.384000000	5.248000000
C	4.188000000	24.569000000	5.333000000
H	4.340000000	25.652000000	5.369000000
C	5.593000000	23.943000000	5.306000000
H	6.166000000	24.282000000	6.176000000
H	6.136000000	24.241000000	4.404000000
H	5.533000000	22.856000000	5.339000000
C	3.416000000	24.179000000	6.609000000
H	3.978000000	24.491000000	7.496000000
H	3.272000000	23.100000000	6.636000000
H	2.433000000	24.662000000	6.641000000
O	1.234000000	35.735000000	10.924000000
C	0.346000000	36.399000000	10.299000000
O	-0.374000000	35.902000000	9.376000000
C	0.415000000	37.910000000	10.391000000
H	-0.571000000	38.375000000	10.325000000
H	0.908000000	38.218000000	11.317000000
C	1.252000000	38.327000000	9.161000000
H	0.639000000	38.206000000	8.264000000
O	2.352000000	37.373000000	8.940000000
H	2.835000000	37.323000000	9.782000000
C	1.777000000	39.753000000	9.268000000
H	2.426000000	39.842000000	10.149000000
H	0.912000000	40.405000000	9.444000000
C	2.549000000	40.251000000	8.039000000
H	1.932000000	40.072000000	7.139000000
O	3.805000000	39.594000000	7.911000000
H	3.621000000	38.643000000	7.917000000
C	2.828000000	41.746000000	8.139000000
H	3.351000000	41.952000000	9.078000000
H	1.875000000	42.284000000	8.180000000
C	3.649000000	42.269000000	6.956000000
H	4.677000000	41.907000000	7.018000000
H	3.235000000	41.892000000	6.020000000
N	3.641000000	43.729000000	6.866000000
C	2.918000000	44.434000000	5.907000000
C	3.169000000	45.780000000	6.097000000
C	4.065000000	45.894000000	7.221000000
C	4.346000000	44.599000000	7.671000000
C	1.987000000	43.782000000	4.967000000
C	0.832000000	43.130000000	5.432000000
H	0.626000000	43.115000000	6.497000000
C	-0.065000000	42.531000000	4.553000000
H	-0.961000000	42.031000000	4.903000000
C	0.194000000	42.602000000	3.188000000
C	1.320000000	43.243000000	2.686000000
H	1.486000000	43.274000000	1.615000000
C	2.214000000	43.824000000	3.582000000
H	3.108000000	44.309000000	3.208000000
F	-0.674000000	42.031000000	2.329000000
C	2.529000000	46.887000000	5.349000000
C	2.882000000	47.168000000	4.020000000
H	3.626000000	46.551000000	3.528000000
C	2.310000000	48.247000000	3.340000000
H	2.597000000	48.449000000	2.312000000
C	1.384000000	49.067000000	3.985000000

F	-1.752000000	27.939000000	-0.674000000
C	0.980000000	22.686000000	2.150000000
C	1.202000000	22.197000000	0.854000000
H	2.034000000	22.590000000	0.278000000
C	0.383000000	21.201000000	0.316000000
H	0.570000000	20.833000000	-0.689000000
C	-0.668000000	20.680000000	1.069000000
H	-1.303000000	19.902000000	0.653000000
C	-0.899000000	21.161000000	2.359000000
H	-1.716000000	20.757000000	2.950000000
C	-0.081000000	22.152000000	2.897000000
H	-0.251000000	22.520000000	3.904000000
C	3.276000000	22.147000000	4.331000000
O	3.727000000	22.027000000	5.478000000
N	3.105000000	21.074000000	3.492000000
H	2.727000000	21.282000000	2.579000000
C	3.318000000	19.709000000	3.739000000
C	3.007000000	18.825000000	2.692000000
H	2.608000000	19.221000000	1.762000000
C	3.202000000	17.457000000	2.847000000
H	2.956000000	16.792000000	2.024000000
C	3.706000000	16.944000000	4.044000000
H	3.858000000	15.877000000	4.164000000
C	4.003000000	17.824000000	5.085000000
H	4.390000000	17.441000000	6.025000000
C	3.823000000	19.199000000	4.944000000
H	4.061000000	19.884000000	5.743000000
C	4.612000000	25.045000000	4.997000000
H	4.864000000	26.089000000	4.783000000
C	5.872000000	24.216000000	4.694000000
H	6.711000000	24.587000000	5.292000000
H	6.144000000	24.284000000	3.636000000
H	5.707000000	23.168000000	4.947000000
C	4.234000000	24.957000000	6.487000000
H	5.054000000	25.332000000	7.108000000
H	4.029000000	23.921000000	6.755000000
H	3.341000000	25.551000000	6.703000000
O	0.426000000	34.903000000	8.755000000
C	0.184000000	35.573000000	9.837000000
O	-0.566000000	35.248000000	10.752000000
C	0.976000000	36.899000000	9.982000000
H	0.365000000	37.595000000	10.564000000
H	1.877000000	36.696000000	10.578000000
C	1.419000000	37.550000000	8.676000000
H	0.589000000	37.526000000	7.957000000
O	2.508000000	36.779000000	8.087000000
H	3.247000000	37.396000000	7.914000000
C	1.861000000	39.001000000	8.872000000
H	2.612000000	39.055000000	9.671000000
H	0.996000000	39.580000000	9.210000000
C	2.452000000	39.662000000	7.628000000
H	1.806000000	39.470000000	6.759000000
O	3.745000000	39.034000000	7.391000000
H	3.858000000	38.904000000	6.441000000
C	2.617000000	41.170000000	7.812000000
H	3.146000000	41.360000000	8.750000000
H	1.623000000	41.618000000	7.913000000
C	3.356000000	41.838000000	6.648000000
H	4.399000000	41.512000000	6.633000000
H	2.906000000	41.539000000	5.699000000
N	3.307000000	43.296000000	6.696000000
C	2.569000000	44.073000000	5.803000000
C	2.827000000	45.400000000	6.090000000
C	3.739000000	45.429000000	7.206000000
C	4.028000000	44.106000000	7.551000000
C	1.624000000	43.492000000	4.832000000
H	4.562000000	30.964000000	9.311000000
H	5.081000000	30.475000000	10.934000000
H	1.982000000	33.382000000	10.869000000
H	2.828000000	32.944000000	9.359000000
H	4.181000000	33.835000000	11.944000000
H	4.332000000	34.629000000	10.368000000
H	5.759000000	32.921000000	9.536000000
H	6.094000000	32.627000000	11.253000000
H	0.710000000	29.644000000	13.841000000
H	2.036000000	30.557000000	14.555000000
H	0.068000000	29.040000000	10.209000000
H	-0.059000000	28.807000000	11.952000000
H	3.593000000	28.679000000	14.255000000
H	2.423000000	27.778000000	13.301000000
H	0.880000000	27.659000000	15.279000000
H	2.073000000	28.493000000	16.261000000
H	2.511000000	27.950000000	9.314000000
H	0.053000000	26.495000000	12.516000000
H	2.220000000	26.010000000	16.605000000
H	3.708000000	26.605000000	15.852000000
H	2.474000000	25.783000000	14.867000000
H	3.430000000	25.672000000	9.001000000
H	0.939000000	24.217000000	12.171000000
H	2.002000000	23.582000000	8.226000000
H	4.166000000	20.598000000	11.507000000
H	2.694000000	21.324000000	7.530000000
H	3.723000000	19.779000000	9.204000000
Ca	2.205000000	34.508000000	7.497000000
O	4.488000000	33.683000000	7.447000000
C	4.140000000	33.363000000	6.272000000
O	3.493000000	34.144000000	5.500000000
C	4.151000000	31.887000000	5.895000000
H	4.583000000	31.726000000	4.903000000
H	4.714000000	31.311000000	6.634000000
C	2.657000000	31.483000000	5.885000000
H	2.200000000	31.941000000	5.003000000
O	1.964000000	32.084000000	7.003000000
H	1.967000000	31.456000000	7.766000000
C	2.418000000	29.978000000	5.862000000
H	1.341000000	29.799000000	5.963000000
H	2.907000000	29.518000000	6.728000000
C	2.902000000	29.293000000	4.579000000
H	3.989000000	29.440000000	4.473000000
O	2.227000000	29.800000000	3.424000000
H	2.568000000	30.682000000	3.228000000
C	2.622000000	27.794000000	4.623000000
H	3.108000000	27.370000000	5.506000000
H	1.545000000	27.638000000	4.747000000
C	3.094000000	27.079000000	3.356000000
H	2.602000000	27.524000000	2.492000000
H	4.171000000	27.215000000	3.218000000
N	2.791000000	25.650000000	3.360000000
C	1.830000000	25.061000000	2.541000000
C	1.883000000	23.696000000	2.752000000
C	2.894000000	23.452000000	3.749000000
C	3.452000000	24.686000000	4.097000000
C	0.902000000	25.835000000	1.698000000
C	0.030000000	26.784000000	2.258000000
H	0.043000000	26.949000000	3.329000000
C	-0.861000000	27.501000000	1.467000000
H	-1.532000000	28.237000000	1.894000000
C	-0.889000000	27.250000000	0.101000000
C	-0.054000000	26.311000000	-0.491000000
H	-0.104000000	26.148000000	-1.561000000
C	0.843000000	25.612000000	0.313000000
H	1.515000000	24.891000000	-0.136000000

C	5.011000000	27.183000000	-2.614000000
C	6.707000000	30.928000000	-6.007000000
C	5.112000000	26.606000000	-1.345000000
C	5.488000000	26.468000000	-3.717000000
C	6.019000000	31.698000000	-7.135000000
C	5.605000000	25.318000000	-1.189000000
C	5.992000000	25.178000000	-3.562000000
C	6.015000000	24.568000000	-2.301000000
C	6.418000000	23.153000000	-2.107000000
C	5.911000000	22.093000000	-2.896000000
C	7.278000000	22.835000000	-1.046000000
C	6.318000000	20.775000000	-2.631000000
C	7.641000000	21.521000000	-0.766000000
C	4.831000000	22.254000000	-3.883000000
C	7.170000000	20.485000000	-1.571000000
H	2.221000000	23.251000000	-5.082000000
H	3.589000000	31.963000000	0.683000000
H	4.265000000	32.874000000	-0.677000000
H	6.823000000	29.900000000	0.938000000
H	5.303000000	30.229000000	1.797000000
H	7.474000000	32.246000000	1.048000000
H	6.786000000	32.005000000	2.654000000
H	4.861000000	33.329000000	2.086000000
H	5.867000000	33.917000000	0.766000000
H	6.810000000	29.248000000	-3.804000000
H	7.692000000	30.719000000	-3.382000000
H	4.317000000	28.824000000	-3.828000000
H	3.259000000	28.403000000	-2.476000000
H	5.914000000	32.125000000	-4.384000000
H	4.949000000	30.714000000	-4.772000000
H	6.773000000	29.864000000	-6.271000000
H	7.741000000	31.278000000	-5.895000000
H	4.746000000	27.151000000	-0.485000000
H	5.442000000	26.912000000	-4.708000000
H	6.555000000	31.582000000	-8.082000000
H	5.971000000	32.769000000	-6.908000000
H	4.993000000	31.345000000	-7.286000000
H	5.614000000	24.859000000	-0.208000000
H	6.342000000	24.629000000	-4.429000000
H	5.910000000	19.977000000	-3.241000000
H	8.262000000	21.309000000	0.095000000
H	7.427000000	19.456000000	-1.345000000
Ca	2.355000000	34.162000000	6.245000000
O	4.596000000	33.309000000	6.389000000
C	4.374000000	33.010000000	5.176000000
O	3.772000000	33.789000000	4.367000000
C	4.467000000	31.550000000	4.774000000
H	4.840000000	31.433000000	3.757000000
H	5.104000000	30.993000000	5.468000000
C	3.005000000	31.048000000	4.832000000
H	2.460000000	31.485000000	3.996000000
O	2.329000000	31.581000000	6.013000000
H	2.804000000	31.250000000	6.790000000
C	2.872000000	29.531000000	4.774000000
H	1.818000000	29.267000000	4.914000000
H	3.449000000	29.067000000	5.584000000
C	3.344000000	28.989000000	3.423000000
H	4.409000000	29.255000000	3.304000000
O	2.554000000	29.640000000	2.443000000
H	2.928000000	29.527000000	1.546000000
C	3.254000000	27.460000000	3.328000000
H	3.768000000	27.007000000	4.183000000
H	2.205000000	27.153000000	3.382000000
C	3.884000000	26.978000000	2.018000000
H	3.361000000	27.427000000	1.176000000
H	4.922000000	27.319000000	1.961000000
C	0.513000000	42.747000000	5.265000000
H	0.349000000	42.610000000	6.328000000
C	-0.393000000	42.205000000	4.358000000
H	-1.254000000	41.632000000	4.684000000
C	-0.191000000	42.430000000	3.001000000
C	0.887000000	43.171000000	2.532000000
H	1.008000000	43.325000000	1.466000000
C	1.793000000	43.691000000	3.453000000
H	2.649000000	44.255000000	3.102000000
F	-1.066000000	41.914000000	2.115000000
C	2.192000000	46.567000000	5.433000000
C	2.579000000	46.978000000	4.149000000
H	3.343000000	46.417000000	3.620000000
C	2.015000000	48.112000000	3.561000000
H	2.327000000	48.416000000	2.565000000
C	1.059000000	48.855000000	4.251000000
H	0.623000000	49.739000000	3.797000000
C	0.669000000	48.458000000	5.533000000
H	-0.070000000	49.038000000	6.079000000
C	1.230000000	47.327000000	6.119000000
H	0.939000000	47.025000000	7.120000000
C	4.270000000	46.630000000	7.893000000
O	4.633000000	46.623000000	9.075000000
N	4.336000000	47.756000000	7.107000000
H	4.011000000	47.652000000	6.157000000
C	4.679000000	49.070000000	7.465000000
C	4.489000000	50.060000000	6.486000000
H	4.069000000	49.783000000	5.522000000
C	4.827000000	51.384000000	6.750000000
H	4.677000000	52.135000000	5.981000000
C	5.350000000	51.742000000	7.993000000
H	5.615000000	52.774000000	8.199000000
C	5.528000000	50.759000000	8.965000000
H	5.932000000	51.026000000	9.937000000
C	5.201000000	49.426000000	8.717000000
H	5.338000000	48.663000000	9.468000000
C	4.966000000	43.520000000	8.579000000
H	5.055000000	42.451000000	8.357000000
C	6.387000000	44.102000000	8.482000000
H	7.052000000	43.561000000	9.161000000
H	6.395000000	45.157000000	8.756000000
H	6.793000000	43.994000000	7.472000000
C	4.402000000	43.646000000	10.009000000
H	3.411000000	43.187000000	10.080000000
H	4.323000000	44.698000000	10.283000000
H	5.066000000	43.142000000	10.719000000

Complex **III**

O	3.446000000	29.326000000	-0.144000000
N	6.253000000	31.234000000	-1.288000000
N	4.852000000	29.582000000	-1.958000000
N	3.841000000	23.152000000	-3.819000000
N	4.641000000	21.379000000	-4.918000000
N	3.083000000	22.780000000	-4.843000000
N	3.530000000	21.724000000	-5.517000000
C	5.291000000	30.999000000	-0.202000000
C	4.520000000	32.277000000	0.201000000
C	6.038000000	30.644000000	1.100000000
C	6.554000000	32.006000000	1.585000000
C	5.437000000	33.016000000	1.211000000
C	4.390000000	29.884000000	-0.706000000
C	5.973000000	30.408000000	-2.227000000
C	6.693000000	30.304000000	-3.533000000
C	4.304000000	28.508000000	-2.783000000
C	5.983000000	31.068000000	-4.666000000

H	2.455000000	39.583000000	5.366000000
O	4.146000000	39.139000000	6.496000000
H	4.051000000	38.184000000	6.361000000
C	2.967000000	41.187000000	6.694000000
H	3.283000000	41.325000000	7.734000000
H	1.987000000	41.663000000	6.579000000
C	3.968000000	41.873000000	5.761000000
H	4.964000000	41.463000000	5.908000000
H	3.694000000	41.680000000	4.723000000
N	3.992000000	43.322000000	5.964000000
C	3.023000000	44.168000000	5.431000000
C	3.168000000	45.409000000	6.025000000
C	4.265000000	45.309000000	6.954000000
C	4.743000000	43.997000000	6.908000000
C	2.007000000	43.729000000	4.460000000
C	0.643000000	43.891000000	4.756000000
H	0.361000000	44.328000000	5.708000000
C	-0.341000000	43.511000000	3.848000000
H	-1.396000000	43.633000000	4.067000000
C	0.048000000	42.954000000	2.636000000
C	1.386000000	42.783000000	2.302000000
H	1.648000000	42.360000000	1.338000000
C	2.359000000	43.182000000	3.215000000
H	3.408000000	43.089000000	2.952000000
F	-0.900000000	42.574000000	1.755000000
C	2.317000000	46.584000000	5.749000000
C	2.109000000	47.031000000	4.434000000
H	2.575000000	46.493000000	3.615000000
C	1.322000000	48.154000000	4.179000000
H	1.173000000	48.485000000	3.156000000
C	0.736000000	48.855000000	5.234000000
H	0.130000000	49.734000000	5.036000000
C	0.936000000	48.421000000	6.547000000
H	0.486000000	48.961000000	7.374000000
C	1.713000000	47.294000000	6.801000000
H	1.870000000	46.960000000	7.822000000
C	4.828000000	46.411000000	7.771000000
O	5.336000000	46.248000000	8.882000000
N	4.741000000	47.649000000	7.170000000
H	4.301000000	47.659000000	6.262000000
C	4.902000000	48.918000000	7.750000000
C	4.356000000	50.010000000	7.055000000
H	3.822000000	49.837000000	6.124000000
C	4.480000000	51.298000000	7.563000000
H	4.052000000	52.131000000	7.014000000
C	5.141000000	51.518000000	8.773000000
H	5.235000000	52.523000000	9.172000000
C	5.677000000	50.431000000	9.463000000
H	6.194000000	50.589000000	10.405000000
C	5.570000000	49.133000000	8.965000000
H	5.982000000	48.288000000	9.496000000
C	5.864000000	43.325000000	7.663000000
H	6.011000000	42.338000000	7.215000000
C	7.201000000	44.075000000	7.521000000
H	8.002000000	43.492000000	7.987000000
H	7.157000000	45.047000000	8.013000000
H	7.457000000	44.224000000	6.467000000
C	5.498000000	43.104000000	9.144000000
H	4.580000000	42.516000000	9.241000000
H	5.350000000	44.067000000	9.634000000
H	6.302000000	42.560000000	9.652000000

Complex **IV**

O	2.869000000	18.306000000	-3.316000000
N	4.584000000	19.690000000	-6.126000000
N	3.863000000	25.532000000	1.813000000
C	2.973000000	24.875000000	0.963000000
C	3.338000000	23.540000000	0.926000000
C	4.513000000	23.389000000	1.751000000
C	4.806000000	24.648000000	2.294000000
C	2.016000000	25.584000000	0.096000000
C	1.092000000	26.519000000	0.586000000
H	1.016000000	26.696000000	1.653000000
C	0.265000000	27.239000000	-0.274000000
H	-0.437000000	27.975000000	0.100000000
C	0.355000000	26.998000000	-1.638000000
C	1.240000000	26.061000000	-2.161000000
H	1.284000000	25.905000000	-3.233000000
C	2.071000000	25.361000000	-1.293000000
H	2.785000000	24.654000000	-1.698000000
F	-0.437000000	27.692000000	-2.482000000
C	2.604000000	22.522000000	0.141000000
C	3.230000000	21.821000000	-0.899000000
H	4.272000000	22.016000000	-1.107000000
C	2.529000000	20.883000000	-1.653000000
H	3.035000000	20.359000000	-2.461000000
C	1.185000000	20.623000000	-1.372000000
H	0.637000000	19.890000000	-1.955000000
C	0.548000000	21.312000000	-0.335000000
H	-0.496000000	21.112000000	-0.105000000
C	1.253000000	22.252000000	0.416000000
H	0.766000000	22.786000000	1.224000000
C	5.355000000	22.183000000	1.921000000
O	6.546000000	22.241000000	2.246000000
N	4.729000000	20.983000000	1.666000000
H	3.764000000	21.044000000	1.370000000
C	5.350000000	19.753000000	1.381000000
C	4.644000000	18.847000000	0.573000000
H	3.662000000	19.118000000	0.198000000
C	5.213000000	17.626000000	0.226000000
H	4.657000000	16.940000000	-0.407000000
C	6.489000000	17.286000000	0.680000000
H	6.933000000	16.334000000	0.409000000
C	7.181000000	18.182000000	1.497000000
H	8.168000000	17.924000000	1.870000000
C	6.623000000	19.408000000	1.858000000
H	7.158000000	20.106000000	2.484000000
C	5.906000000	25.126000000	3.213000000
H	5.623000000	26.129000000	3.546000000
C	7.246000000	25.264000000	2.463000000
H	8.009000000	25.685000000	3.126000000
H	7.145000000	25.926000000	1.597000000
H	7.574000000	24.282000000	2.120000000
C	6.039000000	24.275000000	4.488000000
H	6.777000000	24.728000000	5.158000000
H	6.367000000	23.264000000	4.249000000
H	5.084000000	24.221000000	5.020000000
O	1.289000000	34.728000000	8.315000000
C	0.557000000	35.445000000	7.563000000
O	0.177000000	35.084000000	6.405000000
C	0.456000000	36.927000000	7.889000000
H	-0.527000000	37.338000000	7.650000000
H	0.688000000	37.110000000	8.942000000
C	1.509000000	37.582000000	6.969000000
H	1.139000000	37.541000000	5.941000000
O	2.721000000	36.750000000	6.913000000
H	3.034000000	36.647000000	7.827000000
C	1.856000000	39.014000000	7.354000000
H	2.276000000	39.036000000	8.369000000
H	0.918000000	39.583000000	7.384000000
C	2.840000000	39.698000000	6.396000000

H	0.687000000	28.729000000	2.133000000
O	0.145000000	28.031000000	4.012000000
H	0.150000000	28.904000000	4.442000000
C	1.278000000	26.677000000	2.470000000
H	0.277000000	26.269000000	2.294000000
H	1.714000000	26.101000000	3.296000000
C	2.114000000	26.455000000	1.206000000
H	3.139000000	26.836000000	1.359000000
O	1.534000000	27.074000000	0.055000000
H	1.537000000	28.029000000	0.188000000
C	2.180000000	24.960000000	0.911000000
H	2.581000000	24.451000000	1.790000000
H	1.156000000	24.596000000	0.770000000
C	3.001000000	24.572000000	-0.321000000
H	2.619000000	25.090000000	-1.200000000
H	4.051000000	24.852000000	-0.203000000
N	2.904000000	23.132000000	-0.567000000
C	1.923000000	22.581000000	-1.383000000
C	1.921000000	21.205000000	-1.194000000
C	2.948000000	20.923000000	-0.214000000
C	3.523000000	22.136000000	0.168000000
C	1.070000000	23.444000000	-2.232000000
C	-0.165000000	23.920000000	-1.770000000
H	-0.508000000	23.630000000	-0.782000000
C	-0.956000000	24.752000000	-2.559000000
H	-1.908000000	25.135000000	-2.210000000
C	-0.502000000	25.096000000	-3.827000000
C	0.714000000	24.642000000	-4.324000000
H	1.026000000	24.924000000	-5.323000000
C	1.498000000	23.822000000	-3.516000000
H	2.449000000	23.451000000	-3.885000000
F	-1.270000000	25.892000000	-4.600000000
C	0.979000000	20.262000000	-1.825000000
C	0.549000000	20.440000000	-3.152000000
H	0.923000000	21.277000000	-3.726000000
C	-0.337000000	19.547000000	-3.748000000
H	-0.652000000	19.711000000	-4.774000000
C	-0.804000000	18.439000000	-3.039000000
H	-1.491000000	17.739000000	-3.506000000
C	-0.389000000	18.249000000	-1.720000000
H	-0.749000000	17.398000000	-1.152000000
C	0.480000000	19.155000000	-1.117000000
H	0.775000000	19.002000000	-0.086000000
C	3.331000000	19.613000000	0.383000000
O	3.579000000	19.509000000	1.589000000
N	3.403000000	18.549000000	-0.479000000
H	3.237000000	18.718000000	-1.470000000
C	3.580000000	17.194000000	-0.128000000
C	3.827000000	16.279000000	-1.166000000
H	3.885000000	16.636000000	-2.186000000
C	3.982000000	14.924000000	-0.890000000
H	4.180000000	14.240000000	-1.708000000
C	3.889000000	14.449000000	0.419000000
H	4.009000000	13.392000000	0.633000000
C	3.644000000	15.357000000	1.448000000
H	3.569000000	15.007000000	2.474000000
C	3.493000000	16.719000000	1.192000000
H	3.323000000	17.418000000	1.996000000
C	4.653000000	22.453000000	1.123000000
H	4.931000000	23.498000000	0.945000000
C	5.912000000	21.613000000	0.840000000
H	6.730000000	21.943000000	1.489000000
H	6.236000000	21.723000000	-0.199000000
H	5.729000000	20.557000000	1.043000000
C	4.245000000	22.333000000	2.606000000
H	5.067000000	22.675000000	3.245000000
N	2.854000000	18.310000000	-5.625000000
N	1.138000000	11.542000000	-6.891000000
N	2.421000000	10.300000000	-8.240000000
N	0.587000000	11.306000000	-8.076000000
N	1.323000000	10.567000000	-8.902000000
C	4.460000000	19.655000000	-4.659000000
C	4.195000000	21.083000000	-4.080000000
C	5.775000000	19.195000000	-3.983000000
C	6.535000000	20.500000000	-3.729000000
C	5.431000000	21.438000000	-3.223000000
C	3.314000000	18.694000000	-4.388000000
C	3.670000000	18.926000000	-6.604000000
C	3.411000000	18.721000000	-8.061000000
C	1.783000000	17.323000000	-5.788000000
C	2.180000000	19.496000000	-8.570000000
C	2.255000000	15.896000000	-5.622000000
C	1.861000000	19.183000000	-10.033000000
C	2.221000000	15.284000000	-4.365000000
C	2.741000000	15.166000000	-6.712000000
C	0.632000000	19.936000000	-10.547000000
C	2.659000000	13.971000000	-4.208000000
C	3.193000000	13.861000000	-6.553000000
C	3.152000000	13.240000000	-5.297000000
C	3.670000000	11.860000000	-5.113000000
C	3.326000000	10.788000000	-5.965000000
C	4.592000000	11.618000000	-4.084000000
C	3.941000000	9.539000000	-5.803000000
C	5.180000000	10.367000000	-3.913000000
C	2.303000000	10.901000000	-7.016000000
C	4.865000000	9.325000000	-4.785000000
H	-0.328000000	11.652000000	-8.331000000
H	3.283000000	21.100000000	-3.483000000
H	4.072000000	21.766000000	-4.925000000
H	6.308000000	18.470000000	-4.604000000
H	5.521000000	18.716000000	-3.030000000
H	6.949000000	20.872000000	-4.673000000
H	7.360000000	20.379000000	-3.020000000
H	5.212000000	21.224000000	-2.172000000
H	5.698000000	22.498000000	-3.293000000
H	3.286000000	17.652000000	-8.268000000
H	4.305000000	19.054000000	-8.594000000
H	1.338000000	17.470000000	-6.774000000
H	1.030000000	17.562000000	-5.034000000
H	2.365000000	20.569000000	-8.443000000
H	1.304000000	19.264000000	-7.951000000
H	1.702000000	18.101000000	-10.142000000
H	2.733000000	19.425000000	-10.654000000
H	1.854000000	15.843000000	-3.509000000
H	2.764000000	15.618000000	-7.700000000
H	0.422000000	19.691000000	-11.593000000
H	0.776000000	21.019000000	-10.479000000
H	-0.258000000	19.683000000	-9.960000000
H	2.599000000	13.501000000	-3.232000000
H	3.584000000	13.317000000	-7.407000000
H	4.874000000	12.440000000	-3.434000000
H	3.669000000	8.736000000	-6.480000000
H	5.895000000	10.213000000	-3.111000000
H	5.328000000	8.351000000	-4.668000000
Ca	2.053000000	32.506000000	5.301000000
O	1.189000000	30.421000000	4.697000000
C	2.246000000	30.148000000	4.014000000
O	3.164000000	31.011000000	3.859000000
C	2.406000000	28.754000000	3.438000000
H	3.153000000	28.798000000	2.641000000
H	2.820000000	28.128000000	4.240000000
C	1.088000000	28.112000000	2.961000000

H	4.878000000	48.777000000	9.812000000
C	4.911000000	43.592000000	8.719000000
H	5.093000000	42.546000000	8.452000000
C	6.288000000	44.268000000	8.828000000
H	6.904000000	43.734000000	9.559000000
H	6.189000000	45.303000000	9.156000000
H	6.811000000	44.250000000	7.866000000
C	4.169000000	43.594000000	10.069000000
H	3.216000000	43.060000000	9.993000000
H	3.971000000	44.622000000	10.379000000
H	4.777000000	43.099000000	10.834000000

H	4.010000000	21.295000000	2.837000000
H	3.364000000	22.939000000	2.836000000
O	3.029000000	34.343000000	6.399000000
C	1.867000000	34.754000000	6.779000000
O	0.815000000	34.113000000	6.481000000
C	1.782000000	36.044000000	7.569000000
H	0.740000000	36.370000000	7.593000000
H	2.107000000	35.846000000	8.598000000
C	2.701000000	37.122000000	6.970000000
H	2.468000000	37.205000000	5.893000000
O	4.069000000	36.752000000	7.133000000
H	4.140000000	35.847000000	6.779000000
C	2.506000000	38.503000000	7.590000000
H	2.784000000	38.477000000	8.651000000
H	1.444000000	38.768000000	7.523000000
C	3.346000000	39.566000000	6.869000000
H	3.144000000	39.458000000	5.785000000
O	4.733000000	39.405000000	7.125000000
H	4.916000000	38.453000000	7.047000000
C	2.982000000	40.997000000	7.256000000
H	3.178000000	41.150000000	8.323000000
H	1.914000000	41.164000000	7.084000000
C	3.799000000	41.977000000	6.412000000
H	4.862000000	41.799000000	6.577000000
H	3.604000000	41.786000000	5.354000000
N	3.501000000	43.389000000	6.644000000
C	2.764000000	44.164000000	5.754000000
C	2.848000000	45.480000000	6.168000000
C	3.659000000	45.499000000	7.361000000
C	4.051000000	44.185000000	7.628000000
C	1.943000000	43.559000000	4.686000000
C	0.804000000	42.807000000	5.015000000
H	0.533000000	42.693000000	6.059000000
C	0.006000000	42.231000000	4.030000000
H	-0.877000000	41.653000000	4.275000000
C	0.353000000	42.427000000	2.698000000
C	1.466000000	43.170000000	2.329000000
H	1.700000000	43.299000000	1.278000000
C	2.260000000	43.728000000	3.330000000
H	3.144000000	44.293000000	3.060000000
F	-0.417000000	41.877000000	1.736000000
C	2.145000000	46.628000000	5.555000000
C	2.449000000	47.056000000	4.254000000
H	3.201000000	46.521000000	3.684000000
C	1.821000000	48.174000000	3.703000000
H	2.072000000	48.491000000	2.694000000
C	0.885000000	48.889000000	4.448000000
H	0.402000000	49.764000000	4.025000000
C	0.577000000	48.477000000	5.747000000
H	-0.148000000	49.031000000	6.336000000
C	1.199000000	47.358000000	6.294000000
H	0.969000000	47.043000000	7.306000000
C	4.061000000	46.699000000	8.132000000
O	4.312000000	46.686000000	9.341000000
N	4.158000000	47.840000000	7.365000000
H	3.933000000	47.729000000	6.387000000
C	4.409000000	49.164000000	7.753000000
C	4.282000000	50.147000000	6.757000000
H	3.970000000	49.855000000	5.756000000
C	4.545000000	51.481000000	7.047000000
H	4.451000000	52.227000000	6.263000000
C	4.926000000	51.859000000	8.336000000
H	5.132000000	52.900000000	8.562000000
C	5.038000000	50.882000000	9.325000000
H	5.330000000	51.165000000	10.332000000
C	4.788000000	49.538000000	9.050000000

IRB (**dimer**; tetrazole 1*H* tautomer)

N	1.412750000	6.780990000	-2.731230000
C	1.341210000	6.182440000	-3.968800000
N	2.134230000	6.927040000	-4.899350000
C	2.990320000	7.847780000	-4.127350000
C	2.287370000	7.864530000	-2.757050000
C	3.068460000	9.265200000	-4.704820000
H	3.066560000	9.253440000	-5.800880000
H	2.245220000	9.911410000	-4.381730000
C	4.412140000	9.791950000	-4.214990000
H	4.778320000	10.620490000	-4.828250000
H	4.333860000	10.145300000	-3.180640000
C	5.322590000	8.576360000	-4.289670000
H	6.207520000	8.683870000	-3.655330000
H	5.667670000	8.431950000	-5.319900000
C	4.447940000	7.397540000	-3.860670000
H	4.620880000	7.183190000	-2.798280000
H	4.737330000	6.487440000	-4.394900000
C	0.841490000	6.328980000	-1.470270000
H	1.585770000	6.511580000	-0.683970000
H	0.695320000	5.244250000	-1.516070000
C	-0.447580000	7.002100000	-1.029560000
C	-1.197920000	6.405300000	0.000030000
H	-0.877480000	5.455850000	0.432010000
C	-2.328930000	7.035630000	0.537230000
H	-2.869860000	6.550220000	1.350820000
C	-2.749200000	8.280340000	0.050630000
C	-1.980900000	8.894220000	-0.950970000
H	-2.241900000	9.891630000	-1.308140000
C	-0.847310000	8.265510000	-1.480780000
H	-0.254670000	8.814880000	-2.208900000
C	-3.993540000	8.907020000	0.521300000
C	-5.193980000	8.917430000	-0.231700000
C	-6.313900000	9.633050000	0.216450000
H	-7.240940000	9.663470000	-0.354930000
C	-6.265360000	10.310520000	1.433690000
H	-7.135900000	10.859480000	1.790710000
C	-5.103590000	10.285270000	2.199890000
H	-5.068750000	10.817760000	3.149540000
C	-3.977430000	9.595460000	1.746320000
H	-3.072720000	9.620680000	2.354890000
C	-5.323840000	8.167110000	-1.526100000
N	-4.469380000	7.207490000	-2.005190000
N	-4.903210000	6.870990000	-3.167500000
N	-6.052340000	7.551090000	-3.517050000
N	-6.328730000	8.332000000	-2.460200000
O	2.509460000	8.633800000	-1.837490000
N	-4.135400000	4.822780000	-5.963790000
C	-4.303850000	6.057400000	-5.391570000
N	-4.194110000	5.942790000	-3.967840000
C	-4.361150000	4.515160000	-3.638530000
C	-4.074490000	3.834380000	-4.987840000
C	-3.363130000	4.024000000	-2.579670000
H	-3.126060000	4.809670000	-1.856460000
H	-2.416880000	3.671320000	-3.002130000
C	-4.099840000	2.904620000	-1.856360000
H	-3.682660000	2.709150000	-0.864350000
H	-4.055490000	1.975620000	-2.436080000
C	-5.526370000	3.421210000	-1.789650000
H	-6.248200000	2.626100000	-1.582010000
H	-5.615160000	4.175310000	-0.998200000
C	-5.759860000	4.071350000	-3.151380000
H	-6.210650000	3.342610000	-3.836630000
H	-6.482070000	4.886730000	-3.055360000
C	-4.002420000	4.503240000	-7.372600000

B97D3 functional

IRB (tetrazole 1*H* tautomer)

O	-1.895458000	0.290135000	-7.820055000
N	-1.512529000	2.917354000	-5.389650000
N	-2.849611000	1.135036000	-5.863189000
N	-0.218619000	-0.938411000	-1.867808000
N	0.601247000	-1.325579000	0.097609000
N	0.566877000	0.121193000	-1.559804000
N	1.051134000	-0.132338000	-0.372477000
C	-0.999255000	2.295817000	-6.626005000
C	0.467734000	1.842504000	-6.407548000
C	-0.898911000	3.316094000	-7.795214000
C	0.575743000	3.819538000	-7.806039000
C	1.256642000	3.146232000	-6.592558000
C	-1.927749000	1.115037000	-6.912249000
C	-2.519981000	2.211889000	-4.999011000
C	-3.239055000	2.423594000	-3.699448000
C	-3.874345000	0.105405000	-5.710582000
C	-2.514950000	1.703028000	-2.539213000
C	-3.472059000	-0.933953000	-4.682639000
C	-3.268001000	1.800181000	-1.207133000
C	-2.290795000	-1.674461000	-4.877403000
C	-4.202095000	-1.119487000	-3.501141000
C	-2.596940000	0.990051000	-0.090300000
C	-1.827277000	-2.540623000	-3.882393000
C	-3.732005000	-1.975399000	-2.496818000
C	-2.522242000	-2.670788000	-2.660808000
C	-1.989872000	-3.522622000	-1.557379000
C	-0.901863000	-3.124340000	-0.735262000
C	-2.619991000	-4.753076000	-1.301100000
C	-0.476492000	-3.980167000	0.305115000
C	-2.187101000	-5.592608000	-0.270322000
C	-0.202240000	-1.836143000	-0.846785000
C	-1.108130000	-5.202177000	0.533818000
H	0.716428000	1.111351000	-7.191241000
H	0.603417000	1.371119000	-5.425832000
H	-1.624685000	4.122071000	-7.639352000
H	-1.151819000	2.804270000	-8.732390000
H	0.637686000	4.913700000	-7.758115000
H	1.067629000	3.508156000	-8.738060000
H	2.330768000	2.975738000	-6.742133000
H	1.131005000	3.766150000	-5.695477000
H	-4.278541000	2.072215000	-3.767840000
H	-3.265711000	3.502994000	-3.504335000
H	-3.970753000	-0.349563000	-6.705888000
H	-4.832551000	0.569635000	-5.446569000
H	-1.502551000	2.117761000	-2.442002000
H	-2.395619000	0.644691000	-2.801334000
H	-4.301825000	1.442956000	-1.350320000
H	-3.348366000	2.857959000	-0.909924000
H	-1.736152000	-1.556915000	-5.807827000
H	-5.120682000	-0.554494000	-3.340608000
H	-3.126863000	1.108666000	0.864176000
H	-1.555075000	1.301220000	0.058215000
H	-2.588742000	-0.080467000	-0.337621000
H	-0.910794000	-3.110822000	-4.036047000
H	-4.281645000	-2.078360000	-1.562205000
H	-3.457597000	-5.045903000	-1.933214000
H	0.350853000	-3.653901000	0.931029000
H	-2.688734000	-6.544129000	-0.097387000
H	-0.760725000	-5.846621000	1.340265000
H	-0.723210000	-0.972738000	-2.747505000

C	-2.406411000	-2.097291000	-0.789055000
C	-4.536503000	-0.996174000	-0.963299000
C	-4.204940000	-1.492253000	-2.387098000
C	-2.869177000	-2.286145000	-2.254828000
C	-3.174886000	-0.746955000	1.223983000
C	-2.240305000	1.120067000	0.314272000
C	-1.697565000	2.525089000	0.297866000
C	-1.921914000	0.698777000	2.826993000
C	-0.429971000	2.709597000	-0.553079000
C	-0.474232000	0.277649000	2.647434000
C	0.201251000	4.097336000	-0.398867000
C	-0.171236000	-1.087264000	2.484189000
C	0.529509000	1.225450000	2.399460000
C	1.484309000	4.231343000	-1.229310000
C	1.060123000	-1.480135000	1.957434000
C	1.759586000	0.832752000	1.859935000
C	2.009054000	-0.518262000	1.569972000
C	3.205220000	-0.895394000	0.760657000
C	3.183899000	-0.776980000	-0.656039000
C	4.383117000	-1.327140000	1.389917000
C	4.353187000	-1.062521000	-1.389424000
C	5.533878000	-1.623074000	0.649410000
C	1.982923000	-0.361327000	-1.401448000
C	5.519303000	-1.480540000	-0.743851000
H	-0.993211000	0.152829000	-1.901732000
H	-2.752502000	-2.926244000	-0.156884000
H	-1.322409000	-2.007608000	-0.662620000
H	-5.113824000	-0.063974000	-0.945111000
H	-5.086550000	-1.756204000	-0.390360000
H	-4.065882000	-0.630307000	-3.051418000
H	-5.018304000	-2.099454000	-2.804153000
H	-2.996395000	-3.351131000	-2.487257000
H	-2.118372000	-1.897895000	-2.954758000
H	-1.505117000	2.854652000	1.324628000
H	-2.498989000	3.175213000	-0.086861000
H	-2.460915000	0.059762000	3.535594000
H	-2.018001000	1.741448000	3.149548000
H	-0.670479000	2.532988000	-1.609144000
H	0.303554000	1.949137000	-0.261278000
H	0.427601000	4.277864000	0.665544000
H	-0.523118000	4.873841000	-0.694002000
H	-0.935014000	-1.833617000	2.698040000
H	0.331121000	2.284353000	2.566281000
H	1.941606000	5.222485000	-1.109165000
H	1.279738000	4.077427000	-2.297665000
H	2.224576000	3.475808000	-0.929923000
H	1.259889000	-2.533266000	1.763467000
H	2.505744000	1.582592000	1.598277000
H	4.390786000	-1.414438000	2.476089000
H	4.325235000	-0.954839000	-2.471970000
H	6.437897000	-1.955208000	1.159296000
H	6.411835000	-1.701086000	-1.328470000

IRB (**dimer**; tetrazole **2*H*** tautomer)

N	-0.289020000	3.005512000	1.327308000
C	0.547500000	2.131158000	0.627810000
N	1.204504000	2.701937000	-0.337868000
C	0.838384000	4.131629000	-0.366298000
C	-0.182128000	4.283458000	0.763177000
C	0.263898000	4.546131000	-1.764989000
H	0.402428000	3.694277000	-2.441619000
H	-0.808633000	4.762774000	-1.710719000
C	1.104295000	5.757047000	-2.223185000
H	1.158753000	5.834330000	-3.316087000
H	-4.458680000	3.520410000	-7.550640000
H	-4.584520000	5.208490000	-7.975290000
C	-2.558800000	4.446550000	-7.833310000
C	-2.005070000	5.471960000	-8.608550000
H	-2.598640000	6.344970000	-8.877130000
C	-0.714740000	5.349090000	-9.137950000
H	-0.350530000	6.125600000	-9.812140000
C	0.051500000	4.196550000	-8.906490000
C	-0.487220000	3.189740000	-8.097020000
H	0.055450000	2.256720000	-7.938550000
C	-1.774230000	3.314930000	-7.566350000
H	-2.189060000	2.476220000	-7.005590000
C	1.317560000	3.985290000	-9.616220000
C	2.546860000	4.579170000	-9.280420000
C	3.675480000	4.378240000	-10.100470000
H	4.639050000	4.832900000	-9.869150000
C	3.600720000	3.569490000	-11.232950000
H	4.479610000	3.413930000	-11.856840000
C	2.400250000	2.958530000	-11.563380000
H	2.334330000	2.327070000	-12.448050000
C	1.274040000	3.168390000	-10.767550000
H	0.338330000	2.694550000	-11.069610000
C	2.754770000	5.429200000	-8.060170000
N	1.931040000	5.619470000	-6.965250000
N	2.579060000	6.405910000	-6.155570000
N	3.781240000	6.816770000	-6.711040000
N	3.901240000	6.164460000	-7.856760000
C	-4.387570000	7.343400000	-6.159950000
H	-4.205010000	7.179300000	-7.225490000
H	-5.416070000	7.718000000	-6.100960000
C	-3.431400000	8.453860000	-5.696530000
H	-3.635450000	9.347310000	-6.300360000
H	-3.641680000	8.734960000	-4.658390000
C	-1.952700000	8.087600000	-5.836090000
H	-1.708380000	7.227440000	-5.203870000
H	-1.735710000	7.806920000	-6.871530000
C	-1.057710000	9.255110000	-5.450820000
H	-0.005510000	8.976860000	-5.555890000
H	-1.243470000	10.120450000	-6.094970000
H	-1.230360000	9.557690000	-4.413840000
O	-3.856320000	2.646230000	-5.154720000
C	0.539070000	4.947790000	-4.210150000
H	-0.272770000	4.878710000	-3.477290000
H	0.023360000	5.006720000	-5.171960000
C	1.403500000	3.687670000	-4.133660000
H	1.956720000	3.670340000	-3.185940000
H	2.151030000	3.696860000	-4.935880000
C	0.554290000	2.421590000	-4.240510000
H	-0.024230000	2.435500000	-5.169280000
H	-0.163300000	2.384370000	-3.412800000
C	1.416000000	1.169710000	-4.209370000
H	1.985370000	1.108420000	-3.276420000
H	0.788870000	0.275880000	-4.284230000
H	2.122970000	1.159540000	-5.045150000
H	1.002240000	5.247860000	-6.839670000
H	-3.622660000	6.850040000	-1.587770000

IRB (tetrazole **2*H*** tautomer)

O	-3.545246000	-1.581455000	2.038311000
N	-2.506242000	0.422236000	-0.747418000
N	-2.610567000	0.504840000	1.530001000
N	0.711159000	-0.535650000	-0.993707000
N	2.031387000	0.249669000	-2.632894000
N	0.025355000	-0.026116000	-2.013686000
N	0.782810000	0.468775000	-2.999044000
C	-3.150862000	-0.831566000	-0.299099000

H	2.918029000	-4.136739000	0.918708000
C	6.464027000	-1.126560000	0.400256000
C	6.646598000	0.259231000	0.154082000
C	7.845785000	0.885813000	0.541241000
H	7.969002000	1.946438000	0.330315000
C	8.847385000	0.168721000	1.202676000
H	9.768213000	0.669612000	1.499590000
C	8.653408000	-1.188485000	1.490549000
H	9.424317000	-1.754609000	2.013092000
C	7.475820000	-1.824977000	1.086618000
H	7.344451000	-2.892011000	1.264719000
C	5.593895000	1.088429000	-0.456355000
N	4.285160000	0.997393000	-0.146711000
N	3.758872000	1.942210000	-0.921940000
H	2.729381000	2.207929000	-0.841361000
N	4.651765000	2.607558000	-1.659165000
N	5.829745000	2.073280000	-1.377521000
C	0.150487000	-1.903463000	-2.867793000
H	1.155024000	-1.500458000	-2.665440000
H	0.268004000	-2.581517000	-3.729792000
C	-0.826440000	-0.762895000	-3.210458000
H	-0.528670000	-0.343828000	-4.182845000
H	-1.834593000	-1.178564000	-3.351496000
C	-0.861590000	0.359146000	-2.159553000
H	-1.083528000	-0.076144000	-1.176814000
H	0.137814000	0.816100000	-2.086309000
C	-1.905751000	1.439504000	-2.463315000
H	-1.889040000	2.221942000	-1.693327000
H	-1.720789000	1.916193000	-3.437400000
H	-2.916655000	1.013932000	-2.470280000
O	0.443793000	-5.077622000	0.755752000
C	0.725377000	0.706679000	1.046558000
H	-0.244404000	0.278884000	1.334554000
H	1.088568000	0.145544000	0.178038000
C	1.752876000	0.571418000	2.194260000
H	1.443534000	1.178291000	3.059730000
H	2.709380000	0.974853000	1.838034000
C	1.949122000	-0.888925000	2.620171000
H	2.134243000	-1.498679000	1.724208000
H	1.020588000	-1.269572000	3.075862000
C	3.118729000	-1.059220000	3.596725000
H	2.967356000	-0.465204000	4.509254000
H	3.237759000	-2.109546000	3.896494000
H	4.057563000	-0.729267000	3.132198000

ATR calcium (ATR-Ca-ATR)

Ca	2.331737000	34.572630000	8.698514000
O	3.009541000	32.279819000	8.382522000
C	3.149504000	32.481683000	7.094693000
O	3.185827000	33.673173000	6.600722000
C	3.103757000	31.249952000	6.199605000
H	3.542015000	31.472154000	5.223360000
H	3.677349000	30.451170000	6.685285000
C	1.608313000	30.791762000	6.040523000
H	1.159536000	31.325188000	5.187909000
O	0.777223000	31.203148000	7.176164000
H	1.322213000	31.092678000	8.004935000
C	1.486639000	29.269228000	5.847218000
H	0.417802000	29.013554000	5.833875000
H	1.935141000	28.778022000	6.727123000
C	2.135727000	28.701075000	4.572214000
H	3.196724000	29.012686000	4.513458000
O	1.424532000	29.161007000	3.363359000
H	1.551156000	30.130014000	3.251068000
H	0.654598000	6.685881000	-1.842668000
C	2.469278000	5.530417000	-1.555348000
H	3.097058000	6.430790000	-1.530901000
H	3.035795000	4.745250000	-2.075945000
C	2.077684000	5.056413000	-0.146950000
H	1.750317000	5.912913000	0.459110000
H	2.874691000	4.532617000	0.394388000
C	-1.226924000	2.688220000	2.409852000
H	-1.342049000	3.624928000	2.971213000
H	-0.764282000	1.948109000	3.071135000
C	-2.575644000	2.206494000	1.910597000
C	-3.074495000	0.942065000	2.261124000
H	-2.479253000	0.282723000	2.895732000
C	-4.337327000	0.526763000	1.825179000
H	-4.716197000	-0.453932000	2.106592000
C	-5.126557000	1.367298000	1.018841000
C	-4.621917000	2.632692000	0.671562000
H	-5.226488000	3.290413000	0.047601000
C	-3.363723000	3.051572000	1.110093000
H	-2.993770000	4.041747000	0.849455000
C	-6.500955000	0.980613000	0.589142000
C	-6.791332000	-0.149215000	-0.220039000
C	-8.135075000	-0.446706000	-0.532923000
H	-8.337423000	-1.318058000	-1.152805000
C	-9.179044000	0.358334000	-0.074818000
H	-10.208411000	0.108137000	-0.331277000
C	-8.895409000	1.489252000	0.699876000
H	-9.700358000	2.130551000	1.057998000
C	-7.568636000	1.789181000	1.023810000
H	-7.340957000	2.654276000	1.645966000
C	-5.755428000	-1.023529000	-0.788190000
N	-4.439518000	-0.742141000	-0.864551000
N	-3.961995000	-1.827965000	-1.465986000
H	-2.921637000	-2.038065000	-1.474092000
N	-4.886803000	-2.754910000	-1.741262000
N	-6.035843000	-2.254659000	-1.329491000
O	-0.791511000	5.280273000	1.126725000
N	0.594114000	-3.670941000	-1.104416000
C	-0.260503000	-2.718676000	-1.675631000
N	-1.394095000	-2.610109000	-1.059240000
C	-1.383569000	-3.506647000	0.108951000
C	-0.029127000	-4.217675000	0.025457000
C	-1.529387000	-2.671096000	1.427776000
H	-1.968643000	-1.704003000	1.148720000
H	-0.558747000	-2.483841000	1.901515000
C	-2.509679000	-3.466099000	2.304819000
H	-2.989807000	-2.838287000	3.067013000
H	-1.975668000	-4.278526000	2.819436000
C	-3.494288000	-4.049514000	1.279457000
H	-4.111619000	-4.863680000	1.678113000
H	-4.173134000	-3.265185000	0.917126000
C	-2.573096000	-4.522469000	0.138890000
H	-2.162916000	-5.513592000	0.379318000
H	-3.073125000	-4.578260000	-0.832824000
C	1.893451000	-4.140637000	-1.599813000
H	1.965665000	-5.184100000	-1.266666000
H	1.866865000	-4.132601000	-2.695473000
C	3.070658000	-3.343868000	-1.083047000
C	3.792149000	-2.487289000	-1.930569000
H	3.510002000	-2.402121000	-2.980321000
C	4.883107000	-1.760996000	-1.448173000
H	5.439466000	-1.108232000	-2.118436000
C	5.281049000	-1.873046000	-0.102078000
C	4.565727000	-2.745697000	0.738917000
H	4.860868000	-2.840281000	1.782468000
C	3.471434000	-3.470310000	0.258270000

O	2.835337000	37.364095000	9.033002000
H	3.369550000	37.364791000	9.845416000
C	1.989872000	39.684757000	9.509555000
H	2.666026000	39.785421000	10.375360000
H	1.070931000	40.243905000	9.756360000
C	2.660498000	40.320562000	8.277438000
H	2.015006000	40.147805000	7.391031000
O	3.983822000	39.734316000	8.042444000
H	3.866891000	38.752902000	8.026575000
C	2.875292000	41.826671000	8.445182000
H	3.470275000	42.004957000	9.352234000
H	1.901349000	42.315687000	8.595945000
C	3.576716000	42.438249000	7.216108000
H	4.641167000	42.180586000	7.226499000
H	3.153833000	42.013561000	6.298597000
N	3.423714000	43.901679000	7.115795000
C	2.667984000	44.542889000	6.107633000
C	2.920756000	45.915556000	6.210058000
C	3.819802000	46.112229000	7.331521000
C	4.129330000	44.847185000	7.864660000
C	1.707017000	43.838520000	5.243751000
C	0.744509000	42.953239000	5.791064000
H	0.703683000	42.799412000	6.862063000
C	-0.193739000	42.298945000	4.968926000
H	-0.934503000	41.625584000	5.384213000
C	-0.170258000	42.560370000	3.598401000
C	0.746241000	43.438558000	3.018338000
H	0.730463000	43.615752000	1.949025000
C	1.690474000	44.068423000	3.849880000
H	2.420247000	44.738609000	3.411170000
F	-1.103906000	41.917808000	2.776218000
C	2.277094000	46.987055000	5.409666000
C	2.774037000	47.346474000	4.135214000
H	3.621740000	46.796392000	3.723529000
C	2.201631000	48.405201000	3.411002000
H	2.596803000	48.670906000	2.432378000
C	1.120977000	49.123238000	3.952579000
H	0.678184000	49.946641000	3.395957000
C	0.618565000	48.774661000	5.219207000
H	-0.215896000	49.330493000	5.644065000
C	1.193327000	47.719919000	5.940389000
H	0.817080000	47.456390000	6.928754000
C	4.271571000	47.415309000	7.852568000
O	4.496811000	47.627465000	9.100359000
N	4.440788000	48.409237000	6.908045000
H	4.271237000	48.126307000	5.939078000
C	4.789701000	49.762684000	7.065101000
C	5.093983000	50.493582000	5.894439000
H	5.054813000	49.995409000	4.929423000
C	5.450522000	51.849392000	5.972620000
H	5.692517000	52.391096000	5.063885000
C	5.497785000	52.494475000	7.223452000
H	5.776874000	53.546133000	7.283830000
C	5.187458000	51.776697000	8.381904000
H	5.214854000	52.264219000	9.354732000
C	4.833682000	50.408343000	8.329051000
H	4.611468000	49.850979000	9.217306000
C	5.059586000	44.431934000	8.985981000
H	5.229801000	43.348944000	8.886366000
C	6.449744000	45.109314000	8.892271000
H	7.121081000	44.676318000	9.648948000
H	6.370226000	46.185262000	9.079961000
H	6.897871000	44.951540000	7.900870000
C	4.412245000	44.680122000	10.376383000
H	3.447084000	44.159100000	10.462671000
H	4.245713000	45.754703000	10.508951000
C	2.057676000	27.171077000	4.541068000
H	2.533833000	26.779122000	5.449158000
H	1.000345000	26.871229000	4.579177000
C	2.720274000	26.572473000	3.286715000
H	2.308578000	27.054987000	2.396639000
H	3.802904000	26.767423000	3.298111000
N	2.501105000	25.119728000	3.150977000
C	1.720318000	24.525342000	2.132962000
C	1.879934000	23.142375000	2.245204000
C	2.750423000	22.888070000	3.374716000
C	3.135336000	24.132288000	3.912394000
C	0.841055000	25.283962000	1.226448000
C	-0.042623000	26.279629000	1.705316000
H	-0.074214000	26.514760000	2.764657000
C	-0.901576000	26.970869000	0.837550000
H	-1.573060000	27.740097000	1.205503000
C	-0.885476000	26.638129000	-0.518829000
C	-0.040792000	25.656234000	-1.036559000
H	-0.054117000	25.429100000	-2.098586000
C	0.829391000	24.987879000	-0.159705000
H	1.505185000	24.237127000	-0.550079000
F	-1.747036000	27.317729000	-1.391053000
C	1.160524000	22.109078000	1.451960000
C	1.663723000	21.656181000	0.210998000
H	2.571769000	22.104039000	-0.189460000
C	1.010150000	20.631757000	-0.500765000
H	1.409769000	20.292362000	-1.452170000
C	-0.158357000	20.049253000	0.022790000
H	-0.663147000	19.255823000	-0.522668000
C	-0.667680000	20.495051000	1.255284000
H	-1.570292000	20.046063000	1.664748000
C	-0.011784000	21.513434000	1.964932000
H	-0.395244000	21.851460000	2.924876000
C	3.126172000	21.560687000	3.916742000
O	3.344307000	21.344639000	5.139724000
N	3.231115000	20.553734000	2.960399000
H	3.072863000	20.832303000	1.997364000
C	3.538881000	19.191066000	3.129686000
C	3.875943000	18.446020000	1.976522000
H	3.889917000	18.951001000	1.012401000
C	4.195946000	17.093045000	2.078886000
H	4.470975000	16.539123000	1.168919000
C	4.179398000	16.443340000	3.314122000
H	4.433664000	15.385970000	3.394411000
C	3.836716000	17.159329000	4.469375000
H	3.809642000	16.681289000	5.437848000
C	3.509823000	18.554318000	4.410242000
H	3.271515000	19.101935000	5.271470000
C	4.092433000	24.483767000	5.032158000
H	4.319879000	25.558107000	4.942531000
C	5.446531000	23.738975000	4.915301000
H	6.146407000	24.126187000	5.670565000
H	5.892161000	23.887154000	3.920753000
H	5.313781000	22.665936000	5.089382000
C	3.453685000	24.254892000	6.429633000
H	4.149018000	24.578418000	7.218803000
H	3.234387000	23.188656000	6.552786000
H	2.516283000	24.820374000	6.537197000
O	1.952648000	35.509999000	10.933372000
C	0.939503000	36.128689000	10.363716000
O	0.262637000	35.593758000	9.392699000
C	0.838833000	37.625285000	10.549840000
H	-0.202107000	37.971380000	10.523940000
H	1.309541000	37.941219000	11.490315000
C	1.591784000	38.219520000	9.326801000
H	0.995136000	38.068102000	8.421620000

O	3.767000000	33.606000000	7.634000000
C	3.470000000	33.323000000	6.427000000
O	2.784000000	34.087000000	5.662000000
C	3.600000000	31.866000000	5.987000000
H	3.962000000	31.789000000	4.953000000
H	4.277000000	31.311000000	6.650000000
C	2.155000000	31.310000000	6.071000000
H	1.565000000	31.764000000	5.269000000
O	1.485000000	31.788000000	7.287000000
H	1.970000000	31.404000000	8.037000000
C	2.065000000	29.788000000	5.984000000
H	1.011000000	29.492000000	6.079000000
H	2.619000000	29.339000000	6.826000000
C	2.629000000	29.228000000	4.665000000
H	3.710000000	29.454000000	4.617000000
O	1.959000000	29.804000000	3.529000000
H	2.386000000	30.647000000	3.321000000
C	2.449000000	27.711000000	4.563000000
H	2.781000000	27.238000000	5.495000000
H	1.378000000	27.487000000	4.459000000
C	3.223000000	27.145000000	3.363000000
H	2.958000000	27.714000000	2.468000000
H	4.303000000	27.264000000	3.519000000
N	2.942000000	25.735000000	3.088000000
C	2.186000000	25.284000000	1.998000000
C	2.253000000	23.893000000	1.995000000
C	3.063000000	23.489000000	3.125000000
C	3.489000000	24.663000000	3.777000000
C	1.527000000	26.170000000	1.025000000
C	0.696000000	27.241000000	1.420000000
H	0.520000000	27.432000000	2.475000000
C	0.075000000	28.067000000	0.480000000
H	-0.557000000	28.895000000	0.788000000
C	0.270000000	27.800000000	-0.875000000
C	1.060000000	26.735000000	-1.312000000
H	1.187000000	26.557000000	-2.377000000
C	1.693000000	25.933000000	-0.359000000
H	2.325000000	25.110000000	-0.684000000
F	-0.321000000	28.605000000	-1.800000000
C	1.612000000	23.038000000	0.971000000
C	2.385000000	22.167000000	0.173000000
H	3.461000000	22.128000000	0.328000000
C	1.780000000	21.357000000	-0.793000000
H	2.392000000	20.686000000	-1.396000000
C	0.391000000	21.410000000	-0.986000000
H	-0.080000000	20.779000000	-1.740000000
C	-0.388000000	22.276000000	-0.206000000
H	-1.468000000	22.325000000	-0.351000000
C	0.217000000	23.081000000	0.767000000
H	-0.384000000	23.754000000	1.377000000
C	3.346000000	22.104000000	3.587000000
O	4.273000000	21.813000000	4.355000000
N	2.462000000	21.151000000	3.109000000
H	1.733000000	21.491000000	2.486000000
C	2.547000000	19.748000000	3.184000000
C	1.716000000	19.012000000	2.311000000
H	1.066000000	19.542000000	1.613000000
C	1.740000000	17.615000000	2.327000000
H	1.094000000	17.062000000	1.644000000
C	2.587000000	16.931000000	3.210000000
H	2.607000000	15.842000000	3.222000000
C	3.407000000	17.666000000	4.076000000
H	4.067000000	17.146000000	4.771000000
C	3.396000000	19.065000000	4.077000000
H	4.028000000	19.636000000	4.744000000
C	4.383000000	24.888000000	4.977000000
H	5.078621000	44.313758000	11.172224000

Complex **V**

O	-3.394000000	27.294000000	-1.192000000
N	-3.627000000	30.663000000	-2.439000000
N	-2.999000000	28.577000000	-3.104000000
N	-7.474000000	29.265000000	-5.865000000
N	-8.663000000	30.264000000	-7.492000000
N	-7.843000000	30.509000000	-5.567000000
N	-8.554000000	31.135000000	-6.516000000
C	-3.823000000	29.753000000	-1.295000000
C	-5.297000000	29.778000000	-0.802000000
C	-3.034000000	30.249000000	-0.065000000
C	-3.912000000	31.390000000	0.471000000
C	-5.361000000	30.862000000	0.314000000
C	-3.391000000	28.368000000	-1.783000000
C	-3.172000000	29.945000000	-3.414000000
C	-2.888000000	30.479000000	-4.787000000
C	-2.579000000	27.489000000	-3.984000000
C	-4.170000000	30.612000000	-5.640000000
C	-3.635000000	27.212000000	-5.035000000
C	-3.865000000	31.029000000	-7.084000000
C	-4.943000000	26.902000000	-4.621000000
C	-3.376000000	27.364000000	-6.403000000
C	-5.116000000	31.082000000	-7.968000000
C	-5.979000000	26.814000000	-5.549000000
C	-4.416000000	27.272000000	-7.338000000
C	-5.737000000	27.036000000	-6.918000000
C	-6.839000000	26.993000000	-7.920000000
C	-7.896000000	27.944000000	-7.975000000
C	-6.802000000	25.975000000	-8.893000000
C	-8.875000000	27.829000000	-8.987000000
C	-7.779000000	25.870000000	-9.887000000
C	-7.998000000	29.121000000	-7.096000000
C	-8.825000000	26.802000000	-9.930000000
H	-7.576000000	30.956000000	-4.698000000
H	-5.542000000	28.784000000	-0.404000000
H	-5.969000000	29.980000000	-1.645000000
H	-2.017000000	30.555000000	-0.339000000
H	-2.974000000	29.419000000	0.654000000
H	-3.765000000	32.275000000	-0.162000000
H	-3.668000000	31.668000000	1.505000000
H	-5.713000000	30.413000000	1.253000000
H	-6.059000000	31.671000000	0.062000000
H	-2.168000000	29.832000000	-5.305000000
H	-2.422000000	31.466000000	-4.671000000
H	-2.438000000	26.629000000	-3.315000000
H	-1.609000000	27.729000000	-4.439000000
H	-4.826000000	31.355000000	-5.160000000
H	-4.716000000	29.661000000	-5.639000000
H	-3.147000000	30.312000000	-7.515000000
H	-3.359000000	32.008000000	-7.083000000
H	-5.146000000	26.763000000	-3.559000000
H	-2.366000000	27.598000000	-6.740000000
H	-4.877000000	31.431000000	-8.982000000
H	-5.874000000	31.761000000	-7.554000000
H	-5.571000000	30.088000000	-8.055000000
H	-6.993000000	26.604000000	-5.216000000
H	-4.216000000	27.431000000	-8.397000000
H	-5.987000000	25.254000000	-8.849000000
H	-9.666000000	28.573000000	-9.027000000
H	-7.726000000	25.066000000	-10.620000000
H	-9.595000000	26.733000000	-10.698000000
Ca	1.496000000	34.437000000	7.651000000

C	4.753000000	51.546000000	6.620000000
H	4.534000000	52.242000000	5.810000000
C	5.222000000	52.022000000	7.853000000
H	5.376000000	53.090000000	8.008000000
C	5.489000000	51.109000000	8.881000000
H	5.854000000	51.467000000	9.844000000
C	5.302000000	49.734000000	8.699000000
H	5.505000000	49.023000000	9.491000000
C	4.986000000	43.731000000	8.945000000
H	5.048000000	42.645000000	8.787000000
C	6.434000000	44.263000000	8.945000000
H	7.019000000	43.747000000	9.719000000
H	6.452000000	45.336000000	9.160000000
H	6.917000000	44.087000000	7.975000000
C	4.298000000	43.957000000	10.310000000
H	3.285000000	43.531000000	10.318000000
H	4.228000000	45.030000000	10.512000000
H	4.880000000	43.474000000	11.109000000

Complex **VI**

O	1.744000000	30.135000000	9.194000000
N	3.525000000	30.860000000	12.239000000
N	1.882000000	29.505000000	11.424000000
N	3.923000000	23.462000000	12.982000000
N	3.505000000	21.300000000	13.455000000
N	4.214000000	23.122000000	14.238000000
N	3.975000000	21.843000000	14.556000000
C	3.399000000	31.125000000	10.795000000
C	4.736000000	30.822000000	10.049000000
C	3.196000000	32.628000000	10.520000000
C	4.604000000	33.208000000	10.709000000
C	5.528000000	32.163000000	10.039000000
C	2.257000000	30.237000000	10.321000000
C	2.662000000	29.948000000	12.534000000
C	2.437000000	29.394000000	13.906000000
C	0.845000000	28.471000000	11.384000000
C	3.062000000	28.002000000	14.133000000
C	1.382000000	27.067000000	11.161000000
C	2.677000000	27.423000000	15.500000000
C	2.345000000	26.815000000	10.170000000
C	0.880000000	25.984000000	11.903000000
C	3.206000000	25.998000000	15.699000000
C	2.772000000	25.511000000	9.915000000
C	1.306000000	24.677000000	11.643000000
C	2.249000000	24.417000000	10.631000000
C	2.599000000	23.029000000	10.222000000
C	3.084000000	22.027000000	11.103000000
C	2.383000000	22.681000000	8.874000000
C	3.281000000	20.715000000	10.624000000
C	2.604000000	21.384000000	8.401000000
C	3.473000000	22.289000000	12.496000000
C	3.040000000	20.392000000	9.287000000
H	4.564000000	23.793000000	14.911000000
H	4.490000000	30.503000000	9.027000000
H	5.263000000	29.998000000	10.545000000
H	2.416000000	33.077000000	11.146000000
H	2.911000000	32.710000000	9.463000000
H	4.819000000	33.286000000	11.782000000
H	4.706000000	34.203000000	10.259000000
H	5.728000000	32.463000000	9.004000000
H	6.487000000	32.070000000	10.564000000
H	1.355000000	29.346000000	14.106000000
H	2.864000000	30.118000000	14.611000000
H	0.184000000	28.756000000	10.554000000
H	0.261000000	28.519000000	12.310000000
H	4.405000000	25.970000000	5.158000000
C	5.842000000	24.466000000	4.699000000
H	6.475000000	24.719000000	5.562000000
H	6.237000000	24.991000000	3.817000000
H	5.892000000	23.388000000	4.521000000
C	3.820000000	24.244000000	6.262000000
H	4.453000000	24.512000000	7.120000000
H	3.803000000	23.155000000	6.171000000
H	2.800000000	24.600000000	6.462000000
O	0.528000000	35.030000000	9.784000000
C	-0.175000000	35.813000000	9.060000000
O	-0.632000000	35.493000000	7.908000000
C	-0.151000000	37.304000000	9.399000000
H	-1.120000000	37.786000000	9.222000000
H	0.156000000	37.465000000	10.440000000
C	0.886000000	37.901000000	8.418000000
H	0.463000000	37.860000000	7.406000000
O	2.060000000	37.006000000	8.312000000
H	2.427000000	36.928000000	9.211000000
C	1.323000000	39.325000000	8.752000000
H	1.793000000	39.354000000	9.748000000
H	0.415000000	39.945000000	8.806000000
C	2.301000000	39.926000000	7.721000000
H	1.862000000	39.782000000	6.711000000
O	3.587000000	39.308000000	7.794000000
H	3.429000000	38.355000000	7.668000000
C	2.509000000	41.425000000	7.934000000
H	2.868000000	41.606000000	8.956000000
H	1.545000000	41.940000000	7.827000000
C	3.503000000	42.003000000	6.916000000
H	4.510000000	41.623000000	7.106000000
H	3.223000000	41.685000000	5.906000000
N	3.519000000	43.468000000	6.906000000
C	2.841000000	44.224000000	5.947000000
C	3.058000000	45.565000000	6.235000000
C	3.896000000	45.619000000	7.415000000
C	4.161000000	44.296000000	7.812000000
C	1.970000000	43.598000000	4.927000000
C	0.658000000	43.200000000	5.258000000
H	0.290000000	43.380000000	6.267000000
C	-0.178000000	42.601000000	4.311000000
H	-1.193000000	42.294000000	4.555000000
C	0.310000000	42.411000000	3.015000000
C	1.600000000	42.797000000	2.645000000
H	1.941000000	42.633000000	1.625000000
C	2.425000000	43.385000000	3.611000000
H	3.440000000	43.680000000	3.346000000
F	-0.494000000	41.835000000	2.090000000
C	2.452000000	46.703000000	5.509000000
C	2.672000000	46.888000000	4.129000000
H	3.289000000	46.170000000	3.591000000
C	2.128000000	47.991000000	3.457000000
H	2.314000000	48.119000000	2.391000000
C	1.363000000	48.933000000	4.156000000
H	0.950000000	49.797000000	3.636000000
C	1.139000000	48.763000000	5.531000000
H	0.548000000	49.493000000	6.084000000
C	1.673000000	47.658000000	6.199000000
H	1.507000000	47.528000000	7.268000000
C	4.427000000	46.847000000	8.064000000
O	4.694000000	46.934000000	9.270000000
N	4.612000000	47.901000000	7.179000000
H	4.340000000	47.714000000	6.222000000
C	4.828000000	49.262000000	7.459000000
C	4.553000000	50.179000000	6.421000000
H	4.166000000	49.812000000	5.470000000

C	3.415000000	22.258000000	4.420000000
O	3.872000000	22.204000000	5.575000000
N	3.295000000	21.135000000	3.621000000
H	2.910000000	21.294000000	2.697000000
C	3.429000000	19.779000000	3.967000000
C	3.038000000	18.835000000	2.992000000
H	2.645000000	19.184000000	2.036000000
C	3.142000000	17.468000000	3.253000000
H	2.843000000	16.754000000	2.486000000
C	3.624000000	17.017000000	4.490000000
H	3.702000000	15.950000000	4.695000000
C	4.006000000	17.956000000	5.457000000
H	4.387000000	17.619000000	6.422000000
C	3.923000000	19.331000000	5.209000000
H	4.237000000	20.059000000	5.948000000
C	4.497000000	25.288000000	5.029000000
H	4.652000000	26.354000000	4.811000000
C	5.838000000	24.582000000	4.735000000
H	6.637000000	25.036000000	5.337000000
H	6.108000000	24.679000000	3.675000000
H	5.781000000	23.520000000	4.990000000
C	4.127000000	25.177000000	6.524000000
H	4.911000000	25.642000000	7.139000000
H	4.028000000	24.124000000	6.801000000
H	3.175000000	25.682000000	6.740000000
O	0.749000000	35.141000000	9.756000000
C	0.030000000	35.900000000	9.023000000
O	-0.428000000	35.556000000	7.880000000
C	0.025000000	37.396000000	9.342000000
H	-0.953000000	37.856000000	9.160000000
H	0.330000000	37.577000000	10.381000000
C	1.049000000	38.000000000	8.354000000
H	0.630000000	37.928000000	7.343000000
O	2.247000000	37.137000000	8.270000000
H	2.590000000	37.059000000	9.177000000
C	1.441000000	39.443000000	8.662000000
H	1.920000000	39.504000000	9.652000000
H	0.512000000	40.031000000	8.717000000
C	2.384000000	40.063000000	7.611000000
H	1.937000000	39.891000000	6.609000000
O	3.692000000	39.491000000	7.673000000
H	3.563000000	38.529000000	7.595000000
C	2.540000000	41.571000000	7.807000000
H	2.924000000	41.769000000	8.816000000
H	1.552000000	42.046000000	7.734000000
C	3.473000000	42.188000000	6.756000000
H	4.497000000	41.827000000	6.897000000
H	3.158000000	41.877000000	5.754000000
N	3.458000000	43.653000000	6.767000000
C	2.797000000	44.416000000	5.800000000
C	3.018000000	45.757000000	6.098000000
C	3.835000000	45.802000000	7.293000000
C	4.096000000	44.475000000	7.682000000
C	1.931000000	43.807000000	4.770000000
C	0.685000000	43.246000000	5.122000000
H	0.364000000	43.283000000	6.162000000
C	-0.151000000	42.671000000	4.161000000
H	-1.115000000	42.240000000	4.425000000
C	0.265000000	42.673000000	2.827000000
C	1.487000000	43.223000000	2.435000000
H	1.776000000	43.203000000	1.387000000
C	2.316000000	43.781000000	3.415000000
H	3.283000000	44.192000000	3.131000000
F	-0.542000000	42.125000000	1.888000000
C	2.420000000	46.908000000	5.385000000
C	2.746000000	47.189000000	4.043000000
H	4.156000000	28.087000000	14.048000000
H	2.742000000	27.312000000	13.342000000
H	1.578000000	27.414000000	15.589000000
H	3.045000000	28.082000000	16.302000000
H	2.748000000	27.639000000	9.584000000
H	0.138000000	26.163000000	12.683000000
H	2.920000000	25.591000000	16.678000000
H	4.306000000	25.986000000	15.635000000
H	2.807000000	25.337000000	14.918000000
H	3.520000000	25.326000000	9.147000000
H	0.888000000	23.846000000	12.211000000
H	2.013000000	23.444000000	8.192000000
H	3.651000000	19.963000000	11.318000000
H	2.452000000	21.163000000	7.348000000
H	3.209000000	19.376000000	8.933000000
Ca	1.704000000	34.497000000	7.617000000
O	4.034000000	33.760000000	7.422000000
C	3.611000000	33.421000000	6.267000000
O	2.874000000	34.174000000	5.540000000
C	3.656000000	31.942000000	5.884000000
H	4.001000000	31.807000000	4.850000000
H	4.311000000	31.382000000	6.563000000
C	2.184000000	31.465000000	6.002000000
H	1.621000000	31.937000000	5.187000000
O	1.569000000	31.993000000	7.212000000
H	1.711000000	31.344000000	7.948000000
C	2.014000000	29.948000000	5.934000000
H	0.952000000	29.709000000	6.082000000
H	2.573000000	29.482000000	6.759000000
C	2.480000000	29.331000000	4.603000000
H	3.564000000	29.520000000	4.479000000
O	1.760000000	29.863000000	3.479000000
H	2.081000000	30.762000000	3.318000000
C	2.254000000	27.820000000	4.594000000
H	2.722000000	27.391000000	5.488000000
H	1.177000000	27.620000000	4.669000000
C	2.816000000	27.153000000	3.332000000
H	2.298000000	27.538000000	2.451000000
H	3.882000000	27.395000000	3.216000000
N	2.659000000	25.696000000	3.341000000
C	1.776000000	25.003000000	2.505000000
C	1.944000000	23.645000000	2.755000000
C	2.950000000	23.515000000	3.787000000
C	3.387000000	24.810000000	4.120000000
C	0.801000000	25.678000000	1.628000000
C	-0.203000000	26.514000000	2.161000000
H	-0.259000000	26.661000000	3.238000000
C	-1.140000000	27.140000000	1.336000000
H	-1.914000000	27.788000000	1.742000000
C	-1.080000000	26.909000000	-0.040000000
C	-0.109000000	26.082000000	-0.607000000
H	-0.090000000	25.932000000	-1.685000000
C	0.832000000	25.478000000	0.233000000
H	1.611000000	24.853000000	-0.196000000
F	-1.989000000	27.506000000	-0.848000000
C	1.148000000	22.545000000	2.158000000
C	1.368000000	22.120000000	0.832000000
H	2.119000000	22.631000000	0.231000000
C	0.659000000	21.035000000	0.299000000
H	0.843000000	20.720000000	-0.729000000
C	-0.274000000	20.351000000	1.088000000
H	-0.820000000	19.502000000	0.677000000
C	-0.497000000	20.759000000	2.411000000
H	-1.217000000	20.227000000	3.032000000
C	0.205000000	21.846000000	2.940000000
H	0.044000000	22.158000000	3.971000000

C	7.677000000	21.419000000	-0.704000000
C	4.942000000	21.921000000	-3.965000000
C	7.270000000	20.335000000	-1.488000000
H	2.553000000	22.909000000	-5.590000000
H	3.816000000	31.929000000	0.514000000
H	4.489000000	32.765000000	-0.903000000
H	6.955000000	29.720000000	0.755000000
H	5.462000000	30.138000000	1.633000000
H	7.730000000	32.032000000	0.817000000
H	7.020000000	31.875000000	2.433000000
H	5.190000000	33.317000000	1.814000000
H	6.211000000	33.765000000	0.443000000
H	6.818000000	28.926000000	-3.971000000
H	7.787000000	30.363000000	-3.601000000
H	4.297000000	28.634000000	-3.946000000
H	3.253000000	28.281000000	-2.553000000
H	6.079000000	31.841000000	-4.624000000
H	5.015000000	30.478000000	-4.949000000
H	6.750000000	29.463000000	-6.461000000
H	7.825000000	30.823000000	-6.144000000
H	4.591000000	26.966000000	-0.587000000
H	5.489000000	26.709000000	-4.785000000
H	6.611000000	31.142000000	-8.325000000
H	6.138000000	32.408000000	-7.174000000
H	5.049000000	31.043000000	-7.487000000
H	5.433000000	24.657000000	-0.259000000
H	6.367000000	24.413000000	-4.456000000
H	7.566000000	23.551000000	-0.418000000
H	6.050000000	19.709000000	-3.153000000
H	8.319000000	21.263000000	0.160000000
H	7.579000000	19.323000000	-1.236000000
Ca	2.725000000	34.206000000	6.301000000
O	4.989000000	33.368000000	6.141000000
C	4.607000000	33.075000000	4.959000000
O	3.894000000	33.855000000	4.234000000
C	4.666000000	31.612000000	4.534000000
H	4.897000000	31.507000000	3.470000000
H	5.403000000	31.057000000	5.131000000
C	3.232000000	31.075000000	4.774000000
H	2.575000000	31.505000000	4.013000000
O	2.686000000	31.587000000	6.041000000
H	3.258000000	31.241000000	6.746000000
C	3.129000000	29.550000000	4.734000000
H	2.091000000	29.263000000	4.954000000
H	3.775000000	29.108000000	5.509000000
C	3.526000000	28.988000000	3.362000000
H	4.592000000	29.238000000	3.187000000
O	2.694000000	29.636000000	2.406000000
H	3.050000000	29.517000000	1.498000000
C	3.407000000	27.454000000	3.305000000
H	3.983000000	27.009000000	4.130000000
H	2.357000000	27.165000000	3.447000000
C	3.918000000	26.931000000	1.958000000
H	3.351000000	27.400000000	1.152000000
H	4.968000000	27.220000000	1.816000000
N	3.801000000	25.484000000	1.765000000
C	2.869000000	24.896000000	0.898000000
C	3.152000000	23.536000000	0.847000000
C	4.297000000	23.289000000	1.701000000
C	4.681000000	24.530000000	2.253000000
C	1.873000000	25.683000000	0.150000000
C	0.971000000	26.553000000	0.799000000
H	0.963000000	26.603000000	1.885000000
C	0.085000000	27.355000000	0.076000000
H	-0.598000000	28.039000000	0.574000000
C	0.086000000	27.264000000	-1.317000000
H	3.441000000	46.534000000	3.519000000
C	2.214000000	48.312000000	3.395000000
H	2.481000000	48.513000000	2.357000000
C	1.354000000	49.179000000	4.081000000
H	0.948000000	50.056000000	3.580000000
C	1.025000000	48.914000000	5.420000000
H	0.361000000	49.586000000	5.962000000
C	1.550000000	47.790000000	6.063000000
H	1.306000000	47.589000000	7.106000000
C	4.324000000	47.026000000	7.979000000
O	4.563000000	47.090000000	9.193000000
N	4.496000000	48.107000000	7.126000000
H	4.267000000	47.932000000	6.155000000
C	4.681000000	49.465000000	7.434000000
C	4.504000000	50.387000000	6.380000000
H	4.217000000	50.023000000	5.392000000
C	4.677000000	51.754000000	6.603000000
H	4.538000000	52.452000000	5.776000000
C	5.020000000	52.227000000	7.877000000
H	5.154000000	53.294000000	8.052000000
C	5.187000000	51.309000000	8.923000000
H	5.452000000	51.664000000	9.919000000
C	5.027000000	49.934000000	8.717000000
H	5.155000000	49.220000000	9.523000000
C	4.932000000	43.902000000	8.802000000
H	5.020000000	42.822000000	8.618000000
C	6.368000000	44.467000000	8.811000000
H	6.968000000	43.943000000	9.568000000
H	6.364000000	45.533000000	9.056000000
H	6.848000000	44.330000000	7.833000000
C	4.246000000	44.080000000	10.176000000
H	3.242000000	43.636000000	10.177000000
H	4.156000000	45.147000000	10.404000000
H	4.841000000	43.590000000	10.959000000

Complex **VII**

O	3.508000000	29.281000000	-0.249000000
N	6.407000000	31.019000000	-1.497000000
N	4.907000000	29.412000000	-2.101000000
N	4.005000000	22.877000000	-4.128000000
N	4.812000000	20.931000000	-4.917000000
N	3.343000000	22.418000000	-5.189000000
N	3.798000000	21.257000000	-5.685000000
C	5.449000000	30.858000000	-0.388000000
C	4.748000000	32.187000000	-0.009000000
C	6.205000000	30.505000000	0.914000000
C	6.792000000	31.857000000	1.359000000
C	5.731000000	32.918000000	0.947000000
C	4.478000000	29.772000000	-0.841000000
C	6.067000000	30.174000000	-2.411000000
C	6.761000000	29.997000000	-3.726000000
C	4.296000000	28.339000000	-2.892000000
C	6.075000000	30.770000000	-4.875000000
C	4.963000000	26.991000000	-2.705000000
C	6.763000000	30.541000000	-6.227000000
C	4.995000000	26.412000000	-1.427000000
C	5.484000000	26.265000000	-3.788000000
C	6.105000000	31.326000000	-7.369000000
C	5.472000000	25.115000000	-1.244000000
C	5.974000000	24.967000000	-3.605000000
C	5.936000000	24.356000000	-2.337000000
C	6.368000000	22.953000000	-2.103000000
C	5.934000000	21.841000000	-2.879000000
C	7.233000000	22.706000000	-1.019000000
C	6.407000000	20.550000000	-2.563000000

C	3.109000000	45.667000000	6.129000000
C	4.069000000	45.657000000	7.215000000
C	4.567000000	44.347000000	7.333000000
C	2.188000000	43.805000000	4.565000000
C	0.888000000	43.367000000	4.886000000
H	0.535000000	43.470000000	5.910000000
C	0.049000000	42.814000000	3.913000000
H	-0.956000000	42.471000000	4.149000000
C	0.525000000	42.710000000	2.604000000
C	1.808000000	43.136000000	2.243000000
H	2.140000000	43.041000000	1.210000000
C	2.634000000	43.681000000	3.233000000
H	3.640000000	44.019000000	2.974000000
F	-0.280000000	42.176000000	1.654000000
C	2.268000000	46.792000000	5.672000000
C	2.203000000	47.144000000	4.307000000
H	2.775000000	46.565000000	3.585000000
C	1.430000000	48.231000000	3.882000000
H	1.396000000	48.488000000	2.823000000
C	0.715000000	48.995000000	4.813000000
H	0.123000000	49.848000000	4.483000000
C	0.771000000	48.657000000	6.173000000
H	0.222000000	49.246000000	6.905000000
C	1.533000000	47.564000000	6.598000000
H	1.578000000	47.305000000	7.654000000
C	4.507000000	46.833000000	8.010000000
O	4.871000000	46.776000000	9.193000000
N	4.484000000	48.020000000	7.290000000
H	4.153000000	47.936000000	6.337000000
C	4.538000000	49.343000000	7.765000000
C	4.059000000	50.352000000	6.903000000
H	3.650000000	50.076000000	5.931000000
C	4.086000000	51.690000000	7.302000000
H	3.711000000	52.458000000	6.625000000
C	4.583000000	52.043000000	8.565000000
H	4.603000000	53.086000000	8.877000000
C	5.053000000	51.037000000	9.420000000
H	5.442000000	51.298000000	10.404000000
C	5.041000000	49.691000000	9.034000000
H	5.403000000	48.909000000	9.692000000
C	5.578000000	43.740000000	8.279000000
H	5.770000000	42.715000000	7.933000000
C	6.931000000	44.482000000	8.251000000
H	7.660000000	43.946000000	8.874000000
H	6.824000000	45.497000000	8.646000000
H	7.327000000	44.539000000	7.229000000
C	5.017000000	43.642000000	9.716000000
H	4.077000000	43.073000000	9.735000000
H	4.824000000	44.647000000	10.103000000
H	5.740000000	43.133000000	10.368000000

Complex **VIII**

O	2.849000000	17.785000000	-3.465000000
N	4.552000000	19.183000000	-6.308000000
N	2.883000000	17.717000000	-5.793000000
N	1.537000000	11.136000000	-7.843000000
N	2.998000000	9.946000000	-9.079000000
N	1.182000000	11.014000000	-9.121000000
N	2.027000000	10.309000000	-9.888000000
C	4.490000000	19.082000000	-4.834000000
C	4.359000000	20.482000000	-4.160000000
C	5.837000000	18.527000000	-4.294000000
C	6.717000000	19.781000000	-4.208000000
C	5.765000000	20.827000000	-3.593000000
C	3.325000000	18.135000000	-4.542000000
C	0.943000000	26.395000000	-1.997000000
H	0.914000000	26.351000000	-3.084000000
C	1.838000000	25.615000000	-1.260000000
H	2.538000000	24.971000000	-1.782000000
F	-0.767000000	28.039000000	-2.031000000
C	2.355000000	22.528000000	0.102000000
C	2.608000000	22.275000000	-1.261000000
H	3.368000000	22.855000000	-1.775000000
C	1.918000000	21.263000000	-1.940000000
H	2.138000000	21.070000000	-2.990000000
C	0.964000000	20.490000000	-1.266000000
H	0.433000000	19.696000000	-1.791000000
C	0.699000000	20.738000000	0.089000000
H	-0.040000000	20.137000000	0.618000000
C	1.389000000	21.747000000	0.768000000
H	1.203000000	21.930000000	1.825000000
C	5.008000000	22.000000000	1.917000000
O	5.856000000	21.829000000	2.808000000
N	4.667000000	20.998000000	1.027000000
H	3.985000000	21.241000000	0.319000000
C	5.172000000	19.697000000	0.906000000
C	4.571000000	18.878000000	-0.076000000
H	3.733000000	19.267000000	-0.656000000
C	5.061000000	17.594000000	-0.318000000
H	4.592000000	16.981000000	-1.087000000
C	6.147000000	17.098000000	0.417000000
H	6.530000000	16.096000000	0.227000000
C	6.726000000	17.905000000	1.406000000
H	7.567000000	17.528000000	1.991000000
C	6.251000000	19.196000000	1.662000000
H	6.703000000	19.827000000	2.421000000
C	5.823000000	24.934000000	3.160000000
H	5.779000000	26.027000000	3.255000000
C	7.202000000	24.612000000	2.545000000
H	8.001000000	24.982000000	3.202000000
H	7.311000000	25.101000000	1.567000000
H	7.324000000	23.533000000	2.423000000
C	5.665000000	24.362000000	4.585000000
H	6.467000000	24.745000000	5.233000000
H	5.717000000	23.269000000	4.558000000
H	4.699000000	24.660000000	5.018000000
O	1.864000000	34.715000000	8.494000000
C	1.052000000	35.455000000	7.839000000
O	0.563000000	35.138000000	6.701000000
C	0.952000000	36.922000000	8.251000000
H	-0.062000000	37.320000000	8.123000000
H	1.272000000	37.058000000	9.292000000
C	1.903000000	37.664000000	7.283000000
H	1.449000000	37.664000000	6.284000000
O	3.142000000	36.882000000	7.075000000
H	3.519000000	36.736000000	7.961000000
C	2.228000000	39.089000000	7.723000000
H	2.732000000	39.073000000	8.703000000
H	1.271000000	39.615000000	7.864000000
C	3.104000000	39.873000000	6.728000000
H	2.634000000	39.799000000	5.724000000
O	4.439000000	39.367000000	6.688000000
H	4.354000000	38.412000000	6.520000000
C	3.189000000	41.353000000	7.103000000
H	3.573000000	41.449000000	8.128000000
H	2.179000000	41.784000000	7.087000000
C	4.080000000	42.147000000	6.138000000
H	5.126000000	41.843000000	6.238000000
H	3.783000000	41.944000000	5.104000000
N	3.959000000	43.592000000	6.341000000
C	3.064000000	44.377000000	5.612000000

H	2.765000000	24.761000000	1.965000000
H	1.213000000	24.824000000	1.143000000
C	2.897000000	24.730000000	-0.198000000
H	2.457000000	25.256000000	-1.049000000
H	3.975000000	24.946000000	-0.199000000
N	2.689000000	23.290000000	-0.407000000
C	1.802000000	22.750000000	-1.346000000
C	1.806000000	21.359000000	-1.182000000
C	2.721000000	21.062000000	-0.105000000
C	3.259000000	22.276000000	0.354000000
C	0.984000000	23.583000000	-2.247000000
C	0.064000000	24.534000000	-1.753000000
H	-0.043000000	24.676000000	-0.681000000
C	-0.720000000	25.303000000	-2.617000000
H	-1.423000000	26.042000000	-2.239000000
C	-0.600000000	25.098000000	-3.994000000
C	0.296000000	24.170000000	-4.525000000
H	0.372000000	24.049000000	-5.604000000
C	1.092000000	23.430000000	-3.645000000
H	1.829000000	22.736000000	-4.043000000
F	-1.369000000	25.831000000	-4.838000000
C	0.837000000	20.403000000	-1.769000000
C	0.730000000	20.154000000	-3.150000000
H	1.406000000	20.650000000	-3.840000000
C	-0.214000000	19.247000000	-3.648000000
H	-0.268000000	19.068000000	-4.723000000
C	-1.069000000	18.566000000	-2.775000000
H	-1.804000000	17.859000000	-3.163000000
C	-0.972000000	18.799000000	-1.396000000
H	-1.637000000	18.280000000	-0.704000000
C	-0.030000000	19.704000000	-0.900000000
H	0.039000000	19.893000000	0.170000000
C	3.023000000	19.734000000	0.498000000
O	3.242000000	19.600000000	1.710000000
N	3.073000000	18.656000000	-0.371000000
H	2.966000000	18.819000000	-1.369000000
C	3.426000000	17.336000000	0.000000000
C	4.114000000	16.539000000	-0.935000000
H	4.329000000	16.944000000	-1.919000000
C	4.494000000	15.234000000	-0.610000000
H	5.032000000	14.637000000	-1.349000000
C	4.203000000	14.703000000	0.653000000
H	4.514000000	13.692000000	0.913000000
C	3.508000000	15.493000000	1.579000000
H	3.265000000	15.090000000	2.563000000
C	3.113000000	16.798000000	1.265000000
H	2.584000000	17.405000000	1.989000000
C	4.344000000	22.564000000	1.371000000
H	4.643000000	23.612000000	1.229000000
C	5.606000000	21.711000000	1.120000000
H	6.411000000	22.028000000	1.797000000
H	5.959000000	21.825000000	0.086000000
H	5.402000000	20.652000000	1.309000000
C	3.861000000	22.412000000	2.833000000
H	4.655000000	22.729000000	3.524000000
H	3.602000000	21.368000000	3.023000000
H	2.970000000	23.024000000	3.031000000
O	2.810000000	34.145000000	7.429000000
C	1.865000000	34.978000000	7.174000000
O	1.040000000	34.815000000	6.208000000
C	1.790000000	36.264000000	7.988000000
H	0.744000000	36.526000000	8.185000000
H	2.312000000	36.126000000	8.945000000
C	2.454000000	37.398000000	7.183000000
H	1.864000000	37.564000000	6.270000000
O	3.751000000	36.963000000	6.689000000
C	3.649000000	18.388000000	-6.779000000
C	3.346000000	18.237000000	-8.240000000
C	1.851000000	16.687000000	-5.959000000
C	2.115000000	19.056000000	-8.687000000
C	2.382000000	15.267000000	-5.865000000
C	1.792000000	18.855000000	-10.175000000
C	2.223000000	14.527000000	-4.682000000
C	3.022000000	14.654000000	-6.957000000
C	0.591000000	19.685000000	-10.644000000
C	2.655000000	13.197000000	-4.610000000
C	3.470000000	13.335000000	-6.880000000
C	3.272000000	12.573000000	-5.711000000
C	3.747000000	11.167000000	-5.623000000
C	3.536000000	10.206000000	-6.650000000
C	4.492000000	10.774000000	-4.493000000
C	4.112000000	8.925000000	-6.540000000
C	5.037000000	9.491000000	-4.381000000
C	2.698000000	10.455000000	-7.835000000
C	4.860000000	8.565000000	-5.417000000
H	0.329000000	11.420000000	-9.488000000
H	3.611000000	20.447000000	-3.360000000
H	4.022000000	21.204000000	-4.912000000
H	6.230000000	17.732000000	-4.939000000
H	5.666000000	18.114000000	-3.290000000
H	7.013000000	20.083000000	-5.222000000
H	7.627000000	19.623000000	-3.615000000
H	5.753000000	20.722000000	-2.498000000
H	6.064000000	21.858000000	-3.820000000
H	3.193000000	17.179000000	-8.493000000
H	4.234000000	18.578000000	-8.786000000
H	1.360000000	16.851000000	-6.925000000
H	1.115000000	16.863000000	-5.166000000
H	2.310000000	20.119000000	-8.482000000
H	1.237000000	18.782000000	-8.081000000
H	1.599000000	17.785000000	-10.360000000
H	2.680000000	19.113000000	-10.774000000
H	1.755000000	14.996000000	-3.817000000
H	3.171000000	15.208000000	-7.884000000
H	0.382000000	19.519000000	-11.709000000
H	0.774000000	20.760000000	-10.500000000
H	-0.315000000	19.424000000	-10.080000000
H	2.487000000	12.625000000	-3.697000000
H	3.975000000	12.884000000	-7.733000000
H	4.671000000	11.509000000	-3.709000000
H	3.947000000	8.211000000	-7.345000000
H	5.612000000	9.222000000	-3.495000000
H	5.291000000	7.568000000	-5.347000000
Ca	2.235000000	32.909000000	5.493000000
O	1.319000000	30.898000000	4.658000000
C	2.444000000	30.577000000	4.104000000
O	3.429000000	31.389000000	4.093000000
C	2.615000000	29.176000000	3.517000000
H	3.337000000	29.238000000	2.692000000
H	3.090000000	28.571000000	4.308000000
C	1.304000000	28.475000000	3.092000000
H	0.872000000	29.046000000	2.240000000
O	0.377000000	28.423000000	4.168000000
H	0.347000000	29.340000000	4.512000000
C	1.507000000	27.013000000	2.666000000
H	0.504000000	26.567000000	2.630000000
H	2.055000000	26.499000000	3.471000000
C	2.198000000	26.723000000	1.321000000
H	3.233000000	27.120000000	1.330000000
O	1.474000000	27.274000000	0.208000000
H	1.513000000	28.238000000	0.284000000
C	2.244000000	25.205000000	1.111000000

H	4.164000000	36.437000000	7.394000000
C	2.555000000	38.721000000	7.959000000
H	3.142000000	38.580000000	8.881000000
H	1.538000000	39.021000000	8.258000000
C	3.203000000	39.834000000	7.112000000
H	2.672000000	39.854000000	6.135000000
O	4.595000000	39.589000000	6.915000000
H	4.648000000	38.658000000	6.623000000
C	3.081000000	41.237000000	7.716000000
H	3.564000000	41.264000000	8.703000000
H	2.021000000	41.481000000	7.865000000
C	3.729000000	42.253000000	6.759000000
H	4.819000000	42.163000000	6.807000000
H	3.439000000	42.008000000	5.732000000
N	3.355000000	43.659000000	6.964000000
C	2.594000000	44.380000000	6.032000000
C	2.727000000	45.732000000	6.338000000
C	3.569000000	45.828000000	7.506000000
C	3.956000000	44.527000000	7.864000000
C	1.733000000	43.731000000	5.033000000
C	0.830000000	42.708000000	5.402000000
H	0.762000000	42.412000000	6.446000000
C	-0.002000000	42.094000000	4.462000000
H	-0.701000000	41.310000000	4.746000000
C	0.052000000	42.527000000	3.136000000
C	0.914000000	43.549000000	2.732000000
H	0.932000000	43.861000000	1.690000000
C	1.756000000	44.135000000	3.681000000
H	2.448000000	44.914000000	3.372000000
F	-0.757000000	41.943000000	2.218000000
C	2.006000000	46.850000000	5.679000000
C	2.475000000	47.411000000	4.474000000
H	3.375000000	47.000000000	4.014000000
C	1.811000000	48.491000000	3.878000000
H	2.190000000	48.916000000	2.949000000
C	0.663000000	49.024000000	4.477000000
H	0.144000000	49.863000000	4.015000000
C	0.188000000	48.476000000	5.677000000
H	-0.704000000	48.888000000	6.149000000
C	0.857000000	47.403000000	6.275000000
H	0.496000000	46.982000000	7.212000000
C	3.978000000	47.083000000	8.192000000
O	4.123000000	47.189000000	9.416000000
N	4.214000000	48.127000000	7.310000000
H	4.093000000	47.905000000	6.329000000
C	4.650000000	49.439000000	7.564000000
C	5.050000000	50.207000000	6.450000000
H	5.007000000	49.765000000	5.453000000
C	5.505000000	51.515000000	6.618000000
H	5.819000000	52.089000000	5.746000000
C	5.564000000	52.083000000	7.898000000
H	5.921000000	53.104000000	8.032000000
C	5.159000000	51.322000000	9.002000000
H	5.195000000	51.754000000	10.003000000
C	4.704000000	50.007000000	8.853000000
H	4.401000000	49.411000000	9.706000000
C	4.873000000	44.027000000	8.955000000
H	5.099000000	42.975000000	8.732000000
C	6.223000000	44.774000000	8.988000000
H	6.893000000	44.296000000	9.715000000
H	6.085000000	45.818000000	9.287000000
H	6.711000000	44.751000000	8.004000000
C	4.169000000	44.068000000	10.330000000
H	3.243000000	43.476000000	10.314000000
H	3.920000000	45.104000000	10.583000000
H	4.829000000	43.655000000	11.106000000
